# Supplementary material for: Exploring the causes underlying the latitudinal variation in range sizes: Evidence for Rapoport’s rule in spiny lizards (genus Sceloporus)
Source: PLoS One. 2024 Jul 9;19(7):e0306832. doi: 10.1371/journal.pone.0306832 (PMC11233011; doi:10.1371/journal.pone.0306832)
Supplement: S1 Appendix — This table contains the raw values from all the combinations between environmental hypotheses used for model selection. (PDF) [file pone.0306832.s001.pdf]

S1 Appendix. Results of model selection based on AIC for climatic hypothesis

Species level

All species – OLS

(Intercept)	CCV	CEH min	CVH	Elevation	df	logLik	AIC	delta	weight
3.71E-17	-	-0.62	-	-0.32	4.00	-126.79	261.59	0.00	0.20
3.75E-17	0.16	-0.50	-	-0.21	5.00	-125.92	261.83	0.24	0.18
-9.99E-18	0.25	-	0.38	-	4.00	-127.05	262.10	0.51	0.15
8.10E-17	-	-1.03	-0.39	-0.42	5.00	-126.40	262.80	1.21	0.11
2.62E-17	0.30	-0.35	-	-	4.00	-127.42	262.84	1.25	0.11
6.73E-17	0.14	-0.80	-0.26	-0.29	6.00	-125.74	263.49	1.90	0.08
-9.70E-18	0.22	-	0.41	-0.05	5.00	-126.92	263.84	2.25	0.06
-2.31E-19	0.26	-0.10	0.28	-	5.00	-126.98	263.96	2.37	0.06
-2.62E-17	-	-	0.54	-0.15	4.00	-128.69	265.38	3.79	0.03
-3.75E-17	-	-	0.51	-	3.00	-130.26	266.52	4.94	0.02
-4.45E-17	-	0.08	0.59	-	4.00	-130.22	268.43	6.84	0.01
6.30E-18	-	-0.48	-	-	3.00	-132.44	270.88	9.29	0.00
3.00E-17	0.45	-	-	-	3.00	-134.27	274.53	12.94	0.00
2.23E-17	0.49	-	-	0.13	4.00	-133.35	274.71	13.12	0.00
-4.10E-18	-	-	-	-	2.00	-145.65	295.30	33.71	0.00
-5.61E-19	-	-	-	-0.04	3.00	-145.56	297.13	35.54	0.00

Tonini – OLS

(Intercept)	CCV	CEH min	CVH	Elevation	df	logLik	AIC	delta	weight
1.57778E-17	-	-0.62	-	-0.31	4.00	-112.76	233.51	0.00	0.20
-9.58935E-17	0.23	-	0.40	-	4.00	-112.85	233.69	0.18	0.18
-1.88719E-17	0.14	-0.52	-	-0.22	5.00	-112.15	234.29	0.78	0.13
-7.81679E-17	0.19	-	0.44	-0.07	5.00	-112.63	235.26	1.75	0.08
-8.35764E-17	0.28	-0.36	-	-	4.00	-113.67	235.33	1.82	0.08
3.43315E-17	-	-0.81	-0.18	-0.36	5.00	-112.69	235.38	1.87	0.08
-4.49485E-17	-	-	0.55	-0.16	4.00	-113.81	235.62	2.11	0.07
-9.63613E-17	0.23	0.01	0.42	-	5.00	-112.84	235.69	2.18	0.07
-1.30827E-17	0.14	-0.57	-0.05	-0.23	6.00	-112.14	236.29	2.77	0.05
-7.7407E-17	-	-	0.52	-	3.00	-115.32	236.64	3.12	0.04
-8.62735E-17	-	0.19	0.71	-	4.00	-115.12	238.23	4.72	0.02
-5.50106E-17	-	-0.48	-	-	3.00	-117.78	241.56	8.04	0.00
-1.12935E-16	0.44	-	-	-	3.00	-120.22	246.43	12.92	0.00
-1.41316E-16	0.48	-	-	0.12	4.00	-119.50	247.00	13.48	0.00

-7.7407E-17	-	-	-	-	2.00	-130.04	264.08	30.57	0.00
-6.68607E-17	-	-	-	-0.05	3.00	-129.92	265.84	32.33	0.00

Leache – OLS

(Intercept)	CCV	CEH min	CVH	Elevation	df	logLik	AIC	delta	weight
-1.01075E-16	-	-0.57	-	-0.29	4.00	-100.84	209.68	0.00	0.17
6.66058E-18	0.25	-	0.35	-	4.00	-100.86	209.72	0.04	0.17
-5.73931E-17	0.29	-0.32	-	-	4.00	-101.03	210.06	0.38	0.14
-7.82106E-17	0.17	-0.45	-	-0.18	5.00	-100.09	210.18	0.50	0.13
-1.80693E-16	-	-0.98	-0.39	-0.39	5.00	-100.54	211.09	1.41	0.08
6.7909E-18	0.22	-	0.37	-0.05	5.00	-100.78	211.55	1.88	0.07
-1.55064E-17	0.26	-0.12	0.23	-	5.00	-100.80	211.59	1.91	0.07
-1.35473E-16	0.15	-0.75	-0.27	-0.26	6.00	-99.95	211.91	2.23	0.06
6.33996E-18	-	-	0.51	-0.14	4.00	-102.16	212.32	2.64	0.05
5.54542E-18	-	-	0.49	-	3.00	-103.22	212.43	2.75	0.04
1.594E-17	-	0.05	0.54	-	4.00	-103.20	214.40	4.72	0.02
-8.94846E-17	-	-0.46	-	-	3.00	-104.60	215.20	5.52	0.01
-2.19481E-17	0.45	-	-	-	3.00	-105.35	216.69	7.01	0.01
-1.83235E-17	0.48	-	-	0.10	4.00	-104.92	217.85	8.17	0.00
-5.51257E-17	-	-	-	-	2.00	-114.43	232.86	23.18	0.00
-5.59462E-17	-	-	-	-0.07	3.00	-114.25	234.50	24.82	0.00

Tonini – PGLS

(Intercept)	CCV	CEH min	CVH	Elevation	df	logLik	AIC	delta	weight
-9.86067E-08	-	-0.62	-	-0.31	3.00	-112.76	231.51	0.00	0.20
3.55889E-08	0.23	-	0.40	-	3.00	-112.85	231.69	0.18	0.18
5.99281E-08	0.14	-0.52	-	-0.22	4.00	-112.15	232.29	0.78	0.13
-1.0424E-07	0.19	-	0.44	-0.07	4.00	-112.63	233.26	1.75	0.08
3.81597E-07	0.28	-0.36	-	-	3.00	-113.67	233.33	1.82	0.08
-1.12745E-08	-	-0.81	-0.18	-0.36	4.00	-112.69	233.38	1.87	0.08
-3.73152E-07	-	-	0.55	-0.16	3.00	-113.81	233.62	2.11	0.07
2.4521E-08	0.23	0.01	0.42	-	4.00	-112.84	233.69	2.18	0.07
8.01128E-08	0.14	-0.57	-0.05	-0.23	5.00	-112.14	234.29	2.77	0.05
-1.2215E-07	-	-	0.52	-	2.00	-115.32	234.64	3.12	0.04
-2.85257E-07	-	0.19	0.71	-	3.00	-115.12	236.23	4.72	0.02
2.88443E-07	-	-0.48	-	-	2.00	-117.78	239.56	8.04	0.00
1.74172E-07	0.44	-	-	-	2.00	-120.22	244.43	12.92	0.00
3.97425E-07	0.48	-	-	0.12	3.00	-119.50	245.00	13.48	0.00

-1.34243E-07	-	-	-	-	1.00	-130.04	262.08	30.57	0.00
-2.16021E-07	-	-	-	-0.05	2.00	-129.92	263.84	32.33	0.00

Leache – PGLS

(Intercept)	CCV	CEH min	CVH	Elevation	df	logLik	AIC	delta	weight
9.70651E-07	-	-0.57	-	-0.29	3.00	-100.84	207.68	0.00	0.17
1.07217E-06	0.25	-	0.35	-	3.00	-100.86	207.72	0.04	0.17
1.36603E-06	0.29	-0.32	-	-	3.00	-101.03	208.06	0.38	0.14
1.08272E-06	0.17	-0.45	-	-0.18	4.00	-100.09	208.18	0.50	0.13
1.10693E-06	-	-0.98	-0.39	-0.39	4.00	-100.54	209.09	1.41	0.08
9.60656E-07	0.22	-	0.37	-0.05	4.00	-100.78	209.55	1.88	0.07
1.18623E-06	0.26	-0.12	0.23	-	4.00	-100.80	209.59	1.91	0.07
1.1666E-06	0.15	-0.75	-0.27	-0.26	5.00	-99.95	209.91	2.23	0.06
7.51499E-07	-	-	0.51	-0.14	3.00	-102.16	210.32	2.64	0.05
1.11361E-06	-	-	0.49	-	2.00	-103.22	210.43	2.75	0.04
1.05946E-06	-	0.05	0.54	-	3.00	-103.20	212.40	4.72	0.02
1.51484E-06	-	-0.46	-	-	2.00	-104.60	213.20	5.52	0.01
7.35762E-07	0.45	-	-	-	2.00	-105.35	214.69	7.01	0.01
1.02004E-06	0.48	-	-	0.10	3.00	-104.92	215.85	8.17	0.00
4.88716E-07	-	-	-	-	1.00	-114.43	230.86	23.18	0.00
3.05745E-07	-	-	-	-0.07	2.00	-114.25	232.50	24.82	0.00

Sites level

All species – OLS

(Intercept)	CCV	CEH min	CVH	Elevation	df	logLik	AIC	delta	weight
-4.92547E-16	0.21	-0.84	-0.08	-0.43	6.00	-1995.44	4002.87	0.00	0.98
-6.26102E-16	0.21	-0.77	-	-0.42	5.00	-2000.54	4011.07	8.20	0.02
-4.02832E-16	-	-0.97	-0.07	-0.59	5.00	-2081.62	4173.23	170.36	0.00
-5.24551E-16	-	-0.90	-	-0.58	4.00	-2085.57	4179.15	176.28	0.00
-9.26059E-16	0.52	-0.46	0.05	-	5.00	-2308.28	4626.56	623.69	0.00
-8.37315E-16	0.52	-0.51	-	-	4.00	-2310.33	4628.65	625.78	0.00
-1.80398E-15	0.37	-	0.57	-0.16	5.00	-2406.44	4822.87	820.00	0.00
-1.71675E-15	0.49	-	0.48	-	4.00	-2457.76	4923.51	920.64	0.00
-2.00659E-15	-	-	0.77	-0.41	4.00	-2625.94	5259.87	1257.00	0.00
-1.33161E-15	0.80	-	-	0.21	4.00	-3029.60	6067.20	2064.33	0.00
-1.36541E-15	-	-0.32	0.39	-	4.00	-3086.67	6181.35	2178.47	0.00
-1.36185E-15	0.69	-	-	-	3.00	-3110.70	6227.40	2224.53	0.00

-1.9081E-15	-	-	0.68	-	3.00	-3130.45	6266.90	2264.03	0.00
-7.15315E-16	-	-0.67	-	-	3.00	-3151.47	6308.94	2306.06	0.00
-1.45909E-15	-	-	-	-0.24	3.00	-3925.58	7857.15	3854.28	0.00
-1.4392E-15	-	-	-	-	2.00	-4008.00	8020.00	4017.13	0.00

Tonini – OLS

(Intercept)	CCV	CEH min	CVH	Elevation	df	logLik	AIC	delta	weight
1.80028E-16	0.22	-0.85	-0.09	-0.42	6.00	-2026.94	4065.88	0.00	1.00
2.1958E-17	0.22	-0.76	-	-0.41	5.00	-2033.92	4077.85	11.97	0.00
2.7444E-16	-	-0.98	-0.08	-0.59	5.00	-2120.04	4250.09	184.21	0.00
1.28754E-16	-	-0.89	-	-0.58	4.00	-2125.56	4259.11	193.23	0.00
-1.83681E-16	0.52	-0.50	-	-	4.00	-2322.91	4653.82	587.94	0.00
-2.44291E-16	0.52	-0.47	0.04	-	5.00	-2321.97	4653.93	588.05	0.00
-1.1354E-15	0.38	-	0.56	-0.15	5.00	-2432.19	4874.38	808.50	0.00
-1.0535E-15	0.50	-	0.48	-	4.00	-2476.72	4961.44	895.56	0.00
-1.34424E-15	-	-	0.77	-0.41	4.00	-2660.45	5328.90	1263.02	0.00
-6.73255E-16	0.80	-	-	0.21	4.00	-3024.73	6057.45	1991.57	0.00
-6.87248E-16	-	-0.33	0.37	-	4.00	-3104.35	6216.71	2150.83	0.00
-7.03776E-16	0.69	-	-	-	3.00	-3107.57	6221.14	2155.26	0.00
-1.24627E-15	-	-	0.67	-	3.00	-3150.20	6306.39	2240.51	0.00
-6.1159E-17	-	-0.67	-	-	3.00	-3163.82	6333.64	2267.76	0.00
-8.0104E-16	-	-	-	-0.24	3.00	-3926.18	7858.36	3792.48	0.00
-7.81219E-16	-	-	-	-	2.00	-4008.00	8020.00	3954.12	0.00

Leache – OLS

(Intercept)	CCV	CEH min	CVH	Elevation	df	logLik	AIC	delta	weight
-1.27344E-15	0.22	-0.80	-0.05	-0.42	6.00	-2031.27	4074.54	0.00	0.70
-1.35437E-15	0.22	-0.75	-	-0.42	5.00	-2033.10	4076.19	1.66	0.30
-1.1795E-15	-	-0.93	-0.04	-0.59	5.00	-2123.20	4256.40	181.87	0.00
-1.24779E-15	-	-0.89	-	-0.59	4.00	-2124.41	4256.83	182.29	0.00
-1.70125E-15	0.52	-0.42	0.08	-	5.00	-2329.86	4669.72	595.18	0.00
-1.56484E-15	0.53	-0.49	-	-	4.00	-2334.62	4677.23	602.70	0.00
-2.51693E-15	0.37	-	0.57	-0.17	5.00	-2397.54	4805.09	730.55	0.00
-2.4253E-15	0.50	-	0.48	-	4.00	-2454.41	4916.83	842.29	0.00
-2.7203E-15	-	-	0.77	-0.42	4.00	-2619.86	5247.72	1173.19	0.00
-2.04691E-15	0.80	-	-	0.20	4.00	-3018.85	6045.70	1971.16	0.00
-2.07569E-15	0.69	-	-	-	3.00	-3093.02	6192.05	2117.51	0.00
-2.14541E-15	-	-0.28	0.42	-	4.00	-3112.15	6232.30	2157.76	0.00
-2.61963E-15	-	-	0.68	-	3.00	-3145.11	6296.21	2221.67	0.00

-1.44073E-15	-	-0.66	-	-	3.00	-3186.66	6379.32	2304.79	0.00
-2.17427E-15	-	-	-	-0.25	3.00	-3918.59	7843.17	3768.63	0.00
-2.15357E-15	-	-	-	-	2.00	-4008.00	8020.00	3945.47	0.00
