# Supplementary material for: Exploring the causes underlying the latitudinal variation in range sizes: Evidence for Rapoport’s rule in spiny lizards (genus Sceloporus)
Source: PLoS One. 2024 Jul 9;19(7):e0306832. doi: 10.1371/journal.pone.0306832 (PMC11233011; doi:10.1371/journal.pone.0306832)

S2 Appendix. Results of simultaneous autoregressive models

Rapoport's rule

All species

Scheme	Model	R2	AIC	p-value	Intercept	Latitude
W	error	0.96	-1344.77	0.00	0.02	0.68
S	error	0.95	-566.01	0.00	-0.22	0.67
C	error	0.93	411.60	0.00	-0.22	0.68
C	lm	0.51	5982.71	0.00	0.00	0.72
S	lm	0.51	5982.71	0.00	0.00	0.72
W	lm	0.51	5982.71	0.00	0.00	0.72

Tonini

Scheme	Model	R2	AIC	p-value	Intercept	Latitude
W	error	0.96	-1367.95	0.00	0.02	0.68
S	error	0.95	-561.46	0.00	-0.23	0.68
C	error	0.93	439.06	0.00	-0.23	0.69
C	lm	0.50	6034.48	0.00	0.00	0.71
W	lm	0.50	6034.48	0.00	0.00	0.71
S	lm	0.50	6034.48	0.00	0.00	0.71

Leache

Scheme	Model	R2	AIC	p-value	Intercept	Latitude
W	error	0.96	-1060.81	0.00	0.00	0.70
S	error	0.95	-319.44	0.00	-0.28	0.71
C	error	0.93	628.35	0.00	-0.27	0.71
C	lm	0.50	6061.82	0.00	0.00	0.71
S	lm	0.50	6061.82	0.00	0.00	0.71
W	lm	0.50	6061.82	0.00	0.00	0.71

Moran's correlograms

All species – W scheme **All species – S scheme** **All species – C scheme**

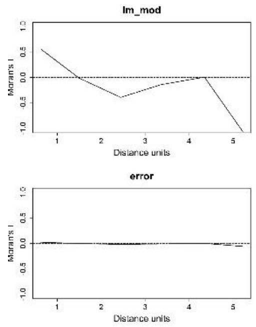

Tonini – W scheme

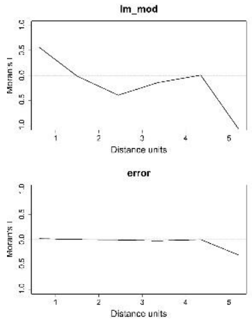

Tonini – S scheme

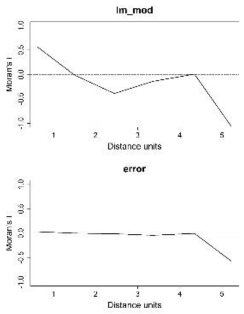

Tonini – C scheme

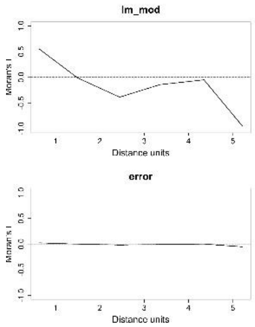

Leache – W scheme

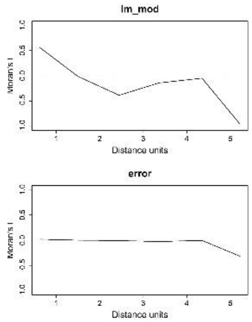

Leache – S scheme

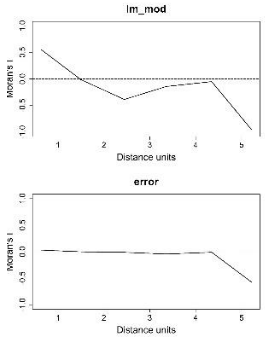

Leache – C scheme

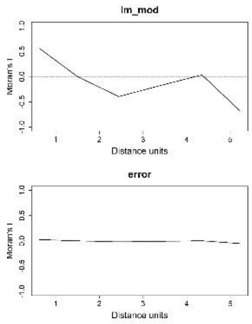

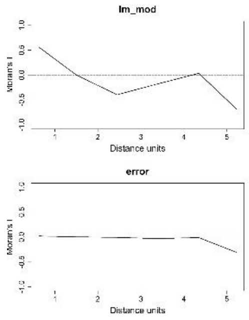

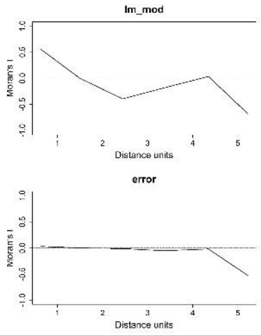

Climatic hypothesis

All species - SARs

Scheme	Model	R2	AIC	p-value	Intercept	CCV	Elevation	CEH min	CVH
W	error	0.96	-1287.22	0.00	0.02	0.02	-0.14	-0.31	-0.08
S	error	0.95	-608.29	0.00	-0.31	0.11	-0.38	-0.91	-0.27
C	error	0.94	102.39	0.00	-0.36	0.21	-0.47	-1.06	-0.39
C	lm	0.76	4002.87	0.00	0.00	0.21	-0.43	-0.84	-0.08
S	lm	0.76	4002.87	0.00	0.00	0.21	-0.43	-0.84	-0.08
W	lm	0.76	4002.87	0.00	0.00	0.21	-0.43	-0.84	-0.08

Tonini - SARs

Scheme	Model	R2	AIC	p-value	Intercept	CCV	Elevation	CEH min	CVH
W	error	0.96	-1309.75	0.00	0.02	0.02	-0.12	-0.30	-0.07
S	error	0.95	-601.97	0.00	-0.32	0.11	-0.38	-0.92	-0.27
C	error	0.94	129.02	0.00	-0.37	0.21	-0.46	-1.07	-0.39
W	lm	0.75	4065.88	0.00	0.00	0.22	-0.42	-0.85	-0.09
C	lm	0.75	4065.88	0.00	0.00	0.22	-0.42	-0.85	-0.09
S	lm	0.75	4065.88	0.00	0.00	0.22	-0.42	-0.85	-0.09

Leache – SARs

Scheme	Model	R2	AIC	p-value	Intercept	CCV	Elevation	CEH min	CVH
W	error	0.96	-998.15	0.00	0.01	0.02	-0.13	-0.35	-0.05
S	error	0.95	-355.61	0.00	-0.35	0.12	-0.37	-0.94	-0.28
C	error	0.93	324.76	0.00	-0.41	0.23	-0.47	-1.09	-0.41
C	lm	0.75	4074.54	0.00	0.00	0.22	-0.42	-0.80	-0.05
S	lm	0.75	4074.54	0.00	0.00	0.22	-0.42	-0.80	-0.05
W	lm	0.75	4074.54	0.00	0.00	0.22	-0.42	-0.80	-0.05

Moran's correlograms

All species – W scheme

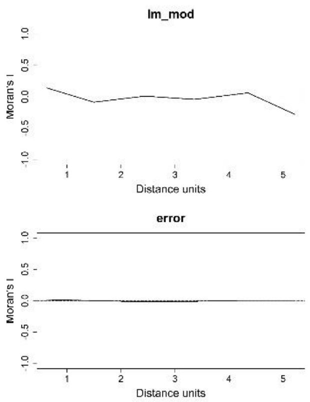

All species – S scheme

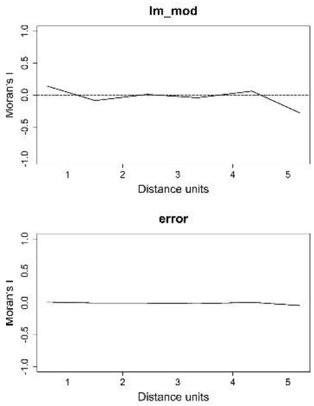

All species – C scheme

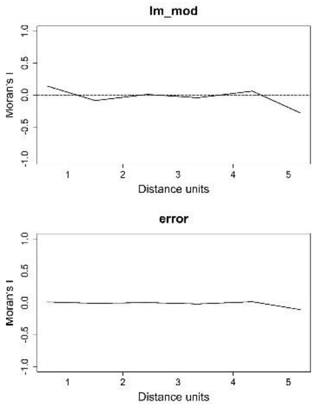

Tonini – W scheme

Tonini – S scheme

Tonini – C scheme

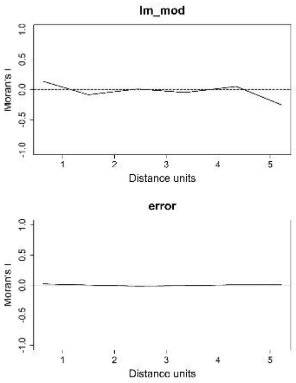

Leache – W scheme

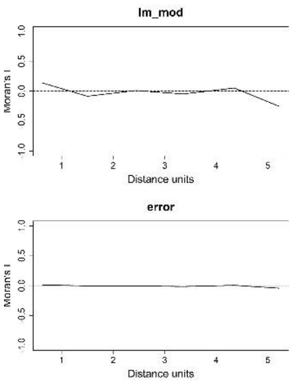

Leache – S scheme

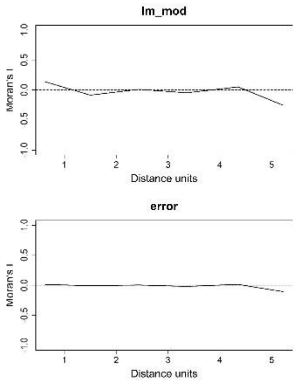

Leache – C scheme

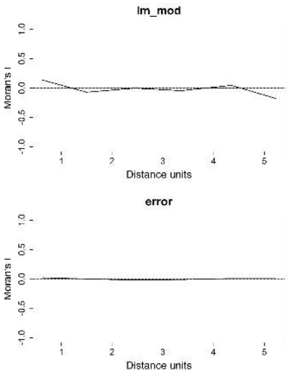

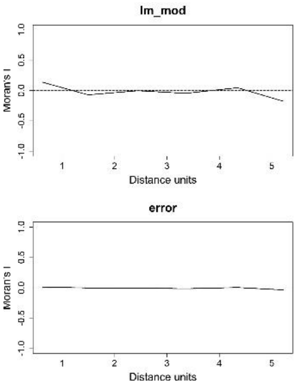

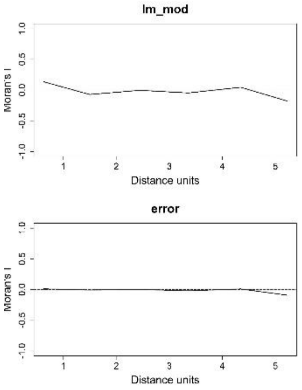

Supplement: S2 Appendix — This appendix shows the results of the simultaneous autoregressive models for Rapoport’s rule and environmental hypotheses. (PDF) [file pone.0306832.s002.pdf]
