# Supplementary material for: Exploring the causes underlying the latitudinal variation in range sizes: Evidence for Rapoport’s rule in spiny lizards (genus Sceloporus)
Source: PLoS One. 2024 Jul 9;19(7):e0306832. doi: 10.1371/journal.pone.0306832 (PMC11233011; doi:10.1371/journal.pone.0306832)
Supplement: S3 Appendix — These tables contain all the simulated coefficients for the relation between range size and latitude. (PDF) [file pone.0306832.s003.pdf]

S3 Appendix. Coefficients for null Simulations for Rapoport's rule

All species (OLS - species level)

Simulation	Latitude	R2	p-value	AIC	BIC
Sim_1	-0.04	-0.01	0.66	297.10	305.01
Sim_2	0.10	0.00	0.29	296.16	304.06
Sim_3	-0.09	0.00	0.39	296.53	304.43
Sim_4	-0.09	0.00	0.39	296.52	304.43
Sim_5	0.08	0.00	0.40	296.56	304.47
Sim_6	-0.08	0.00	0.45	296.71	304.61
Sim_7	0.13	0.01	0.20	295.63	303.53
Sim_8	0.13	0.01	0.19	295.53	303.44
Sim_9	0.13	0.01	0.21	295.66	303.57
Sim_10	-0.08	0.00	0.44	296.70	304.60
Sim_11	-0.02	-0.01	0.83	297.25	305.15
Sim_12	0.12	0.00	0.24	295.85	303.76
Sim_13	0.02	-0.01	0.84	297.25	305.16
Sim_14	0.00	-0.01	0.99	297.30	305.20
Sim_15	0.08	0.00	0.42	296.62	304.52
Sim_16	0.01	-0.01	0.94	297.29	305.20
Sim_17	0.01	-0.01	0.92	297.29	305.19
Sim_18	0.09	0.00	0.37	296.46	304.36
Sim_19	0.18	0.02	0.07	293.83	301.74
Sim_20	-0.03	-0.01	0.80	297.23	305.14
Sim_21	-0.04	-0.01	0.68	297.13	305.03
Sim_22	0.00	-0.01	0.98	297.30	305.20
Sim_23	-0.01	-0.01	0.96	297.29	305.20
Sim_24	-0.09	0.00	0.35	296.39	304.30
Sim_25	0.06	-0.01	0.52	296.88	304.79
Sim_26	0.03	-0.01	0.73	297.17	305.07
Sim_27	-0.06	-0.01	0.53	296.89	304.79
Sim_28	0.11	0.00	0.28	296.09	304.00
Sim_29	-0.05	-0.01	0.62	297.04	304.94
Sim_30	0.09	0.00	0.36	296.45	304.36
Sim_31	-0.04	-0.01	0.70	297.14	305.05
Sim_32	-0.09	0.00	0.38	296.52	304.42
Sim_33	-0.10	0.00	0.30	296.20	304.11
Sim_34	-0.03	-0.01	0.75	297.19	305.09
Sim_35	-0.02	-0.01	0.87	297.27	305.17
Sim_36	0.01	-0.01	0.93	297.29	305.19
Sim_37	-0.02	-0.01	0.87	297.27	305.17

Sim_38	0.03	-0.01	0.79	297.23	305.13
Sim_39	0.09	0.00	0.37	296.48	304.38
Sim_40	-0.05	-0.01	0.58	296.99	304.89
Sim_41	-0.12	0.01	0.21	295.71	303.62
Sim_42	0.17	0.02	0.09	294.35	302.25
Sim_43	-0.12	0.00	0.23	295.80	303.70
Sim_44	0.10	0.00	0.34	296.35	304.25
Sim_45	-0.12	0.00	0.25	295.91	303.82
Sim_46	0.08	0.00	0.41	296.59	304.49
Sim_47	0.04	-0.01	0.71	297.16	305.06
Sim_48	0.02	-0.01	0.82	297.24	305.15
Sim_49	-0.25	0.05	0.01	290.80	298.70
Sim_50	0.20	0.03	0.04	293.06	300.97
Sim_51	-0.07	-0.01	0.50	296.84	304.74
Sim_52	-0.01	-0.01	0.96	297.29	305.20
Sim_53	0.05	-0.01	0.62	297.04	304.95
Sim_54	0.02	-0.01	0.82	297.24	305.15
Sim_55	-0.04	-0.01	0.68	297.13	305.03
Sim_56	0.07	0.00	0.46	296.73	304.63
Sim_57	-0.12	0.01	0.21	295.71	303.62
Sim_58	0.05	-0.01	0.62	297.05	304.95
Sim_59	0.02	-0.01	0.84	297.26	305.16
Sim_60	-0.03	-0.01	0.79	297.22	305.13
Sim_61	0.17	0.02	0.08	294.24	302.14
Sim_62	0.06	-0.01	0.55	296.94	304.84
Sim_63	0.06	-0.01	0.52	296.86	304.77
Sim_64	0.05	-0.01	0.58	296.99	304.89
Sim_65	0.13	0.01	0.18	295.48	303.38
Sim_66	0.21	0.03	0.03	292.70	300.61
Sim_67	0.07	-0.01	0.51	296.84	304.75
Sim_68	-0.07	0.00	0.47	296.77	304.67
Sim_69	-0.05	-0.01	0.64	297.07	304.97
Sim_70	-0.01	-0.01	0.94	297.29	305.20
Sim_71	-0.18	0.02	0.07	294.03	301.93
Sim_72	0.02	-0.01	0.81	297.24	305.14
Sim_73	0.07	-0.01	0.50	296.83	304.73
Sim_74	-0.14	0.01	0.17	295.40	303.30
Sim_75	-0.05	-0.01	0.60	297.02	304.93
Sim_76	-0.01	-0.01	0.88	297.27	305.18
Sim_77	0.04	-0.01	0.68	297.12	305.02
Sim_78	-0.02	-0.01	0.87	297.27	305.17

Sim_79	-0.03	-0.01	0.75	297.20	305.10
Sim_80	-0.26	0.06	0.01	290.17	298.08
Sim_81	-0.16	0.02	0.11	294.64	302.55
Sim_82	0.01	-0.01	0.92	297.29	305.19
Sim_83	0.06	-0.01	0.56	296.95	304.85
Sim_84	0.07	0.00	0.46	296.73	304.64
Sim_85	-0.07	0.00	0.48	296.79	304.69
Sim_86	-0.14	0.01	0.15	295.16	303.07
Sim_87	-0.03	-0.01	0.74	297.19	305.09
Sim_88	0.08	0.00	0.40	296.58	304.48
Sim_89	0.02	-0.01	0.88	297.27	305.18
Sim_90	-0.01	-0.01	0.92	297.29	305.19
Sim_91	0.04	-0.01	0.69	297.13	305.04
Sim_92	0.11	0.00	0.28	296.08	303.98
Sim_93	0.14	0.01	0.15	295.15	303.05
Sim_94	-0.04	-0.01	0.69	297.13	305.03
Sim_95	0.11	0.00	0.25	295.93	303.84
Sim_96	0.15	0.01	0.13	294.92	302.82
Sim_97	0.08	0.00	0.42	296.64	304.55
Sim_98	-0.01	-0.01	0.95	297.29	305.20
Sim_99	0.02	-0.01	0.83	297.25	305.15
Sim_100	-0.11	0.00	0.27	296.03	303.94

Tonini (OLS - species level)

Simulation	Latitude	R2	p-value	AIC	BIC
Sim_1	-0.12	0.00	0.25	264.75	272.31
Sim_2	0.08	0.00	0.43	265.44	273.00
Sim_3	-0.10	0.00	0.33	265.12	272.68
Sim_4	-0.02	-0.01	0.85	266.04	273.61
Sim_5	0.06	-0.01	0.56	265.73	273.30
Sim_6	-0.01	-0.01	0.94	266.07	273.64
Sim_7	0.19	0.03	0.07	262.62	270.19
Sim_8	0.18	0.02	0.08	262.98	270.54
Sim_9	0.13	0.01	0.20	264.42	271.99
Sim_10	-0.12	0.00	0.27	264.82	272.38
Sim_11	-0.01	-0.01	0.93	266.07	273.64
Sim_12	0.17	0.02	0.10	263.26	270.83
Sim_13	-0.03	-0.01	0.77	265.99	273.56
Sim_14	-0.05	-0.01	0.64	265.86	273.42
Sim_15	0.05	-0.01	0.66	265.88	273.45

Sim_16	-0.10	0.00	0.33	265.11	272.68
Sim_17	0.02	-0.01	0.88	266.05	273.62
Sim_18	0.01	-0.01	0.92	266.07	273.63
Sim_19	0.16	0.01	0.13	263.70	271.26
Sim_20	0.01	-0.01	0.93	266.07	273.64
Sim_21	-0.02	-0.01	0.82	266.02	273.59
Sim_22	0.04	-0.01	0.73	265.95	273.52
Sim_23	0.01	-0.01	0.94	266.07	273.64
Sim_24	-0.09	0.00	0.40	265.36	272.92
Sim_25	0.01	-0.01	0.90	266.06	273.63
Sim_26	0.06	-0.01	0.56	265.73	273.30
Sim_27	-0.09	0.00	0.38	265.29	272.86
Sim_28	0.07	-0.01	0.52	265.65	273.21
Sim_29	-0.06	-0.01	0.60	265.80	273.36
Sim_30	0.11	0.00	0.30	264.97	272.54
Sim_31	0.00	-0.01	0.99	266.08	273.64
Sim_32	-0.04	-0.01	0.71	265.94	273.51
Sim_33	-0.12	0.00	0.24	264.63	272.20
Sim_34	0.02	-0.01	0.86	266.05	273.61
Sim_35	0.02	-0.01	0.86	266.05	273.61
Sim_36	-0.03	-0.01	0.79	266.01	273.57
Sim_37	-0.08	0.00	0.46	265.52	273.08
Sim_38	0.03	-0.01	0.81	266.02	273.58
Sim_39	0.13	0.01	0.20	264.39	271.95
Sim_40	-0.01	-0.01	0.93	266.07	273.64
Sim_41	-0.17	0.02	0.11	263.46	271.03
Sim_42	0.22	0.04	0.04	261.66	269.23
Sim_43	-0.07	-0.01	0.50	265.60	273.17
Sim_44	0.12	0.00	0.27	264.81	272.38
Sim_45	-0.16	0.01	0.14	263.82	271.39
Sim_46	0.19	0.02	0.08	262.87	270.44
Sim_47	0.00	-0.01	0.97	266.08	273.64
Sim_48	-0.06	-0.01	0.60	265.80	273.36
Sim_49	-0.24	0.05	0.02	260.65	268.22
Sim_50	0.13	0.00	0.23	264.60	272.17
Sim_51	-0.09	0.00	0.41	265.38	272.95
Sim_52	0.01	-0.01	0.95	266.08	273.64
Sim_53	0.09	0.00	0.40	265.34	272.90
Sim_54	0.07	-0.01	0.52	265.66	273.23
Sim_55	-0.04	-0.01	0.74	265.96	273.53
Sim_56	0.10	0.00	0.32	265.08	272.65

Sim_57	-0.23	0.04	0.03	261.07	268.64
Sim_58	0.02	-0.01	0.84	266.04	273.60
Sim_59	0.01	-0.01	0.93	266.07	273.64
Sim_60	0.05	-0.01	0.63	265.84	273.41
Sim_61	0.17	0.02	0.11	263.52	271.09
Sim_62	0.09	0.00	0.37	265.26	272.82
Sim_63	-0.03	-0.01	0.74	265.97	273.53
Sim_64	0.11	0.00	0.29	264.94	272.50
Sim_65	0.16	0.02	0.12	263.56	271.13
Sim_66	0.14	0.01	0.18	264.23	271.79
Sim_67	0.08	0.00	0.42	265.42	272.99
Sim_68	-0.13	0.01	0.23	264.57	272.14
Sim_69	-0.07	-0.01	0.52	265.65	273.21
Sim_70	-0.07	-0.01	0.51	265.63	273.20
Sim_71	-0.16	0.01	0.13	263.67	271.24
Sim_72	0.09	0.00	0.39	265.33	272.90
Sim_73	0.02	-0.01	0.88	266.06	273.62
Sim_74	-0.12	0.00	0.27	264.82	272.39
Sim_75	-0.05	-0.01	0.66	265.88	273.44
Sim_76	-0.03	-0.01	0.76	265.98	273.55
Sim_77	0.06	-0.01	0.60	265.80	273.36
Sim_78	-0.01	-0.01	0.91	266.07	273.63
Sim_79	0.00	-0.01	0.97	266.08	273.64
Sim_80	-0.23	0.04	0.03	261.03	268.59
Sim_81	-0.16	0.01	0.14	263.79	271.35
Sim_82	-0.04	-0.01	0.71	265.94	273.50
Sim_83	0.07	-0.01	0.53	265.67	273.24
Sim_84	0.07	-0.01	0.52	265.66	273.23
Sim_85	-0.03	-0.01	0.75	265.98	273.54
Sim_86	-0.13	0.00	0.23	264.61	272.18
Sim_87	-0.03	-0.01	0.79	266.01	273.57
Sim_88	0.02	-0.01	0.84	266.04	273.60
Sim_89	0.06	-0.01	0.55	265.70	273.27
Sim_90	-0.02	-0.01	0.85	266.04	273.61
Sim_91	-0.03	-0.01	0.78	266.00	273.57
Sim_92	0.19	0.03	0.07	262.70	270.26
Sim_93	0.15	0.01	0.17	264.11	271.68
Sim_94	-0.02	-0.01	0.87	266.05	273.62
Sim_95	0.02	-0.01	0.86	266.05	273.61
Sim_96	0.09	0.00	0.39	265.32	272.88
Sim_97	0.06	-0.01	0.57	265.75	273.32

Sim_98	-0.02	-0.01	0.83	266.03	273.60
Sim_99	0.01	-0.01	0.89	266.06	273.63
Sim_100	-0.04	-0.01	0.74	265.96	273.53

Leache (OLS - species level)

Simulation	Latitude	R2	p-value	AIC	BIC
Sim_1	-0.07	-0.01	0.55	234.50	241.68
Sim_2	0.06	-0.01	0.60	234.57	241.75
Sim_3	-0.11	0.00	0.32	233.85	241.03
Sim_4	-0.10	0.00	0.36	233.98	241.17
Sim_5	0.04	-0.01	0.72	234.73	241.91
Sim_6	0.01	-0.01	0.95	234.86	242.04
Sim_7	0.19	0.03	0.08	231.75	238.93
Sim_8	0.14	0.01	0.20	233.17	240.35
Sim_9	0.09	0.00	0.40	234.14	241.32
Sim_10	-0.11	0.00	0.33	233.87	241.06
Sim_11	0.08	-0.01	0.48	234.34	241.52
Sim_12	0.17	0.02	0.12	232.35	239.53
Sim_13	0.02	-0.01	0.85	234.82	242.01
Sim_14	-0.10	0.00	0.36	233.99	241.17
Sim_15	0.10	0.00	0.38	234.06	241.24
Sim_16	-0.02	-0.01	0.87	234.83	242.02
Sim_17	0.05	-0.01	0.64	234.64	241.82
Sim_18	-0.01	-0.01	0.95	234.86	242.04
Sim_19	0.20	0.03	0.07	231.49	238.67
Sim_20	0.04	-0.01	0.70	234.71	241.89
Sim_21	0.03	-0.01	0.78	234.78	241.97
Sim_22	0.03	-0.01	0.78	234.78	241.96
Sim_23	0.00	-0.01	0.98	234.86	242.04
Sim_24	-0.03	-0.01	0.77	234.78	241.96
Sim_25	0.05	-0.01	0.67	234.67	241.85
Sim_26	0.10	0.00	0.39	234.10	241.28
Sim_27	-0.10	0.00	0.36	233.99	241.17
Sim_28	0.08	-0.01	0.47	234.32	241.51
Sim_29	-0.11	0.00	0.33	233.88	241.07
Sim_30	0.08	-0.01	0.49	234.37	241.55
Sim_31	0.03	-0.01	0.77	234.77	241.96
Sim_32	-0.03	-0.01	0.76	234.77	241.95
Sim_33	-0.10	0.00	0.35	233.98	241.16
Sim_34	0.05	-0.01	0.64	234.63	241.82

Sim_35	0.06	-0.01	0.58	234.55	241.73
Sim_36	0.01	-0.01	0.92	234.85	242.03
Sim_37	-0.07	-0.01	0.54	234.48	241.66
Sim_38	0.01	-0.01	0.96	234.86	242.04
Sim_39	0.17	0.02	0.12	232.39	239.57
Sim_40	-0.07	-0.01	0.53	234.45	241.63
Sim_41	-0.19	0.02	0.10	232.03	239.22
Sim_42	0.26	0.06	0.02	229.20	236.38
Sim_43	-0.08	-0.01	0.46	234.29	241.48
Sim_44	0.12	0.00	0.28	233.64	240.82
Sim_45	-0.18	0.02	0.11	232.18	239.36
Sim_46	0.14	0.01	0.21	233.27	240.46
Sim_47	0.02	-0.01	0.88	234.84	242.02
Sim_48	-0.03	-0.01	0.77	234.77	241.96
Sim_49	-0.22	0.04	0.05	230.95	238.13
Sim_50	0.10	0.00	0.38	234.08	241.27
Sim_51	-0.17	0.02	0.14	232.58	239.76
Sim_52	-0.01	-0.01	0.93	234.85	242.04
Sim_53	0.05	-0.01	0.67	234.68	241.86
Sim_54	0.09	0.00	0.42	234.18	241.37
Sim_55	-0.07	-0.01	0.53	234.46	241.64
Sim_56	0.04	-0.01	0.70	234.70	241.89
Sim_57	-0.27	0.06	0.02	228.84	236.02
Sim_58	0.00	-0.01	0.97	234.86	242.04
Sim_59	-0.02	-0.01	0.89	234.84	242.02
Sim_60	0.01	-0.01	0.92	234.85	242.03
Sim_61	0.15	0.01	0.18	233.02	240.20
Sim_62	0.09	0.00	0.41	234.17	241.36
Sim_63	-0.06	-0.01	0.59	234.56	241.75
Sim_64	0.05	-0.01	0.65	234.65	241.83
Sim_65	0.21	0.03	0.06	231.11	238.29
Sim_66	0.14	0.01	0.23	233.35	240.53
Sim_67	0.01	-0.01	0.95	234.86	242.04
Sim_68	-0.14	0.01	0.21	233.23	240.41
Sim_69	-0.07	-0.01	0.55	234.50	241.68
Sim_70	-0.02	-0.01	0.89	234.84	242.03
Sim_71	-0.14	0.01	0.20	233.16	240.35
Sim_72	0.08	-0.01	0.49	234.36	241.54
Sim_73	0.06	-0.01	0.59	234.57	241.75
Sim_74	-0.03	-0.01	0.81	234.80	241.99
Sim_75	-0.05	-0.01	0.69	234.69	241.88

Sim_76	-0.05	-0.01	0.68	234.69	241.87
Sim_77	0.02	-0.01	0.87	234.83	242.02
Sim_78	-0.01	-0.01	0.93	234.85	242.04
Sim_79	0.01	-0.01	0.95	234.86	242.04
Sim_80	-0.21	0.03	0.07	231.37	238.55
Sim_81	-0.08	-0.01	0.48	234.35	241.53
Sim_82	-0.03	-0.01	0.81	234.80	241.99
Sim_83	0.03	-0.01	0.82	234.81	241.99
Sim_84	0.01	-0.01	0.93	234.85	242.04
Sim_85	0.01	-0.01	0.91	234.85	242.03
Sim_86	-0.16	0.01	0.17	232.88	240.06
Sim_87	-0.07	-0.01	0.51	234.42	241.60
Sim_88	-0.02	-0.01	0.88	234.84	242.02
Sim_89	0.10	0.00	0.40	234.12	241.30
Sim_90	0.01	-0.01	0.96	234.86	242.04
Sim_91	0.03	-0.01	0.81	234.80	241.98
Sim_92	0.23	0.04	0.04	230.62	237.80
Sim_93	0.18	0.02	0.11	232.28	239.46
Sim_94	0.01	-0.01	0.96	234.86	242.04
Sim_95	-0.05	-0.01	0.69	234.70	241.88
Sim_96	0.10	0.00	0.37	234.03	241.21
Sim_97	0.12	0.00	0.27	233.62	240.81
Sim_98	-0.03	-0.01	0.80	234.79	241.98
Sim_99	0.04	-0.01	0.74	234.75	241.93
Sim_100	-0.04	-0.01	0.71	234.72	241.90

Tonini (PGLS)

Simulation	Latitude	R2	p-value
Sim_1	-0.12	0.00	0.25
Sim_2	0.08	0.00	0.43
Sim_3	-0.10	0.00	0.33
Sim_4	-0.02	-0.01	0.85
Sim_5	0.06	-0.01	0.56
Sim_6	-0.01	-0.01	0.94
Sim_7	0.19	0.03	0.07
Sim_8	0.18	0.02	0.08
Sim_9	0.13	0.01	0.20
Sim_10	-0.12	0.00	0.27
Sim_11	-0.01	-0.01	0.93
Sim_12	0.17	0.02	0.10

Sim_13	-0.03	-0.01	0.77
Sim_14	-0.05	-0.01	0.64
Sim_15	0.05	-0.01	0.66
Sim_16	-0.10	0.00	0.33
Sim_17	0.02	-0.01	0.88
Sim_18	0.01	-0.01	0.92
Sim_19	0.16	0.01	0.13
Sim_20	0.01	-0.01	0.93
Sim_21	-0.02	-0.01	0.82
Sim_22	0.04	-0.01	0.73
Sim_23	0.01	-0.01	0.94
Sim_24	-0.09	0.00	0.40
Sim_25	0.01	-0.01	0.90
Sim_26	0.06	-0.01	0.56
Sim_27	-0.09	0.00	0.38
Sim_28	0.07	-0.01	0.52
Sim_29	-0.06	-0.01	0.60
Sim_30	0.11	0.00	0.30
Sim_31	0.00	-0.01	0.99
Sim_32	-0.04	-0.01	0.71
Sim_33	-0.12	0.00	0.24
Sim_34	0.02	-0.01	0.86
Sim_35	0.02	-0.01	0.86
Sim_36	-0.03	-0.01	0.79
Sim_37	-0.08	0.00	0.46
Sim_38	0.03	-0.01	0.81
Sim_39	0.13	0.01	0.20
Sim_40	-0.01	-0.01	0.93
Sim_41	-0.17	0.02	0.11
Sim_42	0.22	0.04	0.04
Sim_43	-0.07	-0.01	0.50
Sim_44	0.12	0.00	0.27
Sim_45	-0.16	0.01	0.14
Sim_46	0.19	0.02	0.08
Sim_47	0.00	-0.01	0.97
Sim_48	-0.06	-0.01	0.60
Sim_49	-0.24	0.05	0.02
Sim_50	0.13	0.00	0.23
Sim_51	-0.09	0.00	0.41
Sim_52	0.01	-0.01	0.95
Sim_53	0.09	0.00	0.40

Sim_54	0.07	-0.01	0.52
Sim_55	-0.04	-0.01	0.74
Sim_56	0.10	0.00	0.32
Sim_57	-0.23	0.04	0.03
Sim_58	0.02	-0.01	0.84
Sim_59	0.01	-0.01	0.93
Sim_60	0.05	-0.01	0.63
Sim_61	0.17	0.02	0.11
Sim_62	0.09	0.00	0.37
Sim_63	-0.03	-0.01	0.74
Sim_64	0.11	0.00	0.29
Sim_65	0.16	0.02	0.12
Sim_66	0.14	0.01	0.18
Sim_67	0.08	0.00	0.42
Sim_68	-0.13	0.01	0.23
Sim_69	-0.07	-0.01	0.52
Sim_70	-0.07	-0.01	0.51
Sim_71	-0.16	0.01	0.13
Sim_72	0.09	0.00	0.39
Sim_73	0.02	-0.01	0.88
Sim_74	-0.12	0.00	0.27
Sim_75	-0.05	-0.01	0.66
Sim_76	-0.03	-0.01	0.76
Sim_77	0.06	-0.01	0.60
Sim_78	-0.01	-0.01	0.91
Sim_79	0.00	-0.01	0.97
Sim_80	-0.23	0.04	0.03
Sim_81	-0.16	0.01	0.14
Sim_82	-0.04	-0.01	0.71
Sim_83	0.07	-0.01	0.53
Sim_84	0.07	-0.01	0.52
Sim_85	-0.03	-0.01	0.75
Sim_86	-0.13	0.00	0.23
Sim_87	-0.03	-0.01	0.79
Sim_88	0.02	-0.01	0.84
Sim_89	0.06	-0.01	0.55
Sim_90	-0.02	-0.01	0.85
Sim_91	-0.03	-0.01	0.78
Sim_92	0.19	0.03	0.07
Sim_93	0.15	0.01	0.17
Sim_94	-0.02	-0.01	0.87

Sim_95	0.02	-0.01	0.86
Sim_96	0.09	0.00	0.39
Sim_97	0.06	-0.01	0.57
Sim_98	-0.02	-0.01	0.83
Sim_99	0.01	-0.01	0.89
Sim_100	-0.04	-0.01	0.74

Leache (PGLS)

Simulation	Latitude	R2	p-value
Sim_1	-0.09	-0.01	0.44
Sim_2	0.08	-0.01	0.51
Sim_3	-0.12	0.00	0.29
Sim_4	-0.08	-0.01	0.47
Sim_5	0.02	-0.01	0.85
Sim_6	0.02	-0.01	0.90
Sim_7	0.19	0.02	0.11
Sim_8	0.15	0.01	0.19
Sim_9	0.12	0.00	0.29
Sim_10	-0.10	0.00	0.42
Sim_11	0.06	-0.01	0.63
Sim_12	0.19	0.02	0.10
Sim_13	0.03	-0.01	0.82
Sim_14	-0.09	-0.01	0.45
Sim_15	0.08	-0.01	0.51
Sim_16	-0.04	-0.01	0.72
Sim_17	0.06	-0.01	0.58
Sim_18	-0.04	-0.01	0.76
Sim_19	0.19	0.02	0.11
Sim_20	0.02	-0.01	0.83
Sim_21	0.03	-0.01	0.81
Sim_22	0.02	-0.01	0.88
Sim_23	0.00	-0.01	0.98
Sim_24	-0.04	-0.01	0.71
Sim_25	0.06	-0.01	0.58
Sim_26	0.10	0.00	0.38
Sim_27	-0.13	0.00	0.26
Sim_28	0.09	-0.01	0.44
Sim_29	-0.09	0.00	0.43
Sim_30	0.08	-0.01	0.47
Sim_31	0.04	-0.01	0.75

Sim_32	-0.02	-0.01	0.83
Sim_33	-0.13	0.00	0.25
Sim_34	0.06	-0.01	0.60
Sim_35	0.05	-0.01	0.69
Sim_36	0.01	-0.01	0.95
Sim_37	-0.06	-0.01	0.58
Sim_38	-0.01	-0.01	0.94
Sim_39	0.19	0.02	0.10
Sim_40	-0.06	-0.01	0.60
Sim_41	-0.18	0.02	0.12
Sim_42	0.26	0.05	0.02
Sim_43	-0.10	0.00	0.40
Sim_44	0.12	0.00	0.29
Sim_45	-0.19	0.02	0.10
Sim_46	0.17	0.01	0.15
Sim_47	0.02	-0.01	0.83
Sim_48	-0.06	-0.01	0.58
Sim_49	-0.23	0.04	0.05
Sim_50	0.08	-0.01	0.51
Sim_51	-0.16	0.01	0.16
Sim_52	0.00	-0.01	0.98
Sim_53	0.06	-0.01	0.59
Sim_54	0.09	-0.01	0.45
Sim_55	-0.05	-0.01	0.64
Sim_56	0.04	-0.01	0.70
Sim_57	-0.27	0.06	0.02
Sim_58	-0.01	-0.01	0.91
Sim_59	0.01	-0.01	0.96
Sim_60	0.03	-0.01	0.82
Sim_61	0.14	0.01	0.22
Sim_62	0.09	0.00	0.42
Sim_63	-0.07	-0.01	0.55
Sim_64	0.06	-0.01	0.62
Sim_65	0.22	0.03	0.06
Sim_66	0.14	0.01	0.24
Sim_67	0.02	-0.01	0.84
Sim_68	-0.15	0.01	0.20
Sim_69	-0.06	-0.01	0.60
Sim_70	-0.03	-0.01	0.77
Sim_71	-0.16	0.01	0.16
Sim_72	0.08	-0.01	0.49

Sim_73	0.05	-0.01	0.64
Sim_74	-0.05	-0.01	0.70
Sim_75	-0.06	-0.01	0.63
Sim_76	-0.04	-0.01	0.73
Sim_77	0.00	-0.01	0.97
Sim_78	-0.01	-0.01	0.94
Sim_79	0.04	-0.01	0.70
Sim_80	-0.20	0.03	0.07
Sim_81	-0.14	0.00	0.26
Sim_82	-0.02	-0.01	0.89
Sim_83	0.01	-0.01	0.92
Sim_84	-0.01	-0.01	0.91
Sim_85	0.01	-0.01	0.92
Sim_86	-0.17	0.01	0.15
Sim_87	-0.05	-0.01	0.64
Sim_88	-0.04	-0.01	0.74
Sim_89	0.09	-0.01	0.45
Sim_90	0.02	-0.01	0.87
Sim_91	0.01	-0.01	0.96
Sim_92	0.24	0.04	0.04
Sim_93	0.16	0.01	0.16
Sim_94	0.00	-0.01	0.98
Sim_95	-0.05	-0.01	0.67
Sim_96	0.09	0.00	0.43
Sim_97	0.10	0.00	0.38
Sim_98	-0.03	-0.01	0.81
Sim_99	0.02	-0.01	0.86
Sim_100	-0.04	-0.01	0.72

All species (OLS – sites level)

Simulation	Latitude	R2	p-value	AIC	BIC
Sim_1	-0.07	0.00	0.00	7799.37	7817.13
Sim_2	0.15	0.02	0.00	7833.73	7851.52
Sim_3	-0.24	0.06	0.00	7797.91	7815.73
Sim_4	0.14	0.02	0.00	7911.48	7929.30
Sim_5	0.18	0.03	0.00	7833.49	7851.29
Sim_6	-0.06	0.00	0.00	7861.12	7878.90
Sim_7	0.54	0.29	0.00	7003.50	7021.31
Sim_8	0.25	0.06	0.00	7684.54	7702.32
Sim_9	-0.23	0.05	0.00	7783.50	7801.31

Sim_10	-0.20	0.04	0.00	7708.92	7726.68
Sim_11	0.12	0.01	0.00	7831.33	7849.12
Sim_12	-0.18	0.03	0.00	7779.80	7797.58
Sim_13	0.19	0.03	0.00	7909.94	7927.77
Sim_14	0.23	0.05	0.00	7718.38	7736.16
Sim_15	0.19	0.04	0.00	7844.84	7862.65
Sim_16	-0.05	0.00	0.01	7947.82	7965.63
Sim_17	0.50	0.25	0.00	7109.47	7127.27
Sim_18	-0.35	0.12	0.00	7587.22	7605.03
Sim_19	-0.14	0.02	0.00	7602.75	7620.45
Sim_20	0.20	0.04	0.00	7870.55	7888.37
Sim_21	0.04	0.00	0.03	7946.52	7964.33
Sim_22	0.13	0.02	0.00	7887.30	7905.11
Sim_23	0.09	0.01	0.00	7947.09	7964.91
Sim_24	0.02	0.00	0.30	7995.39	8013.21
Sim_25	0.08	0.01	0.00	7954.20	7972.02
Sim_26	0.10	0.01	0.00	7873.31	7891.10
Sim_27	-0.35	0.12	0.00	7552.65	7570.45
Sim_28	0.08	0.01	0.00	7901.69	7919.49
Sim_29	0.13	0.02	0.00	7970.69	7988.53
Sim_30	0.29	0.08	0.00	7689.08	7706.89
Sim_31	0.33	0.11	0.00	7691.78	7709.61
Sim_32	-0.40	0.16	0.00	7411.67	7429.46
Sim_33	0.29	0.09	0.00	7707.76	7725.57
Sim_34	0.62	0.38	0.00	6396.95	6414.67
Sim_35	0.07	0.01	0.00	7700.21	7717.93
Sim_36	0.36	0.13	0.00	7454.94	7472.71
Sim_37	-0.13	0.02	0.00	7837.57	7855.36
Sim_38	0.29	0.08	0.00	7764.20	7782.04
Sim_39	0.61	0.38	0.00	6683.10	6700.93
Sim_40	-0.25	0.06	0.00	7741.40	7759.21
Sim_41	0.33	0.11	0.00	7616.40	7634.20
Sim_42	0.00	0.00	0.89	8019.14	8036.98
Sim_43	0.02	0.00	0.33	7913.23	7931.02
Sim_44	0.52	0.27	0.00	7134.56	7152.40
Sim_45	-0.41	0.17	0.00	7163.57	7181.27
Sim_46	0.49	0.24	0.00	7168.18	7185.99
Sim_47	-0.19	0.04	0.00	7843.74	7861.55
Sim_48	0.19	0.04	0.00	7909.90	7927.74
Sim_49	-0.20	0.04	0.00	7780.01	7797.80
Sim_50	-0.23	0.05	0.00	7793.43	7811.24

Sim_51	-0.35	0.12	0.00	7612.70	7630.52
Sim_52	-0.15	0.02	0.00	7939.46	7957.30
Sim_53	0.45	0.21	0.00	7286.31	7304.11
Sim_54	0.27	0.08	0.00	7535.52	7553.26
Sim_55	0.56	0.31	0.00	6874.98	6892.77
Sim_56	0.62	0.38	0.00	6466.76	6484.50
Sim_57	-0.46	0.21	0.00	7293.16	7310.98
Sim_58	-0.40	0.16	0.00	7506.67	7524.50
Sim_59	-0.08	0.01	0.00	7984.76	8002.60
Sim_60	0.06	0.00	0.00	7869.19	7886.97
Sim_61	0.03	0.00	0.11	7942.84	7960.65
Sim_62	0.56	0.32	0.00	6806.93	6824.71
Sim_63	0.29	0.09	0.00	7680.19	7698.00
Sim_64	-0.21	0.04	0.00	7872.15	7889.98
Sim_65	0.52	0.27	0.00	6782.21	6799.90
Sim_66	-0.02	0.00	0.42	7953.23	7971.05
Sim_67	-0.20	0.04	0.00	7791.43	7809.22
Sim_68	0.21	0.04	0.00	7836.31	7854.13
Sim_69	-0.07	0.00	0.00	7952.23	7970.04
Sim_70	0.00	0.00	0.84	7865.88	7883.66
Sim_71	-0.27	0.07	0.00	7641.59	7659.36
Sim_72	0.24	0.06	0.00	7799.95	7817.77
Sim_73	0.46	0.21	0.00	7293.50	7311.31
Sim_74	0.32	0.10	0.00	7653.16	7670.97
Sim_75	0.05	0.00	0.01	7957.66	7975.48
Sim_76	-0.45	0.20	0.00	7192.47	7210.24
Sim_77	0.25	0.06	0.00	7784.81	7802.62
Sim_78	0.17	0.03	0.00	7888.04	7905.86
Sim_79	-0.14	0.02	0.00	7850.53	7868.32
Sim_80	-0.10	0.01	0.00	7823.07	7840.84
Sim_81	0.38	0.14	0.00	7463.07	7480.86
Sim_82	0.45	0.20	0.00	7338.18	7355.99
Sim_83	0.17	0.03	0.00	7866.27	7884.08
Sim_84	-0.14	0.02	0.00	7927.03	7944.85
Sim_85	0.03	0.00	0.12	7903.21	7921.01
Sim_86	-0.36	0.13	0.00	7606.81	7624.64
Sim_87	0.19	0.04	0.00	7899.80	7917.63
Sim_88	-0.13	0.02	0.00	7790.37	7808.14
Sim_89	0.31	0.10	0.00	7645.37	7663.17
Sim_90	0.20	0.04	0.00	7720.41	7738.18
Sim_91	0.22	0.05	0.00	7726.02	7743.80

Sim_92	0.13	0.02	0.00	7943.33	7961.15
Sim_93	0.17	0.03	0.00	7892.10	7909.92
Sim_94	-0.25	0.06	0.00	7738.69	7756.49
Sim_95	-0.29	0.09	0.00	7722.73	7740.55
Sim_96	0.04	0.00	0.03	7937.88	7955.69
Sim_97	0.16	0.03	0.00	7936.66	7954.50
Sim_98	0.45	0.20	0.00	7216.57	7234.35
Sim_99	-0.06	0.00	0.00	7931.77	7949.58
Sim_100	0.25	0.06	0.00	7738.98	7756.78

Tonini (OLS – sites level)

Simulation	Latitude	R2	p-value	AIC	BIC
Sim_1	-0.07	0.01	0.00	7793.76	7811.52
Sim_2	0.16	0.02	0.00	7829.09	7846.88
Sim_3	-0.24	0.06	0.00	7797.78	7815.59
Sim_4	0.17	0.03	0.00	7880.03	7897.85
Sim_5	0.19	0.04	0.00	7824.19	7841.99
Sim_6	-0.05	0.00	0.01	7864.61	7882.39
Sim_7	0.53	0.28	0.00	7022.98	7040.79
Sim_8	0.25	0.06	0.00	7676.95	7694.72
Sim_9	-0.21	0.04	0.00	7807.37	7825.17
Sim_10	-0.16	0.03	0.00	7744.11	7761.87
Sim_11	0.12	0.01	0.00	7820.11	7837.88
Sim_12	-0.19	0.03	0.00	7774.55	7792.33
Sim_13	0.20	0.04	0.00	7894.39	7912.23
Sim_14	0.26	0.07	0.00	7673.38	7691.17
Sim_15	0.18	0.03	0.00	7861.75	7879.56
Sim_16	-0.06	0.00	0.00	7942.87	7960.68
Sim_17	0.50	0.25	0.00	7110.28	7128.08
Sim_18	-0.35	0.12	0.00	7587.89	7605.71
Sim_19	-0.19	0.03	0.00	7562.80	7580.50
Sim_20	0.15	0.02	0.00	7922.53	7940.35
Sim_21	0.03	0.00	0.09	7948.26	7966.08
Sim_22	0.13	0.02	0.00	7888.09	7905.89
Sim_23	0.07	0.00	0.00	7928.48	7946.29
Sim_24	-0.03	0.00	0.15	7994.35	8012.18
Sim_25	0.07	0.01	0.00	7955.52	7973.34
Sim_26	0.10	0.01	0.00	7870.88	7888.67
Sim_27	-0.36	0.13	0.00	7515.96	7533.76
Sim_28	0.12	0.02	0.00	7876.23	7894.03

Sim_29	0.15	0.02	0.00	7959.41	7977.25
Sim_30	0.30	0.09	0.00	7666.30	7684.11
Sim_31	0.33	0.11	0.00	7699.88	7717.72
Sim_32	-0.39	0.15	0.00	7418.27	7436.06
Sim_33	0.28	0.08	0.00	7727.00	7744.82
Sim_34	0.62	0.38	0.00	6385.70	6403.41
Sim_35	-0.01	0.00	0.63	7684.06	7701.77
Sim_36	0.34	0.11	0.00	7492.34	7510.10
Sim_37	-0.09	0.01	0.00	7799.80	7817.56
Sim_38	0.29	0.08	0.00	7736.05	7753.88
Sim_39	0.62	0.39	0.00	6603.77	6621.60
Sim_40	-0.20	0.04	0.00	7815.70	7833.51
Sim_41	0.34	0.11	0.00	7600.97	7618.78
Sim_42	-0.06	0.00	0.00	8009.51	8027.34
Sim_43	0.07	0.00	0.00	7900.80	7918.60
Sim_44	0.52	0.27	0.00	7104.39	7122.23
Sim_45	-0.36	0.13	0.00	7243.00	7260.68
Sim_46	0.52	0.27	0.00	7039.78	7057.59
Sim_47	-0.17	0.03	0.00	7858.84	7876.65
Sim_48	0.16	0.02	0.00	7947.55	7965.38
Sim_49	-0.20	0.04	0.00	7776.38	7794.17
Sim_50	-0.31	0.10	0.00	7661.93	7679.74
Sim_51	-0.34	0.11	0.00	7633.39	7651.21
Sim_52	-0.16	0.02	0.00	7938.40	7956.24
Sim_53	0.49	0.24	0.00	7166.43	7184.23
Sim_54	0.28	0.08	0.00	7520.00	7537.73
Sim_55	0.59	0.35	0.00	6703.07	6720.87
Sim_56	0.60	0.36	0.00	6534.77	6552.51
Sim_57	-0.43	0.18	0.00	7391.20	7409.02
Sim_58	-0.40	0.16	0.00	7503.31	7521.15
Sim_59	-0.05	0.00	0.01	7997.56	8015.40
Sim_60	0.15	0.02	0.00	7774.84	7792.61
Sim_61	0.03	0.00	0.17	7943.48	7961.29
Sim_62	0.55	0.30	0.00	6886.23	6904.01
Sim_63	0.24	0.06	0.00	7763.83	7781.64
Sim_64	-0.15	0.02	0.00	7937.47	7955.30
Sim_65	0.50	0.25	0.00	6745.77	6763.41
Sim_66	-0.08	0.01	0.00	7933.96	7951.78
Sim_67	-0.19	0.04	0.00	7800.43	7818.22
Sim_68	0.19	0.04	0.00	7854.47	7872.29
Sim_69	-0.06	0.00	0.00	7955.12	7972.94

Sim_70	-0.02	0.00	0.30	7864.86	7882.64
Sim_71	-0.26	0.06	0.00	7659.62	7677.39
Sim_72	0.26	0.07	0.00	7769.70	7787.52
Sim_73	0.44	0.19	0.00	7353.64	7371.45
Sim_74	0.31	0.10	0.00	7672.42	7690.24
Sim_75	0.04	0.00	0.05	7961.22	7979.04
Sim_76	-0.46	0.22	0.00	7082.17	7099.91
Sim_77	0.26	0.07	0.00	7760.29	7778.11
Sim_78	0.17	0.03	0.00	7884.32	7902.14
Sim_79	-0.17	0.03	0.00	7742.95	7760.72
Sim_80	-0.08	0.01	0.00	7821.69	7839.46
Sim_81	0.33	0.11	0.00	7570.64	7588.44
Sim_82	0.45	0.20	0.00	7340.14	7357.96
Sim_83	0.20	0.04	0.00	7834.05	7851.86
Sim_84	-0.19	0.04	0.00	7868.69	7886.51
Sim_85	0.06	0.00	0.00	7894.48	7912.28
Sim_86	-0.34	0.12	0.00	7648.38	7666.22
Sim_87	0.22	0.05	0.00	7858.56	7876.39
Sim_88	-0.13	0.02	0.00	7793.07	7810.84
Sim_89	0.33	0.11	0.00	7600.93	7618.73
Sim_90	0.24	0.06	0.00	7650.13	7667.89
Sim_91	0.20	0.04	0.00	7734.15	7751.92
Sim_92	0.13	0.02	0.00	7940.67	7958.49
Sim_93	0.18	0.03	0.00	7882.06	7899.88
Sim_94	-0.23	0.05	0.00	7768.44	7786.23
Sim_95	-0.32	0.10	0.00	7677.16	7694.99
Sim_96	-0.05	0.00	0.00	7934.08	7951.89
Sim_97	0.11	0.01	0.00	7978.31	7996.15
Sim_98	0.44	0.19	0.00	7264.72	7282.50
Sim_99	-0.05	0.00	0.01	7935.19	7953.00
Sim_100	0.26	0.07	0.00	7722.02	7739.82

Leache (OLS – sites level)

Simulation	Latitude	R2	p-value	AIC	BIC
Sim_1	-0.07	0.00	0.00	7686.92	7704.63
Sim_2	0.19	0.04	0.00	7753.60	7771.38
Sim_3	-0.20	0.04	0.00	7849.35	7867.17
Sim_4	0.14	0.02	0.00	7911.64	7929.46
Sim_5	0.19	0.04	0.00	7824.25	7842.05
Sim_6	-0.06	0.00	0.00	7661.66	7679.36

Sim_7	0.58	0.34	0.00	6793.64	6811.45
Sim_8	0.08	0.01	0.00	7674.46	7692.17
Sim_9	-0.25	0.06	0.00	7749.71	7767.52
Sim_10	-0.13	0.02	0.00	7757.83	7775.58
Sim_11	0.23	0.05	0.00	7610.07	7627.81
Sim_12	-0.21	0.05	0.00	7674.40	7692.16
Sim_13	0.10	0.01	0.00	7980.69	7998.53
Sim_14	0.28	0.08	0.00	7650.41	7668.20
Sim_15	0.23	0.05	0.00	7798.55	7816.37
Sim_16	-0.04	0.00	0.03	7949.05	7966.86
Sim_17	0.46	0.21	0.00	7263.79	7281.59
Sim_18	-0.35	0.12	0.00	7588.42	7606.23
Sim_19	-0.25	0.06	0.00	7464.43	7482.13
Sim_20	0.21	0.04	0.00	7855.89	7873.71
Sim_21	0.11	0.01	0.00	7916.59	7934.40
Sim_22	0.05	0.00	0.01	7927.96	7945.76
Sim_23	-0.07	0.00	0.00	7862.30	7880.08
Sim_24	-0.07	0.00	0.00	7902.94	7920.73
Sim_25	0.07	0.00	0.00	7957.27	7975.09
Sim_26	0.15	0.02	0.00	7801.24	7819.02
Sim_27	-0.28	0.08	0.00	7629.23	7647.01
Sim_28	0.19	0.04	0.00	7802.32	7820.11
Sim_29	0.19	0.04	0.00	7911.08	7928.92
Sim_30	0.29	0.08	0.00	7631.71	7649.49
Sim_31	0.31	0.10	0.00	7733.10	7750.94
Sim_32	-0.42	0.18	0.00	7347.34	7365.13
Sim_33	0.33	0.11	0.00	7608.48	7626.29
Sim_34	0.57	0.32	0.00	6600.03	6617.72
Sim_35	-0.16	0.02	0.00	7483.00	7500.66
Sim_36	0.32	0.11	0.00	7256.98	7274.64
Sim_37	-0.15	0.02	0.00	7822.72	7840.50
Sim_38	0.22	0.05	0.00	7810.99	7828.80
Sim_39	0.60	0.35	0.00	6713.28	6731.09
Sim_40	-0.11	0.01	0.00	7790.02	7807.78
Sim_41	0.51	0.26	0.00	7077.52	7095.32
Sim_42	-0.10	0.01	0.00	7992.81	8010.64
Sim_43	0.05	0.00	0.01	7904.89	7922.68
Sim_44	0.53	0.28	0.00	7096.44	7114.27
Sim_45	-0.39	0.15	0.00	7096.59	7114.24
Sim_46	0.49	0.24	0.00	7187.86	7205.67
Sim_47	-0.20	0.04	0.00	7833.66	7851.47

Sim_48	0.20	0.04	0.00	7896.25	7914.09
Sim_49	-0.27	0.07	0.00	7681.88	7699.67
Sim_50	-0.33	0.11	0.00	7428.12	7445.85
Sim_51	-0.34	0.11	0.00	7631.87	7649.69
Sim_52	-0.12	0.02	0.00	7926.98	7944.80
Sim_53	0.45	0.20	0.00	7040.79	7058.49
Sim_54	0.27	0.07	0.00	7534.07	7551.80
Sim_55	0.61	0.37	0.00	6624.22	6642.01
Sim_56	0.62	0.38	0.00	6430.69	6448.42
Sim_57	-0.28	0.08	0.00	7725.49	7743.30
Sim_58	-0.39	0.15	0.00	7530.26	7548.09
Sim_59	0.04	0.00	0.04	7994.90	8012.73
Sim_60	0.17	0.03	0.00	7731.32	7749.08
Sim_61	-0.05	0.00	0.01	7936.37	7954.18
Sim_62	0.55	0.30	0.00	6864.07	6881.85
Sim_63	0.27	0.07	0.00	7712.24	7730.04
Sim_64	-0.12	0.01	0.00	7959.84	7977.67
Sim_65	0.51	0.26	0.00	6781.43	6799.10
Sim_66	-0.05	0.00	0.01	7946.10	7963.91
Sim_67	-0.11	0.01	0.00	7828.52	7846.30
Sim_68	0.20	0.04	0.00	7841.60	7859.42
Sim_69	-0.05	0.00	0.01	7957.96	7975.78
Sim_70	-0.09	0.01	0.00	7843.53	7861.31
Sim_71	-0.17	0.03	0.00	7696.31	7714.06
Sim_72	0.26	0.07	0.00	7705.78	7723.57
Sim_73	0.50	0.25	0.00	7129.39	7147.20
Sim_74	0.25	0.06	0.00	7585.75	7603.48
Sim_75	0.05	0.00	0.02	7953.75	7971.57
Sim_76	-0.46	0.21	0.00	7108.42	7126.16
Sim_77	0.19	0.04	0.00	7855.18	7873.00
Sim_78	0.26	0.06	0.00	7778.75	7796.57
Sim_79	-0.25	0.06	0.00	7624.06	7641.81
Sim_80	-0.05	0.00	0.00	7775.63	7793.37
Sim_81	0.40	0.16	0.00	7411.70	7429.49
Sim_82	0.42	0.18	0.00	7409.44	7427.26
Sim_83	0.26	0.07	0.00	7749.84	7767.65
Sim_84	-0.18	0.03	0.00	7888.66	7906.48
Sim_85	0.12	0.02	0.00	7861.98	7879.77
Sim_86	-0.29	0.09	0.00	7665.42	7683.22
Sim_87	0.21	0.05	0.00	7870.67	7888.51
Sim_88	-0.10	0.01	0.00	7812.48	7830.24

Sim_89	0.33	0.11	0.00	7594.73	7612.53
Sim_90	0.14	0.02	0.00	7615.84	7633.55
Sim_91	0.22	0.05	0.00	7654.46	7672.21
Sim_92	0.22	0.05	0.00	7833.87	7851.69
Sim_93	0.08	0.01	0.00	7960.22	7978.04
Sim_94	-0.19	0.04	0.00	7797.26	7815.05
Sim_95	-0.27	0.08	0.00	7753.57	7771.39
Sim_96	-0.09	0.01	0.00	7917.13	7934.94
Sim_97	0.13	0.02	0.00	7959.94	7977.78
Sim_98	0.48	0.23	0.00	7129.54	7147.31
Sim_99	0.02	0.00	0.31	7941.52	7959.33
Sim_100	0.34	0.12	0.00	7578.54	7596.35

All species (SARs)

Simulation	Latitude	R2	AIC.df	AIC	p-value	Intercept	scheme	model
Sim_1	-0.07	0.59	4	5332.91	0	0.01	W	error
Sim_2	-0.2	0.42	4	6307.47	0	0	W	error
Sim_3	0.25	0.32	4	6828.55	0	0	W	error
Sim_4	0.1	0.59	4	5414.35	0	0	W	error
Sim_5	-0.2	0.62	4	5192.23	0	0.01	W	error
Sim_6	0.18	0.45	4	6317.21	0	0	W	error
Sim_7	0.24	0.43	4	6311.45	0	0	W	error
Sim_8	0.19	0.34	4	6784.85	0	0	W	error
Sim_9	-0.05	0.34	4	6801.01	0	0.01	W	error
Sim_10	0.5	0.49	4	6035.41	0	0	W	error
Sim_11	-0.35	0.66	4	4892.74	0	0.01	W	error
Sim_12	-0.15	0.63	4	5009.7	0	0.01	W	error
Sim_13	0.13	0.51	4	5928.81	0	0.02	W	error
Sim_14	0.18	0.53	4	5839.66	0	0.01	W	error
Sim_15	0.04	0.54	4	5777.57	0	0.01	W	error
Sim_16	0.12	0.47	4	6167.63	0	-0.02	S	error
Sim_17	0.08	0.48	4	6121.41	0	0.02	W	error
Sim_18	0.02	0.5	4	6026.81	0	0	W	error
Sim_19	0.07	0.49	4	6081.8	0	0.01	W	error
Sim_20	0.1	0.65	4	4982.02	0	0.02	W	error
Sim_21	-0.35	0.47	4	6121	0	0	W	error
Sim_22	0.1	0.52	4	5900.93	0	0.03	W	error
Sim_23	0.14	0.48	4	6183.15	0	0	W	error
Sim_24	-0.25	0.53	4	5877.1	0	0	W	error
Sim_25	0.28	0.45	4	6243.72	0	0.01	W	error

Sim_26	0.33	0.47	4	6229.55	0	-0.01	W	error
Sim_27	-0.41	0.53	4	5760.51	0	0	W	error
Sim_28	0.3	0.48	4	6117.14	0	0	W	error
Sim_29	0.6	0.72	4	4263.46	0	0.01	W	error
Sim_30	0.09	0.61	4	5140.02	0	0.02	W	error
Sim_31	0.36	0.72	4	4342.72	0	0	W	error
Sim_32	-0.15	0.51	4	5913	0	0	W	error
Sim_33	0.28	0.54	4	5787.71	0	0.01	W	error
Sim_34	0.61	0.66	4	5000.95	0	0	W	error
Sim_35	0.13	0.48	4	6142.06	0	0.01	W	error
Sim_36	-0.26	0.53	4	5849.09	0	0	W	error
Sim_37	0.33	0.49	4	6072.39	0	0.01	W	error
Sim_38	-0.02	0.47	4	6218.15	0	0	W	error
Sim_39	0.03	0.48	4	6070.2	0	0	W	error
Sim_40	0.52	0.5	4	6029.13	0	0.01	W	error
Sim_41	-0.41	0.63	4	4992.21	0	0.01	W	error
Sim_42	0.5	0.53	4	5847.22	0	0	W	error
Sim_43	-0.21	0.56	4	5638.45	0	0	W	error
Sim_44	0.21	0.44	4	6405.85	0	0	W	error
Sim_45	-0.2	0.55	4	5696.57	0	0.01	W	error
Sim_46	0.18	0.42	4	6400.86	0	0.01	W	error
Sim_47	-0.25	0.56	4	5665.86	0	0.01	W	error
Sim_48	-0.35	0.69	4	4719.91	0	0.01	W	error
Sim_49	-0.15	0.4	4	6585.69	0	0.01	W	error
Sim_50	0.46	0.7	4	4560.36	0	0.01	W	error
Sim_51	0.27	0.49	4	5913.39	0	0.01	W	error
Sim_52	0.56	0.62	4	5218.72	0	0	W	error
Sim_53	0.61	0.6	4	5272.42	0	0	W	error
Sim_54	-0.45	0.55	4	5740.72	0	0.01	W	error
Sim_55	-0.39	0.62	4	5244.98	0	0.01	W	error
Sim_56	-0.08	0.46	4	6288.58	0	0.01	W	error
Sim_57	-0.06	0.49	4	5988.62	0	0.01	W	error
Sim_58	0.03	0.57	4	5559.02	0	0.01	W	error
Sim_59	0.03	0.61	4	5333.93	0	0.01	W	error
Sim_60	0.56	0.56	4	5583.46	0	0	W	error
Sim_61	0.3	0.5	4	6008.89	0	0	W	error
Sim_62	-0.22	0.37	4	6709.67	0	0.01	W	error
Sim_63	0.51	0.78	4	3569.12	0	0	W	error
Sim_64	-0.05	0.29	4	7012.76	0	0.03	S	error
Sim_65	-0.19	0.53	4	5781.49	0	0	W	error
Sim_66	0.22	0.48	4	6107.52	0	0.02	W	error

Sim_67	-0.07	0.57	4	5595.83	0	0.01	W	error
Sim_68	0.54	0.59	4	5472.47	0	0	W	error
Sim_69	0.02	0.36	4	6630.14	0	0	W	error
Sim_70	-0.26	0.55	4	5661.47	0	0	W	error
Sim_71	0.24	0.6	4	5364.2	0	0.01	W	error
Sim_72	0.45	0.49	4	6080.69	0	0	W	error
Sim_73	0.33	0.48	4	6112.16	0	0.01	W	error
Sim_74	0.05	0.32	4	6902.88	0	0.01	W	error
Sim_75	-0.46	0.68	4	4679.41	0	0.01	W	error
Sim_76	0.26	0.45	4	6267.3	0	0.02	S	error
Sim_77	0.17	0.55	4	5738.32	0	0	W	error
Sim_78	-0.15	0.68	4	4736.29	0	0	W	error
Sim_79	0.27	0.56	4	5584.81	0	-0.01	W	error
Sim_80	-0.09	0.63	4	5111.99	0	0.01	W	error
Sim_81	0.38	0.43	4	6355.36	0	0.03	S	error
Sim_82	0.45	0.54	4	5783.56	0	0	W	error
Sim_83	0.17	0.23	4	7223.66	0	0	W	error
Sim_84	-0.15	0.54	4	5798.37	0	0	W	error
Sim_85	0.05	0.33	4	6778.69	0	0.01	W	error
Sim_86	-0.38	0.5	4	6037.14	0	0.01	W	error
Sim_87	0.18	0.34	4	6817.19	0	0	W	error
Sim_88	-0.12	0.6	4	5314.92	0	0.02	W	error
Sim_89	0.31	0.58	4	5519.19	0	0	W	error
Sim_90	-0.23	0.26	4	7102.87	0	0	W	error
Sim_91	0.17	0.48	4	6047.86	0	0.01	W	error
Sim_92	0.21	0.54	4	5734.48	0	0.01	W	error
Sim_93	0.13	0.37	4	6670.38	0	0	W	error
Sim_94	0.17	0.51	4	5979.72	0	0.01	W	error
Sim_95	-0.26	0.49	4	6057.82	0	0	W	error
Sim_96	-0.3	0.49	4	6060.17	0	0	W	error
Sim_97	0.04	0.31	4	6894.42	0	0.01	W	error
Sim_98	0.17	0.58	4	5534.64	0	0	W	error
Sim_99	0.46	0.56	4	5614.95	0	0.01	W	error
Sim_100	-0.07	0.49	4	6072.96	0	0.01	W	error

Tonini (SARs)

Simulation	Latitude	R2	AIC	p-value	Intercept	Scheme	Model
sim_1	-0.08	0.57	5519.98	0.00	0.00	W	error
sim_2	-0.16	0.42	6307.33	0.00	0.00	W	error
sim_3	0.26	0.27	7055.52	0.00	0.00	W	error
sim_4	0.10	0.53	5779.59	0.00	0.00	W	error
sim_5	-0.20	0.61	5245.23	0.00	0.01	W	error
sim_6	0.19	0.46	6249.13	0.00	0.00	W	error
sim_7	0.27	0.45	6213.39	0.00	0.01	W	error
sim_8	0.17	0.35	6757.61	0.00	0.00	W	error
sim_9	-0.07	0.35	6749.16	0.00	0.00	W	error
sim_10	0.50	0.50	5991.11	0.00	0.00	W	error
sim_11	-0.35	0.66	4903.92	0.00	0.00	W	error
sim_12	-0.19	0.66	4725.72	0.00	0.01	W	error
sim_13	0.13	0.51	5893.12	0.00	0.01	W	error
sim_14	0.13	0.52	5935.30	0.00	0.00	W	error
sim_15	0.03	0.51	5941.67	0.00	0.01	W	error
sim_16	0.12	0.48	6120.70	0.00	-0.03	S	error
sim_17	0.07	0.35	6726.66	0.00	0.01	W	error
sim_18	-0.02	0.50	6032.60	0.00	0.00	W	error
sim_19	0.07	0.45	6278.79	0.00	0.00	W	error
sim_20	0.10	0.63	5163.29	0.00	0.01	W	error
sim_21	-0.36	0.49	6055.12	0.00	0.00	W	error
sim_22	0.14	0.49	6058.35	0.00	0.03	W	error
sim_23	0.16	0.51	6004.88	0.00	0.00	W	error
sim_24	-0.25	0.56	5667.31	0.00	0.00	W	error
sim_25	0.29	0.49	6079.58	0.00	0.01	W	error
sim_26	0.32	0.46	6281.77	0.00	0.00	W	error
sim_27	-0.41	0.55	5651.46	0.00	0.00	W	error
sim_28	0.29	0.51	5934.79	0.00	0.01	W	error
sim_29	0.61	0.74	4083.66	0.00	0.01	W	error
sim_30	0.00	0.61	5141.03	0.00	0.02	W	error
sim_31	0.32	0.73	4232.57	0.00	0.01	W	error
sim_32	-0.09	0.54	5693.55	0.00	0.00	W	error
sim_33	0.29	0.57	5581.19	0.00	0.01	W	error
sim_34	0.62	0.69	4729.86	0.00	0.00	W	error
sim_35	0.17	0.53	5867.32	0.00	0.01	W	error
sim_36	-0.21	0.52	5853.78	0.00	0.00	W	error
sim_37	0.34	0.50	6013.15	0.00	0.01	W	error

sim_38	-0.07	0.46	6255.39	0.00	0.00	W	error
sim_39	0.08	0.50	6006.95	0.00	0.00	W	error
sim_40	0.52	0.51	6024.64	0.00	0.01	W	error
sim_41	-0.36	0.63	4980.88	0.00	0.01	W	error
sim_42	0.52	0.56	5637.29	0.00	0.00	W	error
sim_43	-0.18	0.54	5760.41	0.00	0.00	W	error
sim_44	0.17	0.48	6176.25	0.00	0.00	W	error
sim_45	-0.20	0.57	5552.51	0.00	0.01	W	error
sim_46	0.19	0.42	6429.91	0.00	0.01	W	error
sim_47	-0.33	0.56	5673.39	0.00	0.01	W	error
sim_48	-0.34	0.70	4614.25	0.00	0.01	W	error
sim_49	-0.15	0.40	6570.31	0.00	0.01	W	error
sim_50	0.49	0.70	4564.89	0.00	0.01	W	error
sim_51	0.28	0.49	5899.88	0.00	0.01	W	error
sim_52	0.59	0.65	4967.94	0.00	0.00	W	error
sim_53	0.59	0.61	5216.18	0.00	0.00	W	error
sim_54	-0.41	0.50	5996.77	0.00	0.01	W	error
sim_55	-0.40	0.56	5679.00	0.00	0.01	W	error
sim_56	-0.04	0.44	6356.03	0.00	0.01	W	error
sim_57	-0.05	0.51	5900.08	0.00	0.01	W	error
sim_58	0.14	0.49	6003.80	0.00	0.02	W	error
sim_59	0.02	0.61	5283.51	0.00	0.01	W	error
sim_60	0.54	0.57	5525.57	0.00	0.00	W	error
sim_61	0.24	0.45	6284.32	0.00	0.00	W	error
sim_62	-0.15	0.42	6463.83	0.00	0.01	W	error
sim_63	0.49	0.80	3282.93	0.00	0.01	W	error
sim_64	-0.09	0.31	6923.13	0.00	0.01	W	error
sim_65	-0.18	0.58	5485.06	0.00	0.00	W	error
sim_66	0.20	0.50	6036.65	0.00	0.02	W	error
sim_67	-0.06	0.57	5617.02	0.00	0.01	W	error
sim_68	0.53	0.61	5335.49	0.00	0.01	W	error
sim_69	0.00	0.36	6621.97	0.00	0.00	W	error
sim_70	-0.25	0.53	5753.66	0.00	0.00	W	error
sim_71	0.26	0.63	5188.81	0.00	0.01	W	error
sim_72	0.43	0.49	6085.79	0.00	0.00	W	error
sim_73	0.32	0.49	6072.45	0.00	0.01	W	error
sim_74	0.04	0.32	6900.92	0.00	0.00	W	error
sim_75	-0.47	0.68	4638.20	0.00	0.01	W	error
sim_76	0.28	0.47	6174.86	0.00	0.04	S	error
sim_77	0.17	0.52	5908.11	0.00	0.00	W	error
sim_78	-0.17	0.67	4770.39	0.00	0.00	W	error

sim_79	0.24	0.61	5232.89	0.00	0.00	W	error
sim_80	-0.07	0.71	4420.87	0.00	0.01	W	error
sim_81	0.33	0.39	6500.57	0.00	0.03	S	error
sim_82	0.45	0.53	5866.47	0.00	0.00	W	error
sim_83	0.20	0.27	7061.89	0.00	0.00	W	error
sim_84	-0.20	0.55	5720.92	0.00	0.00	W	error
sim_85	0.08	0.31	6858.96	0.00	0.01	W	error
sim_86	-0.36	0.49	6090.95	0.00	0.01	W	error
sim_87	0.22	0.39	6590.64	0.00	0.01	W	error
sim_88	-0.12	0.61	5272.76	0.00	0.01	W	error
sim_89	0.33	0.60	5341.15	0.00	-0.01	W	error
sim_90	-0.21	0.27	7052.15	0.00	0.00	W	error
sim_91	0.22	0.51	5857.13	0.00	0.00	W	error
sim_92	0.18	0.55	5637.67	0.00	0.01	W	error
sim_93	0.13	0.37	6689.93	0.00	0.00	W	error
sim_94	0.18	0.51	5981.21	0.00	0.00	W	error
sim_95	-0.25	0.50	5978.67	0.00	0.00	W	error
sim_96	-0.33	0.55	5729.69	0.00	0.00	W	error
sim_97	-0.06	0.37	6651.32	0.00	0.01	W	error
sim_98	0.11	0.57	5609.05	0.00	0.00	W	error
sim_99	0.45	0.56	5608.85	0.00	0.01	W	error
sim_100	-0.05	0.51	5977.46	0.00	0.01	W	error

Leache (SARs)

Simulation	Latitude	R2	AIC	p-value	Intercept	Scheme	Model
Sim_1	-0.07	0.58	5333.36	0.00	0.00	W	error
Sim_2	-0.12	0.36	6578.65	0.00	0.00	W	error
Sim_3	0.34	0.33	6790.76	0.00	0.00	W	error
Sim_4	0.22	0.65	4907.00	0.00	-0.01	W	error
Sim_5	-0.22	0.57	5467.41	0.00	0.01	W	error
Sim_6	0.09	0.47	6226.62	0.00	0.00	W	error
Sim_7	0.27	0.42	6380.73	0.00	0.00	W	error
Sim_8	0.23	0.43	6377.78	0.00	0.00	W	error
Sim_9	-0.05	0.37	6682.52	0.00	0.01	W	error
Sim_10	0.46	0.51	5948.59	0.00	0.00	W	error
Sim_11	-0.35	0.71	4526.87	0.00	0.01	W	error
Sim_12	-0.29	0.69	4517.00	0.00	0.02	W	error
Sim_13	0.18	0.46	6159.92	0.00	0.01	W	error
Sim_14	0.19	0.59	5488.92	0.00	0.01	W	error
Sim_15	0.11	0.54	5774.68	0.00	0.01	W	error

Sim_16	0.04	0.48	6087.93	0.00	-0.03	S	error
Sim_17	-0.07	0.36	6637.30	0.00	0.01	W	error
Sim_18	-0.07	0.49	6048.25	0.00	0.01	W	error
Sim_19	0.07	0.42	6441.51	0.00	0.00	W	error
Sim_20	0.13	0.72	4350.41	0.00	0.02	W	error
Sim_21	-0.29	0.50	5932.97	0.00	0.01	W	error
Sim_22	0.20	0.43	6337.51	0.00	0.01	W	error
Sim_23	0.19	0.46	6277.81	0.00	0.00	W	error
Sim_24	-0.20	0.57	5574.67	0.00	0.00	W	error
Sim_25	0.27	0.53	5758.48	0.00	0.01	W	error
Sim_26	0.31	0.46	6264.17	0.00	0.00	W	error
Sim_27	-0.44	0.55	5651.74	0.00	0.00	W	error
Sim_28	0.35	0.53	5826.21	0.00	0.01	W	error
Sim_29	0.56	0.77	3635.52	0.00	0.00	W	error
Sim_30	-0.15	0.58	5266.90	0.00	0.03	W	error
Sim_31	0.29	0.74	3929.49	0.00	0.03	W	error
Sim_32	-0.15	0.48	6047.58	0.00	0.01	W	error
Sim_33	0.22	0.51	5969.34	0.00	0.01	W	error
Sim_34	0.60	0.61	5300.21	0.00	0.00	W	error
Sim_35	0.14	0.53	5875.75	0.00	0.01	W	error
Sim_36	-0.13	0.56	5561.19	0.00	0.00	W	error
Sim_37	0.51	0.57	5577.10	0.00	0.01	W	error
Sim_38	-0.10	0.48	6149.26	0.00	0.00	W	error
Sim_39	0.06	0.50	5968.24	0.00	0.00	W	error
Sim_40	0.52	0.49	6134.24	0.00	0.01	W	error
Sim_41	-0.39	0.60	5081.43	0.00	0.00	W	error
Sim_42	0.49	0.51	5943.77	0.00	0.00	W	error
Sim_43	-0.19	0.49	6059.48	0.00	0.00	W	error
Sim_44	0.22	0.47	6199.56	0.00	0.01	W	error
Sim_45	-0.27	0.61	5253.80	0.00	0.01	W	error
Sim_46	0.19	0.45	6245.83	0.00	0.01	W	error
Sim_47	-0.36	0.61	5162.23	0.00	0.01	W	error
Sim_48	-0.34	0.68	4785.84	0.00	0.01	W	error
Sim_49	-0.12	0.46	6246.95	0.00	0.01	W	error
Sim_50	0.44	0.69	4511.18	0.00	0.01	W	error
Sim_51	0.27	0.49	5896.67	0.00	0.02	W	error
Sim_52	0.61	0.68	4725.07	0.00	0.00	W	error
Sim_53	0.61	0.65	4870.88	0.00	0.01	W	error
Sim_54	-0.27	0.38	6616.29	0.00	0.01	W	error
Sim_55	-0.39	0.54	5835.49	0.00	0.00	W	error
Sim_56	0.05	0.55	5729.40	0.00	0.01	W	error

Sim_57	-0.05	0.61	5154.68	0.00	0.01	W	error
Sim_58	0.15	0.45	6166.66	0.00	0.01	W	error
Sim_59	-0.04	0.63	5145.25	0.00	0.01	W	error
Sim_60	0.55	0.53	5755.09	0.00	0.00	W	error
Sim_61	0.27	0.47	6158.96	0.00	0.00	W	error
Sim_62	-0.12	0.41	6533.88	0.00	0.01	W	error
Sim_63	0.50	0.81	3181.51	0.00	0.01	W	error
Sim_64	-0.07	0.37	6641.45	0.00	0.03	S	error
Sim_65	-0.09	0.55	5641.86	0.00	0.00	W	error
Sim_66	0.26	0.57	5595.16	0.00	0.17	S	error
Sim_67	-0.05	0.59	5481.93	0.00	0.01	W	error
Sim_68	0.58	0.60	5358.80	0.00	0.00	W	error
Sim_69	-0.07	0.36	6634.31	0.00	-0.01	S	error
Sim_70	-0.17	0.46	6107.01	0.00	0.00	W	error
Sim_71	0.24	0.63	5162.05	0.00	0.02	W	error
Sim_72	0.50	0.53	5823.02	0.00	0.00	W	error
Sim_73	0.23	0.59	5295.89	0.00	0.02	W	error
Sim_74	0.04	0.39	6596.10	0.00	0.00	W	error
Sim_75	-0.47	0.68	4673.40	0.00	0.01	W	error
Sim_76	0.17	0.48	6104.27	0.00	0.03	S	error
Sim_77	0.26	0.55	5738.69	0.00	0.00	W	error
Sim_78	-0.26	0.71	4377.21	0.00	0.00	W	error
Sim_79	0.07	0.67	4727.62	0.00	0.00	W	error
Sim_80	-0.05	0.68	4667.11	0.00	0.01	W	error
Sim_81	0.40	0.51	5904.28	0.00	0.00	W	error
Sim_82	0.42	0.51	5956.85	0.00	0.00	W	error
Sim_83	0.25	0.31	6891.56	0.00	0.00	W	error
Sim_84	-0.19	0.55	5754.66	0.00	0.00	W	error
Sim_85	0.15	0.36	6667.41	0.00	0.01	W	error
Sim_86	-0.31	0.45	6249.72	0.00	0.01	W	error
Sim_87	0.21	0.45	6314.36	0.00	0.01	W	error
Sim_88	-0.08	0.64	5016.87	0.00	0.02	W	error
Sim_89	0.33	0.58	5521.80	0.00	-0.01	W	error
Sim_90	-0.25	0.30	6922.98	0.00	0.00	W	error
Sim_91	0.13	0.47	5936.42	0.00	0.01	W	error
Sim_92	0.21	0.53	5707.30	0.00	0.02	W	error
Sim_93	0.22	0.42	6463.10	0.00	0.01	W	error
Sim_94	0.08	0.41	6478.32	0.00	0.00	W	error
Sim_95	-0.20	0.46	6185.20	0.00	0.00	W	error
Sim_96	-0.29	0.59	5469.44	0.00	0.01	W	error
Sim_97	-0.09	0.36	6680.56	0.00	0.01	W	error

Sim_98	0.12	0.59	5488.88	0.00	0.00	W	error
Sim_99	0.47	0.60	5303.22	0.00	0.02	W	error
Sim_100	0.00	0.46	6210.93	0.00	-0.02	S	error
