# Supplementary material for: Exploring the causes underlying the latitudinal variation in range sizes: Evidence for Rapoport’s rule in spiny lizards (genus Sceloporus)
Source: PLoS One. 2024 Jul 9;19(7):e0306832. doi: 10.1371/journal.pone.0306832 (PMC11233011; doi:10.1371/journal.pone.0306832)
Supplement: S4 Appendix — These tables contain all the simulated coefficients for the relation between range size and every environmental hypothesis. (PDF) [file pone.0306832.s004.pdf]

S4 Appendix. Coefficients for null simulations for climatic hypothesis

All species (OLS – Species level)

Simulation	CEH min	CVH	CCV	Elevation	R2	p-value	AIC	BIC
Sim_1	0.15	0.15	0.01	0.01	0.01	0.26	296.53	307.07
Sim_2	0.00	0.02	0.13	0.23	0.05	0.03	292.14	302.68
Sim_3	0.00	-0.08	0.08	-0.06	0.01	0.12	294.85	302.75
Sim_4	0.05	0.04	-0.04	0.10	0.01	0.12	294.82	302.72
Sim_5	-0.01	0.03	-0.08	-0.09	-0.01	0.52	296.87	304.78
Sim_6	0.02	-0.02	0.02	0.10	0.00	0.24	295.86	303.76
Sim_7	-0.03	0.01	0.25	-0.04	0.08	0.00	287.74	295.65
Sim_8	-0.04	0.05	0.00	0.02	0.00	0.27	296.04	303.95
Sim_9	-0.01	0.04	0.00	0.18	0.04	0.02	292.10	300.01
Sim_10	0.04	-0.08	0.13	-0.17	0.08	0.00	287.93	295.83
Sim_11	0.02	0.03	0.05	0.00	0.00	0.34	296.37	304.27
Sim_12	0.38	0.56	-0.12	-0.01	0.06	0.02	291.59	304.77
Sim_13	-0.05	-0.03	0.02	0.01	-0.01	0.61	297.04	304.94
Sim_14	0.13	0.17	0.08	0.10	0.01	0.21	295.67	303.57
Sim_15	-0.33	-0.30	-0.08	-0.05	0.02	0.17	295.60	306.14
Sim_16	0.15	0.19	0.02	-0.01	0.01	0.21	296.05	306.58
Sim_17	0.03	0.05	-0.01	-0.01	-0.01	0.69	297.13	305.04
Sim_18	-0.01	0.02	0.06	0.01	0.00	0.28	296.09	303.99
Sim_19	-0.04	0.13	0.05	-0.01	0.03	0.04	292.88	300.79
Sim_20	0.05	0.05	-0.01	0.01	-0.01	0.73	297.17	305.08
Sim_21	0.02	0.02	-0.02	0.03	0.00	0.45	296.71	304.62
Sim_22	0.02	0.06	0.01	0.05	0.00	0.34	296.38	304.28
Sim_23	0.02	0.00	0.04	0.00	-0.01	0.52	296.88	304.78
Sim_24	-0.15	-0.27	0.10	0.14	0.03	0.12	295.20	308.38
Sim_25	-0.01	0.05	0.04	-0.01	0.00	0.26	296.01	303.91
Sim_26	0.00	0.03	0.05	-0.02	0.00	0.28	296.10	304.00
Sim_27	0.19	0.17	-0.04	0.03	0.00	0.41	296.60	304.51
Sim_28	-0.05	0.03	0.16	0.00	0.04	0.02	292.07	299.98
Sim_29	0.02	-0.03	0.19	0.05	0.02	0.10	294.50	302.40
Sim_30	-0.04	0.03	0.08	0.05	0.01	0.20	295.62	303.52
Sim_31	0.02	0.00	-0.09	-0.09	0.00	0.35	296.41	304.32
Sim_32	0.00	-0.05	-0.04	0.00	0.00	0.31	296.26	304.16
Sim_33	0.02	-0.01	-0.13	0.04	0.03	0.05	293.27	301.17
Sim_34	-0.01	0.00	-0.02	0.04	0.00	0.38	296.51	304.41
Sim_35	0.09	0.07	-0.03	0.02	0.00	0.45	296.72	304.62
Sim_36	0.00	0.02	0.07	0.05	0.00	0.40	296.56	304.46
Sim_37	0.13	0.11	0.04	-0.15	0.04	0.03	292.61	300.51

Sim_38	-0.01	0.05	-0.10	-0.21	0.02	0.11	294.69	302.59
Sim_39	-0.14	-0.08	0.02	0.01	0.00	0.33	296.31	304.22
Sim_40	0.20	0.20	0.03	-0.01	0.02	0.17	295.66	306.20
Sim_41	0.09	-0.03	0.02	-0.01	0.01	0.17	295.34	303.25
Sim_42	-0.28	-0.13	-0.04	0.01	0.02	0.09	294.43	302.33
Sim_43	0.09	0.02	0.03	0.02	0.00	0.37	296.47	304.37
Sim_44	-0.08	-0.04	-0.03	0.00	-0.01	0.55	296.94	304.84
Sim_45	0.08	-0.05	0.15	0.09	0.02	0.20	296.54	309.72
Sim_46	-0.02	0.02	0.02	0.00	0.00	0.48	296.79	304.69
Sim_47	0.00	0.02	0.04	0.00	0.00	0.42	296.62	304.52
Sim_48	0.16	0.20	0.01	0.00	0.01	0.20	296.01	306.55
Sim_49	0.14	-0.09	0.04	0.09	0.03	0.05	293.48	301.38
Sim_50	-0.20	0.01	0.05	0.04	0.06	0.01	290.21	298.12
Sim_51	0.02	-0.03	-0.12	0.01	0.02	0.08	294.05	301.96
Sim_52	0.00	0.02	0.02	0.05	0.00	0.47	296.76	304.67
Sim_53	0.00	0.05	0.03	-0.02	0.00	0.41	296.59	304.49
Sim_54	0.21	0.23	0.00	0.01	0.01	0.23	296.29	306.83
Sim_55	0.45	0.42	0.02	-0.02	0.02	0.13	295.03	305.57
Sim_56	0.03	0.00	0.26	0.13	0.03	0.06	293.53	301.44
Sim_57	0.09	-0.01	-0.02	0.02	0.01	0.16	295.27	303.18
Sim_58	0.01	0.02	0.05	-0.05	0.01	0.21	295.67	303.58
Sim_59	0.17	0.22	0.01	0.00	0.01	0.18	295.78	306.31
Sim_60	0.09	0.03	0.11	0.25	0.03	0.05	293.43	301.33
Sim_61	-0.06	0.06	0.02	-0.01	0.01	0.13	294.99	302.89
Sim_62	-0.36	-0.35	0.01	0.00	0.02	0.11	294.73	305.27
Sim_63	-0.04	-0.03	0.07	0.00	0.00	0.24	295.86	303.76
Sim_64	-0.12	-0.10	0.00	0.01	-0.01	0.61	297.04	304.94
Sim_65	0.07	0.08	0.36	0.41	0.09	0.00	287.47	298.01
Sim_66	-0.08	0.06	0.08	0.00	0.03	0.05	293.29	301.19
Sim_67	-0.05	0.00	0.04	0.06	0.00	0.25	295.94	303.85
Sim_68	0.40	0.40	-0.03	0.03	0.02	0.14	295.24	305.78
Sim_69	0.03	0.00	-0.06	0.01	0.00	0.27	296.02	303.93
Sim_70	0.01	0.06	-0.24	-0.15	0.01	0.19	295.55	303.45
Sim_71	0.26	0.12	0.03	-0.01	0.02	0.09	294.43	302.34
Sim_72	0.00	0.02	0.01	0.14	0.02	0.07	294.02	301.93
Sim_73	0.12	0.16	0.11	0.10	0.01	0.15	295.15	303.06
Sim_74	0.12	0.04	0.02	0.03	0.00	0.34	296.37	304.27
Sim_75	-0.03	-0.06	0.06	0.00	0.00	0.43	296.65	304.55
Sim_76	-0.01	0.00	0.00	0.02	-0.01	0.64	297.07	304.98
Sim_77	0.00	0.01	0.00	-0.03	-0.01	0.58	296.97	304.88
Sim_78	0.01	-0.01	0.03	0.05	-0.01	0.56	296.94	304.85

Sim_79	0.80	0.82	0.04	0.02	0.06	0.02	290.88	301.42
Sim_80	1.12	0.91	0.00	0.08	0.10	0.00	286.15	296.69
Sim_81	0.14	0.04	-0.03	0.09	0.02	0.11	294.78	305.31
Sim_82	-0.03	-0.05	0.00	0.00	-0.01	0.61	297.02	304.93
Sim_83	-0.05	0.04	-0.16	-0.14	-0.01	0.54	296.91	304.82
Sim_84	0.00	0.01	0.05	0.09	0.00	0.36	296.44	304.34
Sim_85	0.06	0.04	0.01	0.07	0.00	0.32	296.28	304.18
Sim_86	0.07	-0.03	0.01	0.03	0.01	0.21	295.67	303.58
Sim_87	-0.11	-0.16	-0.02	0.00	0.00	0.34	296.36	304.26
Sim_88	0.00	0.03	0.14	-0.02	0.03	0.04	293.08	300.99
Sim_89	0.00	0.01	0.04	0.08	0.00	0.31	296.22	304.13
Sim_90	0.00	0.00	0.03	0.03	-0.01	0.66	297.10	305.00
Sim_91	0.00	0.01	-0.02	0.15	0.03	0.04	293.05	300.96
Sim_92	-0.02	0.09	-0.05	-0.05	0.00	0.26	295.97	303.88
Sim_93	-0.08	0.04	0.04	-0.01	0.01	0.13	294.91	302.81
Sim_94	0.02	0.01	0.04	-0.05	0.00	0.27	296.04	303.95
Sim_95	-0.04	-0.01	-0.02	-0.01	-0.01	0.63	297.06	304.97
Sim_96	-0.12	0.00	-0.07	-0.21	0.01	0.14	295.07	302.97
Sim_97	-0.02	0.05	-0.01	-0.04	0.00	0.45	296.70	304.61
Sim_98	0.15	0.07	0.26	0.01	0.05	0.02	291.40	299.31
Sim_99	0.09	0.18	0.00	0.05	0.01	0.18	295.48	303.39
Sim_100	0.05	-0.04	0.15	0.07	0.00	0.23	295.83	303.73

Tonini (OLS – species level)

Simulation	CEH min	CVH	Elevation	CCV	R2	p-value	AIC	BIC
Sim_1	0.92	0.87	0.07	0.03	0.08	0.01	258.80	268.89
Sim_2	0.15	0.06	0.44	0.29	0.07	0.01	259.04	269.12
Sim_3	0.02	-0.07	-0.01	0.03	0.00	0.29	264.94	272.51
Sim_4	0.01	0.03	0.16	0.01	0.03	0.06	262.35	269.91
Sim_5	-0.04	0.04	-0.24	-0.18	0.01	0.21	264.47	272.04
Sim_6	0.01	0.00	0.03	-0.02	-0.01	0.51	265.62	273.19
Sim_7	-0.09	0.02	-0.11	0.16	0.07	0.01	258.16	265.72
Sim_8	-0.04	0.08	-0.03	0.00	0.01	0.18	264.23	271.80
Sim_9	-0.02	0.04	0.16	-0.01	0.04	0.03	261.41	268.97
Sim_10	0.10	-0.02	-0.12	0.09	0.05	0.02	260.80	268.36
Sim_11	0.00	0.02	0.00	0.01	-0.01	0.75	265.97	273.54
Sim_12	0.07	0.32	0.03	-0.08	0.06	0.02	260.21	270.29
Sim_13	0.00	-0.02	-0.01	0.03	-0.01	0.59	265.78	273.35
Sim_14	0.11	0.12	0.04	0.01	-0.01	0.55	265.71	273.28
Sim_15	-0.11	-0.12	-0.08	-0.07	-0.01	0.49	265.59	273.15

Sim_16	0.19	0.14	0.03	0.07	0.01	0.27	265.36	275.44
Sim_17	0.05	0.09	0.00	0.03	0.00	0.42	265.40	272.97
Sim_18	0.00	-0.03	-0.01	0.07	0.00	0.30	264.98	272.54
Sim_19	-0.06	0.09	0.01	0.07	0.03	0.06	262.44	270.00
Sim_20	0.00	0.01	0.05	0.00	0.00	0.39	265.32	272.89
Sim_21	0.02	0.02	0.03	0.01	-0.01	0.63	265.85	273.41
Sim_22	0.01	0.10	0.03	0.03	0.01	0.18	264.26	271.82
Sim_23	0.11	0.12	-0.01	0.01	-0.01	0.69	265.92	273.48
Sim_24	0.01	-0.05	0.06	0.02	0.00	0.42	265.41	272.97
Sim_25	-0.01	0.01	0.00	0.05	0.00	0.39	265.30	272.87
Sim_26	0.06	0.12	0.00	0.02	0.00	0.29	264.92	272.48
Sim_27	0.09	0.05	0.05	-0.04	0.00	0.32	265.05	272.61
Sim_28	-0.02	0.04	0.02	0.13	0.02	0.08	262.87	270.44
Sim_29	0.04	-0.06	0.14	0.30	0.02	0.09	263.06	270.63
Sim_30	-0.05	0.03	0.09	0.12	0.03	0.10	263.33	273.41
Sim_31	0.02	0.02	-0.04	-0.02	-0.01	0.57	265.75	273.32
Sim_32	0.00	0.00	0.00	-0.11	0.01	0.17	264.10	271.67
Sim_33	0.04	-0.02	0.02	-0.09	0.02	0.09	263.17	270.73
Sim_34	-0.03	0.00	0.04	0.01	-0.01	0.47	265.54	273.10
Sim_35	0.02	0.03	0.01	0.00	-0.01	0.74	265.97	273.53
Sim_36	0.00	0.00	0.09	0.05	0.00	0.35	265.17	272.73
Sim_37	1.04	0.96	-0.09	0.03	0.09	0.01	258.61	271.22
Sim_38	0.01	0.04	-0.23	-0.02	0.04	0.03	261.26	268.83
Sim_39	-0.11	-0.03	0.00	0.02	0.00	0.24	264.65	272.21
Sim_40	0.07	0.12	0.04	0.03	0.00	0.31	265.01	272.58
Sim_41	0.23	0.06	-0.01	0.10	0.04	0.07	262.51	272.60
Sim_42	-0.55	-0.32	-0.01	-0.07	0.06	0.03	260.70	270.78
Sim_43	0.06	0.01	-0.01	0.06	0.00	0.43	265.45	273.01
Sim_44	-0.05	0.00	-0.03	0.00	-0.01	0.53	265.67	273.24
Sim_45	0.17	-0.17	0.34	0.48	0.06	0.03	260.98	273.59
Sim_46	-0.17	-0.03	-0.02	0.04	0.02	0.08	262.98	270.55
Sim_47	-0.01	0.01	0.05	0.01	0.00	0.46	265.52	273.09
Sim_48	0.08	0.06	-0.01	0.00	-0.01	0.72	265.95	273.51
Sim_49	0.16	-0.02	0.10	0.01	0.03	0.10	263.39	273.48
Sim_50	-0.05	0.10	0.05	0.07	0.03	0.05	262.03	269.59
Sim_51	0.01	-0.07	0.11	-0.14	0.05	0.02	260.27	267.83
Sim_52	0.02	0.06	0.00	0.00	-0.01	0.48	265.57	273.13
Sim_53	0.00	0.07	-0.02	0.06	0.01	0.19	264.34	271.91
Sim_54	0.11	0.16	0.02	-0.01	0.00	0.40	265.35	272.91
Sim_55	0.27	0.27	0.00	0.01	0.01	0.22	264.93	275.02
Sim_56	0.02	-0.02	0.19	0.33	0.05	0.04	261.21	271.30

Sim_57	0.30	0.08	0.11	-0.02	0.06	0.02	260.12	270.21
Sim_58	0.01	0.00	-0.08	0.11	0.03	0.06	262.56	270.13
Sim_59	0.08	0.13	0.03	-0.01	0.00	0.43	265.45	273.02
Sim_60	0.03	0.03	0.17	0.12	0.02	0.14	263.94	274.03
Sim_61	-0.03	0.10	0.01	0.02	0.02	0.10	263.33	270.90
Sim_62	-0.21	-0.18	-0.01	0.05	0.01	0.21	264.84	274.93
Sim_63	0.01	-0.05	-0.01	0.02	0.00	0.45	265.50	273.06
Sim_64	-0.06	0.01	0.01	0.01	0.00	0.34	265.15	272.71
Sim_65	0.41	0.63	0.08	0.02	0.08	0.01	258.63	268.71
Sim_66	-0.03	0.08	-0.01	0.05	0.02	0.12	263.63	271.20
Sim_67	-0.07	0.00	0.08	0.04	0.01	0.21	264.44	272.00
Sim_68	0.36	0.33	0.02	0.00	0.02	0.15	264.14	274.22
Sim_69	0.04	0.00	0.01	-0.03	0.00	0.41	265.39	272.96
Sim_70	0.16	0.15	-0.09	-0.19	0.01	0.14	263.85	271.42
Sim_71	0.14	-0.01	0.05	0.06	0.01	0.20	264.40	271.96
Sim_72	-0.02	0.03	0.18	0.04	0.03	0.05	262.24	269.80
Sim_73	0.44	0.45	0.18	0.09	0.05	0.06	262.23	274.84
Sim_74	0.22	0.08	0.18	0.18	0.03	0.14	264.36	276.97
Sim_75	0.03	-0.01	0.02	0.10	0.00	0.28	264.86	272.42
Sim_76	-0.01	-0.02	0.02	-0.02	-0.01	0.57	265.75	273.31
Sim_77	-0.01	0.02	-0.03	-0.02	-0.01	0.73	265.96	273.53
Sim_78	0.00	-0.01	0.06	0.02	0.00	0.38	265.28	272.85
Sim_79	0.50	0.56	0.01	0.04	0.04	0.05	261.93	272.02
Sim_80	0.95	0.77	0.10	0.03	0.07	0.02	259.81	269.90
Sim_81	0.11	0.02	0.01	-0.03	0.01	0.18	264.23	271.80
Sim_82	-0.01	-0.08	-0.01	0.05	0.00	0.35	265.18	272.74
Sim_83	-0.01	0.03	-0.06	-0.04	-0.01	0.57	265.74	273.31
Sim_84	0.02	0.04	0.04	0.02	-0.01	0.57	265.74	273.31
Sim_85	0.08	0.10	0.06	0.02	0.00	0.35	265.19	272.76
Sim_86	0.07	-0.01	0.01	0.00	0.00	0.31	265.01	272.58
Sim_87	0.00	-0.03	-0.02	-0.04	-0.01	0.51	265.64	273.20
Sim_88	0.02	0.03	-0.03	0.11	0.02	0.09	263.13	270.69
Sim_89	-0.02	0.04	0.02	-0.02	0.00	0.45	265.50	273.06
Sim_90	-0.01	-0.01	0.06	0.02	0.00	0.41	265.37	272.94
Sim_91	0.02	0.00	0.19	-0.04	0.04	0.03	261.04	268.61
Sim_92	-0.07	0.15	-0.04	-0.06	0.03	0.06	262.39	269.96
Sim_93	-0.03	0.09	-0.03	0.04	0.01	0.14	263.84	271.41
Sim_94	0.06	0.07	-0.04	0.08	0.01	0.13	263.70	271.27
Sim_95	-0.14	-0.18	0.01	-0.03	0.01	0.19	264.66	274.74
Sim_96	-0.05	0.05	-0.21	-0.14	0.00	0.33	265.08	272.65
Sim_97	0.00	0.05	-0.03	0.01	0.00	0.46	265.51	273.08

Sim_98	0.14	0.08	0.01	0.19	0.02	0.07	262.74	270.31
Sim_99	0.25	0.34	0.01	-0.03	0.02	0.16	264.27	274.36
Sim_100	-0.03	-0.13	0.11	0.25	0.04	0.10	263.48	276.09

Leache (OLS – species level)

Simulation	CEH min	CVH	Elevation	CCV	R2	p-value	AIC	BIC
Sim_1	0.99	1.01	0.01	0.00	0.10	0.01	226.02	235.59
Sim_2	0.13	0.07	0.32	0.23	0.05	0.06	231.29	243.26
Sim_3	0.05	-0.04	-0.03	0.01	0.00	0.28	233.68	240.86
Sim_4	0.03	0.03	0.10	0.00	0.01	0.17	232.91	240.10
Sim_5	-0.08	0.03	-0.45	-0.37	0.06	0.05	230.83	242.80
Sim_6	0.01	0.02	0.02	-0.01	-0.01	0.59	234.57	241.75
Sim_7	-0.09	0.01	-0.06	0.19	0.07	0.01	227.77	234.95
Sim_8	-0.02	0.06	0.02	-0.03	0.00	0.32	233.86	241.04
Sim_9	-0.02	0.00	0.19	-0.02	0.04	0.03	230.26	237.44
Sim_10	0.11	0.01	-0.09	0.11	0.04	0.05	230.92	238.10
Sim_11	-0.02	0.07	0.00	0.02	0.01	0.23	233.41	240.59
Sim_12	0.16	0.42	0.03	-0.11	0.08	0.02	228.60	240.57
Sim_13	-0.02	0.01	-0.01	0.04	0.00	0.41	234.17	241.35
Sim_14	0.09	0.04	0.00	0.01	-0.01	0.47	234.33	241.52
Sim_15	-0.08	-0.03	-0.05	-0.07	-0.01	0.66	234.67	241.85
Sim_16	0.05	0.05	0.01	0.06	0.00	0.37	234.02	241.20
Sim_17	0.00	0.07	0.01	0.02	0.00	0.33	233.90	241.08
Sim_18	-0.02	-0.05	0.01	0.07	-0.01	0.45	234.27	241.45
Sim_19	-0.13	0.06	-0.02	0.05	0.04	0.04	230.71	237.90
Sim_20	-0.01	0.00	0.08	0.07	-0.01	0.49	234.38	241.56
Sim_21	-0.02	0.01	0.05	0.02	-0.01	0.46	234.31	241.49
Sim_22	-0.02	0.05	0.06	0.03	0.00	0.28	233.64	240.82
Sim_23	0.07	0.06	-0.03	0.01	-0.01	0.51	234.41	241.59
Sim_24	-0.04	-0.06	0.12	0.05	0.00	0.25	233.50	240.68
Sim_25	-0.04	0.00	0.00	0.04	0.00	0.39	234.10	241.29
Sim_26	0.02	0.12	0.01	0.03	0.01	0.19	233.07	240.25
Sim_27	0.09	0.04	0.09	-0.06	0.01	0.14	232.66	239.84
Sim_28	-0.03	0.06	0.01	0.08	0.01	0.15	232.77	239.96
Sim_29	0.06	-0.08	0.15	0.31	0.02	0.13	232.49	239.67
Sim_30	-0.02	0.03	0.11	0.17	0.04	0.09	231.90	241.47
Sim_31	0.01	0.05	0.00	-0.03	-0.01	0.71	234.72	241.90
Sim_32	-0.01	-0.02	0.01	-0.07	0.00	0.28	233.65	240.83
Sim_33	0.03	-0.02	0.02	-0.09	0.02	0.14	232.58	239.76
Sim_34	-0.05	-0.01	0.04	-0.02	0.00	0.34	233.92	241.11

Sim_35	0.02	0.07	0.02	0.00	0.00	0.44	234.25	241.43
Sim_36	-0.03	0.03	0.11	0.11	0.02	0.14	232.81	242.39
Sim_37	0.63	0.59	-0.08	0.02	0.05	0.05	230.68	240.26
Sim_38	0.00	0.03	-0.27	-0.06	0.05	0.03	230.09	237.27
Sim_39	-0.08	0.02	0.03	0.15	0.03	0.07	231.48	238.67
Sim_40	0.15	0.15	0.09	0.05	0.00	0.36	234.00	241.18
Sim_41	0.40	0.23	-0.01	0.06	0.04	0.09	231.83	241.41
Sim_42	-0.44	-0.19	0.00	-0.03	0.06	0.02	229.15	236.34
Sim_43	0.04	-0.01	0.02	0.03	-0.01	0.58	234.54	241.73
Sim_44	-0.06	0.01	-0.02	-0.03	-0.01	0.49	234.37	241.55
Sim_45	0.12	-0.37	0.58	0.68	0.11	0.01	226.66	238.63
Sim_46	-0.10	0.00	-0.02	0.08	0.02	0.14	232.57	239.75
Sim_47	-0.02	0.02	0.05	0.01	-0.01	0.45	234.27	241.46
Sim_48	0.09	0.09	-0.02	0.02	-0.01	0.52	234.43	241.61
Sim_49	0.13	0.00	0.04	-0.02	0.01	0.15	232.72	239.91
Sim_50	-0.03	0.09	0.09	0.08	0.03	0.07	231.48	238.66
Sim_51	-0.05	-0.27	0.19	-0.10	0.11	0.00	225.69	235.27
Sim_52	0.05	0.07	0.00	-0.01	-0.01	0.64	234.63	241.81
Sim_53	0.01	0.05	0.00	0.03	-0.01	0.46	234.30	241.49
Sim_54	0.08	0.15	0.02	0.00	0.00	0.33	233.90	241.08
Sim_55	0.24	0.20	0.00	0.00	0.01	0.29	234.27	243.85
Sim_56	0.02	-0.05	0.17	0.29	0.02	0.11	232.24	239.42
Sim_57	0.58	0.34	0.08	0.01	0.08	0.01	228.04	237.62
Sim_58	0.04	0.01	-0.09	0.08	0.02	0.10	232.01	239.19
Sim_59	0.03	0.05	0.05	0.02	-0.01	0.48	234.35	241.53
Sim_60	0.07	0.02	0.35	0.16	0.05	0.02	229.63	236.81
Sim_61	-0.04	0.07	0.02	0.00	0.01	0.21	233.22	240.41
Sim_62	-0.15	-0.10	0.00	0.03	0.00	0.37	234.02	241.21
Sim_63	0.05	-0.02	-0.03	0.02	0.00	0.37	234.04	241.23
Sim_64	-0.01	0.02	0.01	0.00	-0.01	0.74	234.75	241.93
Sim_65	0.45	0.73	0.10	0.10	0.14	0.00	222.58	232.16
Sim_66	-0.04	0.06	0.01	0.06	0.01	0.17	232.88	240.06
Sim_67	-0.02	-0.02	0.05	0.03	-0.01	0.51	234.41	241.59
Sim_68	0.49	0.44	0.02	0.00	0.03	0.10	232.12	241.70
Sim_69	0.01	-0.05	-0.03	-0.07	0.00	0.27	233.62	240.81
Sim_70	0.20	0.24	-0.13	-0.22	0.03	0.17	233.68	245.65
Sim_71	0.11	0.02	0.02	0.02	0.00	0.31	233.79	240.98
Sim_72	0.00	0.06	0.15	0.06	0.03	0.12	232.42	242.00
Sim_73	0.19	0.20	0.23	0.12	0.04	0.08	231.63	241.21
Sim_74	0.06	0.06	0.03	0.07	0.00	0.42	234.20	241.39
Sim_75	0.02	-0.02	-0.02	0.11	0.01	0.20	233.16	240.34

Sim_76	0.00	-0.01	0.02	0.00	-0.01	0.73	234.74	241.92
Sim_77	0.00	-0.01	-0.03	-0.01	-0.01	0.72	234.72	241.91
Sim_78	0.00	0.00	0.05	0.00	0.00	0.43	234.23	241.41
Sim_79	0.69	0.79	-0.01	0.03	0.07	0.02	228.69	238.27
Sim_80	1.08	0.92	0.19	0.04	0.09	0.01	227.76	239.74
Sim_81	0.04	-0.01	0.00	-0.05	0.00	0.30	233.74	240.92
Sim_82	-0.08	-0.15	0.01	0.05	0.00	0.38	234.08	241.26
Sim_83	-0.01	0.02	-0.12	-0.08	0.00	0.36	234.01	241.20
Sim_84	0.02	-0.02	0.07	0.03	0.00	0.41	234.16	241.34
Sim_85	0.08	0.10	0.14	0.12	0.02	0.18	233.32	242.90
Sim_86	0.08	-0.03	0.03	-0.02	0.01	0.19	233.11	240.29
Sim_87	0.02	-0.05	-0.02	-0.04	0.00	0.31	233.81	240.99
Sim_88	0.02	0.00	-0.03	0.15	0.03	0.08	231.69	238.87
Sim_89	-0.02	0.06	0.01	-0.01	0.00	0.33	233.88	241.06
Sim_90	-0.02	-0.02	0.02	0.03	-0.01	0.74	234.74	241.93
Sim_91	0.01	0.04	0.21	-0.07	0.07	0.01	228.20	235.39
Sim_92	-0.08	0.17	-0.04	-0.04	0.04	0.04	230.40	237.58
Sim_93	0.09	0.25	-0.05	0.03	0.03	0.07	231.45	238.64
Sim_94	0.01	0.01	-0.07	0.09	0.02	0.13	232.54	239.73
Sim_95	-0.20	-0.33	-0.01	-0.02	0.04	0.08	231.55	241.12
Sim_96	-0.03	0.03	-0.13	-0.06	0.00	0.32	233.86	241.04
Sim_97	-0.01	0.11	-0.06	0.04	0.01	0.16	232.79	239.97
Sim_98	0.07	0.02	-0.08	0.11	0.02	0.09	231.96	239.14
Sim_99	0.22	0.30	0.00	-0.03	0.01	0.22	233.71	243.28
Sim_100	-0.04	-0.24	0.29	0.39	0.07	0.04	230.11	242.09

Tonini (PGLS)

Simulation	CEH min	CVH	Elevation	CCV	R2	p-value
Sim_1	0.92	0.87	0.07	0.03	0.08	0.00
Sim_2	0.15	0.06	0.44	0.29	0.07	0.05
Sim_3	0.02	-0.07	-0.01	0.03	0.00	0.29
Sim_4	0.01	0.03	0.16	0.01	0.03	0.06
Sim_5	-0.04	0.04	-0.24	-0.18	0.01	0.21
Sim_6	0.01	0.00	0.03	-0.02	-0.01	0.51
Sim_7	-0.09	0.02	-0.11	0.16	0.07	0.01
Sim_8	-0.04	0.08	-0.03	0.00	0.01	0.18
Sim_9	-0.02	0.04	0.16	-0.01	0.04	0.03
Sim_10	0.10	-0.02	-0.12	0.09	0.05	0.02
Sim_11	0.00	0.02	0.00	0.01	-0.01	0.75
Sim_12	0.07	0.32	0.03	-0.08	0.06	0.15

Sim_13	0.00	-0.02	-0.01	0.03	-0.01	0.59
Sim_14	0.11	0.12	0.04	0.01	-0.01	0.55
Sim_15	-0.11	-0.12	-0.08	-0.07	-0.01	0.49
Sim_16	0.19	0.14	0.03	0.07	0.01	0.11
Sim_17	0.05	0.09	0.00	0.03	0.00	0.42
Sim_18	0.00	-0.03	-0.01	0.07	0.00	0.30
Sim_19	-0.06	0.09	0.01	0.07	0.03	0.06
Sim_20	0.00	0.01	0.05	0.00	0.00	0.39
Sim_21	0.02	0.02	0.03	0.01	-0.01	0.63
Sim_22	0.01	0.10	0.03	0.03	0.01	0.18
Sim_23	0.11	0.12	-0.01	0.01	-0.01	0.69
Sim_24	0.01	-0.05	0.06	0.02	0.00	0.42
Sim_25	-0.01	0.01	0.00	0.05	0.00	0.39
Sim_26	0.06	0.12	0.00	0.02	0.00	0.29
Sim_27	0.09	0.05	0.05	-0.04	0.00	0.32
Sim_28	-0.02	0.04	0.02	0.13	0.02	0.08
Sim_29	0.04	-0.06	0.14	0.30	0.02	0.09
Sim_30	-0.05	0.03	0.09	0.12	0.03	0.04
Sim_31	0.02	0.02	-0.04	-0.02	-0.01	0.57
Sim_32	0.00	0.00	0.00	-0.11	0.01	0.17
Sim_33	0.04	-0.02	0.02	-0.09	0.02	0.09
Sim_34	-0.03	0.00	0.04	0.01	-0.01	0.47
Sim_35	0.02	0.03	0.01	0.00	-0.01	0.74
Sim_36	0.00	0.00	0.09	0.05	0.00	0.35
Sim_37	1.04	0.96	-0.09	0.03	0.09	0.01
Sim_38	0.01	0.04	-0.23	-0.02	0.04	0.03
Sim_39	-0.11	-0.03	0.00	0.02	0.00	0.24
Sim_40	0.07	0.12	0.04	0.03	0.00	0.31
Sim_41	0.23	0.06	-0.01	0.10	0.04	0.10
Sim_42	-0.55	-0.32	-0.01	-0.07	0.06	0.03
Sim_43	0.06	0.01	-0.01	0.06	0.00	0.43
Sim_44	-0.05	0.00	-0.03	0.00	-0.01	0.53
Sim_45	0.17	-0.17	0.34	0.48	0.06	0.00
Sim_46	-0.17	-0.03	-0.02	0.04	0.02	0.08
Sim_47	-0.01	0.01	0.05	0.01	0.00	0.46
Sim_48	0.08	0.06	-0.01	0.00	-0.01	0.72
Sim_49	0.16	-0.02	0.10	0.01	0.03	0.05
Sim_50	-0.05	0.10	0.05	0.07	0.03	0.05
Sim_51	0.01	-0.07	0.11	-0.14	0.05	0.02
Sim_52	0.02	0.06	0.00	0.00	-0.01	0.48
Sim_53	0.00	0.07	-0.02	0.06	0.01	0.19

Sim_54	0.11	0.16	0.02	-0.01	0.00	0.40
Sim_55	0.27	0.27	0.00	0.01	0.01	0.09
Sim_56	0.02	-0.02	0.19	0.33	0.05	0.01
Sim_57	0.30	0.08	0.11	-0.02	0.06	0.01
Sim_58	0.01	0.00	-0.08	0.11	0.03	0.06
Sim_59	0.08	0.13	0.03	-0.01	0.00	0.43
Sim_60	0.03	0.03	0.17	0.12	0.02	0.14
Sim_61	-0.03	0.10	0.01	0.02	0.02	0.10
Sim_62	-0.21	-0.18	-0.01	0.05	0.01	0.08
Sim_63	0.01	-0.05	-0.01	0.02	0.00	0.45
Sim_64	-0.06	0.01	0.01	0.01	0.00	0.34
Sim_65	0.41	0.63	0.08	0.02	0.08	0.09
Sim_66	-0.03	0.08	-0.01	0.05	0.02	0.12
Sim_67	-0.07	0.00	0.08	0.04	0.01	0.21
Sim_68	0.36	0.33	0.02	0.00	0.02	0.05
Sim_69	0.04	0.00	0.01	-0.03	0.00	0.41
Sim_70	0.16	0.15	-0.09	-0.19	0.01	0.14
Sim_71	0.14	-0.01	0.05	0.06	0.01	0.20
Sim_72	-0.02	0.03	0.18	0.04	0.03	0.05
Sim_73	0.44	0.45	0.18	0.09	0.05	0.03
Sim_74	0.22	0.08	0.18	0.18	0.03	0.02
Sim_75	0.03	-0.01	0.02	0.10	0.00	0.28
Sim_76	-0.01	-0.02	0.02	-0.02	-0.01	0.57
Sim_77	-0.01	0.02	-0.03	-0.02	-0.01	0.73
Sim_78	0.00	-0.01	0.06	0.02	0.00	0.38
Sim_79	0.50	0.56	0.01	0.04	0.04	0.03
Sim_80	0.95	0.77	0.10	0.03	0.07	0.01
Sim_81	0.11	0.02	0.01	-0.03	0.01	0.18
Sim_82	-0.01	-0.08	-0.01	0.05	0.00	0.35
Sim_83	-0.01	0.03	-0.06	-0.04	-0.01	0.57
Sim_84	0.02	0.04	0.04	0.02	-0.01	0.57
Sim_85	0.08	0.10	0.06	0.02	0.00	0.35
Sim_86	0.07	-0.01	0.01	0.00	0.00	0.31
Sim_87	0.00	-0.03	-0.02	-0.04	-0.01	0.51
Sim_88	0.02	0.03	-0.03	0.11	0.02	0.09
Sim_89	-0.02	0.04	0.02	-0.02	0.00	0.45
Sim_90	-0.01	-0.01	0.06	0.02	0.00	0.41
Sim_91	0.02	0.00	0.19	-0.04	0.04	0.03
Sim_92	-0.07	0.15	-0.04	-0.06	0.03	0.06
Sim_93	-0.03	0.09	-0.03	0.04	0.01	0.14
Sim_94	0.06	0.07	-0.04	0.08	0.01	0.13

Sim_95	-0.14	-0.18	0.01	-0.03	0.01	0.12
Sim_96	-0.05	0.05	-0.21	-0.14	0.00	0.33
Sim_97	0.00	0.05	-0.03	0.01	0.00	0.46
Sim_98	0.14	0.08	0.01	0.19	0.02	0.07
Sim_99	0.25	0.34	0.01	-0.03	0.02	0.11
Sim_100	-0.03	-0.13	0.11	0.25	0.04	0.02

Leache (PGLS)

Simulation	CEH min	CVH	Elevation	CCV	R2	p-value
Sim_1	1.06	1.09	0.01	0.00	0.12	0.00
Sim_2	0.21	0.08	0.44	0.38	0.08	0.02
Sim_3	0.06	-0.04	-0.03	0.01	0.00	0.24
Sim_4	0.02	0.02	0.18	0.02	0.03	0.07
Sim_5	-0.05	0.04	-0.40	-0.34	0.04	0.06
Sim_6	0.01	0.01	0.02	-0.02	-0.01	0.60
Sim_7	-0.09	0.02	-0.06	0.17	0.06	0.02
Sim_8	-0.01	0.08	0.01	-0.01	0.00	0.27
Sim_9	-0.03	0.00	0.20	-0.01	0.05	0.03
Sim_10	0.10	0.01	-0.10	0.08	0.04	0.05
Sim_11	-0.02	0.05	0.00	0.02	0.00	0.37
Sim_12	0.15	0.43	0.02	-0.12	0.09	0.06
Sim_13	-0.02	0.01	-0.01	0.06	0.00	0.31
Sim_14	0.08	0.04	0.00	0.01	-0.01	0.60
Sim_15	-0.07	-0.05	-0.06	-0.08	-0.01	0.64
Sim_16	0.07	0.05	0.02	0.09	0.00	0.29
Sim_17	0.00	0.08	0.01	0.04	0.00	0.27
Sim_18	0.02	-0.05	0.01	0.06	-0.01	0.51
Sim_19	-0.11	0.07	-0.02	0.04	0.03	0.07
Sim_20	-0.01	0.01	0.06	0.05	-0.01	0.58
Sim_21	-0.01	0.01	0.05	0.01	0.00	0.38
Sim_22	-0.01	0.05	0.09	0.04	0.00	0.28
Sim_23	0.07	0.07	-0.03	0.01	-0.01	0.53
Sim_24	-0.04	-0.08	0.10	0.05	0.00	0.35
Sim_25	-0.06	0.00	0.00	0.03	0.00	0.43
Sim_26	0.01	0.11	0.01	0.02	0.01	0.21
Sim_27	0.11	0.03	0.08	-0.07	0.02	0.13
Sim_28	-0.03	0.07	0.02	0.06	0.01	0.15
Sim_29	0.05	-0.07	0.11	0.29	0.02	0.12
Sim_30	-0.02	0.04	0.12	0.15	0.04	0.04
Sim_31	0.02	0.05	-0.01	-0.02	-0.01	0.67

Sim_32	0.00	-0.01	0.01	-0.08	0.00	0.27
Sim_33	0.05	-0.02	0.02	-0.09	0.02	0.12
Sim_34	-0.06	0.00	0.04	-0.01	0.00	0.41
Sim_35	0.03	0.07	0.02	0.01	-0.01	0.48
Sim_36	-0.02	0.02	0.12	0.12	0.03	0.07
Sim_37	0.84	0.81	-0.11	0.01	0.07	0.01
Sim_38	0.00	0.02	-0.26	-0.06	0.04	0.04
Sim_39	-0.11	0.00	0.00	0.13	0.03	0.07
Sim_40	0.12	0.12	0.14	0.07	0.01	0.22
Sim_41	0.42	0.25	0.00	0.11	0.04	0.06
Sim_42	-0.60	-0.34	-0.01	-0.04	0.07	0.03
Sim_43	0.05	-0.01	0.03	0.05	-0.01	0.54
Sim_44	-0.07	-0.01	-0.02	-0.01	-0.01	0.51
Sim_45	0.02	-0.54	0.68	0.81	0.14	0.00
Sim_46	-0.16	-0.05	-0.03	0.07	0.01	0.15
Sim_47	-0.02	0.02	0.06	0.02	0.00	0.37
Sim_48	0.11	0.09	-0.02	0.02	-0.01	0.57
Sim_49	0.14	-0.01	0.08	0.01	0.01	0.18
Sim_50	-0.03	0.08	0.12	0.11	0.02	0.09
Sim_51	-0.05	-0.24	0.19	-0.13	0.11	0.01
Sim_52	0.03	0.06	-0.02	-0.02	-0.01	0.65
Sim_53	0.00	0.07	-0.01	0.04	0.00	0.34
Sim_54	0.09	0.15	0.01	0.00	0.00	0.39
Sim_55	0.21	0.18	0.00	0.01	0.00	0.14
Sim_56	0.07	-0.06	0.27	0.44	0.06	0.01
Sim_57	0.62	0.39	0.10	0.00	0.10	0.01
Sim_58	0.05	0.02	-0.11	0.10	0.03	0.06
Sim_59	0.01	0.05	0.05	0.01	-0.01	0.44
Sim_60	0.10	0.02	0.37	0.22	0.05	0.15
Sim_61	-0.02	0.09	0.02	0.00	0.01	0.22
Sim_62	-0.12	-0.08	0.00	0.03	0.00	0.43
Sim_63	0.05	-0.03	-0.02	0.01	0.00	0.34
Sim_64	-0.01	0.01	0.01	0.01	-0.01	0.69
Sim_65	0.48	0.78	0.08	0.07	0.14	0.07
Sim_66	-0.04	0.08	0.01	0.06	0.02	0.14
Sim_67	-0.03	-0.02	0.07	0.04	0.00	0.40
Sim_68	0.51	0.46	0.02	0.00	0.04	0.03
Sim_69	0.01	-0.04	-0.03	-0.08	0.00	0.28
Sim_70	0.25	0.27	-0.09	-0.19	0.03	0.09
Sim_71	0.12	-0.01	0.05	0.06	0.00	0.28
Sim_72	0.01	0.06	0.19	0.07	0.03	0.08

Sim_73	0.31	0.30	0.25	0.13	0.05	0.08
Sim_74	0.06	0.04	0.06	0.08	-0.01	0.54
Sim_75	0.02	-0.02	-0.04	0.09	0.01	0.24
Sim_76	-0.02	-0.03	0.03	-0.01	-0.01	0.57
Sim_77	0.00	-0.03	-0.02	-0.01	-0.01	0.66
Sim_78	0.00	0.00	0.04	-0.01	-0.01	0.44
Sim_79	0.57	0.68	-0.01	0.04	0.06	0.03
Sim_80	1.05	0.90	0.18	0.04	0.09	0.00
Sim_81	0.06	-0.04	-0.01	-0.07	0.01	0.16
Sim_82	-0.06	-0.14	0.02	0.09	-0.01	0.47
Sim_83	0.00	0.02	-0.11	-0.06	0.00	0.32
Sim_84	0.02	-0.03	0.09	0.03	0.00	0.36
Sim_85	0.04	0.06	0.13	0.11	0.01	0.13
Sim_86	0.10	-0.02	0.04	-0.01	0.01	0.19
Sim_87	0.01	-0.04	-0.02	-0.05	0.00	0.37
Sim_88	0.02	0.00	-0.03	0.13	0.02	0.11
Sim_89	-0.03	0.05	0.03	-0.01	0.00	0.39
Sim_90	-0.03	-0.02	0.03	0.03	-0.01	0.71
Sim_91	0.03	0.02	0.26	-0.04	0.07	0.01
Sim_92	-0.09	0.17	-0.03	-0.03	0.05	0.03
Sim_93	0.10	0.25	-0.05	0.04	0.02	0.10
Sim_94	0.02	0.02	-0.06	0.07	0.01	0.19
Sim_95	-0.24	-0.40	-0.01	-0.02	0.05	0.10
Sim_96	-0.03	0.07	-0.19	-0.13	0.00	0.39
Sim_97	0.01	0.10	-0.06	0.03	0.01	0.22
Sim_98	0.07	0.03	-0.06	0.10	0.01	0.15
Sim_99	0.30	0.37	0.01	-0.04	0.02	0.11
Sim_100	-0.05	-0.23	0.26	0.36	0.06	0.01

All species (OLS – sites level)

Simulation	CCV	CEH min	CVH	Elevation	R2	p-value	AIC	BIC
Sim_1	0.38	1.19	1.03	0.37	0.22	0.00	7128.49	7164.01
Sim_2	-0.26	0.11	0.36	0.05	0.10	0.00	7599.06	7634.64
Sim_3	0.67	0.52	0.00	0.39	0.20	0.00	7339.90	7369.60
Sim_4	0.34	0.30	0.28	0.46	0.13	0.00	7577.77	7613.41
Sim_5	0.19	0.24	0.38	0.00	0.10	0.00	7639.80	7669.47
Sim_6	0.17	1.01	0.85	0.51	0.17	0.00	7365.83	7401.40
Sim_7	0.00	-0.90	-0.30	-0.41	0.34	0.00	6768.47	6798.15
Sim_8	-0.27	0.25	0.60	0.09	0.18	0.00	7327.17	7362.73
Sim_9	0.28	0.54	0.23	0.21	0.06	0.00	7758.10	7793.71

Sim_10	0.47	0.64	0.35	0.19	0.16	0.00	7334.88	7370.41
Sim_11	0.55	0.79	0.77	0.28	0.29	0.00	6912.33	6947.89
Sim_12	0.17	0.73	0.50	0.27	0.07	0.00	7680.51	7716.08
Sim_13	0.42	0.67	0.74	0.30	0.23	0.00	7288.39	7324.06
Sim_14	0.05	0.21	0.47	0.00	0.10	0.00	7596.43	7626.07
Sim_15	-0.16	0.23	0.59	-0.08	0.11	0.00	7628.26	7663.89
Sim_16	0.45	0.75	0.57	0.44	0.13	0.00	7575.20	7610.83
Sim_17	-0.39	-0.84	-0.18	-0.42	0.22	0.00	7226.00	7261.60
Sim_18	0.61	1.82	1.24	0.81	0.40	0.00	6538.14	6573.76
Sim_19	0.42	0.70	0.51	0.09	0.18	0.00	7134.74	7170.14
Sim_20	0.11	0.70	0.88	0.34	0.20	0.00	7369.02	7404.67
Sim_21	0.13	0.71	0.72	0.53	0.21	0.00	7304.67	7340.29
Sim_22	0.49	0.23	0.23	0.29	0.17	0.00	7406.99	7442.60
Sim_23	0.12	0.24	0.29	0.17	0.03	0.00	7870.40	7906.04
Sim_24	0.35	0.04	0.02	-0.02	0.13	0.00	7610.08	7633.85
Sim_25	0.10	0.81	0.96	0.29	0.18	0.00	7406.59	7442.23
Sim_26	0.01	1.26	1.40	0.43	0.37	0.00	6624.68	6654.34
Sim_27	0.13	1.00	0.55	0.34	0.18	0.00	7371.69	7407.29
Sim_28	0.05	0.48	0.64	0.19	0.10	0.00	7616.93	7652.53
Sim_29	0.19	0.65	0.77	0.22	0.14	0.00	7602.74	7638.41
Sim_30	-0.02	0.24	0.60	0.10	0.17	0.00	7422.38	7452.06
Sim_31	-0.24	0.15	0.63	0.01	0.21	0.00	7350.31	7380.04
Sim_32	0.44	0.83	0.24	0.36	0.17	0.00	7351.49	7387.06
Sim_33	0.13	0.08	0.34	0.15	0.13	0.00	7566.65	7602.28
Sim_34	-0.24	-0.20	0.59	-0.23	0.42	0.00	6235.18	6270.61
Sim_35	0.19	0.55	0.55	0.30	0.08	0.00	7494.01	7529.46
Sim_36	0.50	0.27	0.59	0.09	0.48	0.00	6019.67	6055.22
Sim_37	0.11	1.04	0.88	0.47	0.17	0.00	7383.86	7419.44
Sim_38	0.14	-0.01	0.30	0.01	0.15	0.00	7538.01	7561.78
Sim_39	0.16	-0.80	-0.14	-0.32	0.54	0.00	5851.88	5887.55
Sim_40	0.13	1.08	0.68	0.62	0.24	0.00	7164.07	7199.68
Sim_41	0.33	0.19	0.44	0.16	0.23	0.00	7208.52	7244.13
Sim_42	0.37	0.00	-0.21	0.32	0.06	0.00	7851.11	7880.84
Sim_43	0.52	0.76	0.63	0.46	0.18	0.00	7366.83	7402.43
Sim_44	-0.04	-0.80	-0.25	-0.29	0.25	0.00	7185.07	7220.74
Sim_45	0.60	1.11	0.45	0.51	0.22	0.00	6995.95	7031.36
Sim_46	0.17	-0.53	-0.08	-0.13	0.28	0.00	7039.41	7075.03
Sim_47	0.20	1.33	1.05	0.57	0.24	0.00	7196.30	7231.92
Sim_48	0.05	0.35	0.55	0.36	0.19	0.00	7425.50	7461.17
Sim_49	0.30	1.04	0.74	0.47	0.13	0.00	7516.18	7551.76
Sim_50	0.24	0.89	0.59	0.38	0.09	0.00	7669.10	7704.72

Sim_51	0.65	1.22	0.60	0.49	0.28	0.00	7039.25	7074.89
Sim_52	0.36	0.92	0.67	0.50	0.12	0.00	7640.27	7675.94
Sim_53	0.03	0.46	1.03	0.03	0.43	0.00	6366.38	6401.99
Sim_54	0.00	0.23	0.56	0.01	0.13	0.00	7358.03	7381.68
Sim_55	-0.16	-0.35	0.30	-0.14	0.29	0.00	6947.79	6983.39
Sim_56	-0.44	-0.67	0.18	-0.47	0.35	0.00	6571.27	6606.75
Sim_57	0.59	1.21	0.51	0.68	0.27	0.00	7076.22	7111.85
Sim_58	0.34	0.70	0.12	0.35	0.17	0.00	7490.41	7526.07
Sim_59	0.48	0.39	0.22	0.33	0.10	0.00	7698.02	7733.69
Sim_60	0.00	0.69	0.67	0.30	0.10	0.00	7599.53	7629.18
Sim_61	0.23	0.78	0.70	0.61	0.20	0.00	7317.07	7352.69
Sim_62	0.00	-0.60	0.02	-0.16	0.32	0.00	6778.88	6802.59
Sim_63	0.34	-0.52	-0.25	-0.19	0.31	0.00	6907.08	6942.69
Sim_64	0.39	0.95	0.62	0.42	0.12	0.00	7652.32	7687.98
Sim_65	-0.31	0.02	0.68	-0.07	0.35	0.00	6481.57	6511.05
Sim_66	0.24	0.19	0.10	0.22	0.02	0.00	7886.03	7921.66
Sim_67	0.28	1.54	1.24	0.49	0.28	0.00	7011.00	7046.58
Sim_68	0.28	0.27	0.48	0.02	0.20	0.00	7354.48	7384.18
Sim_69	0.18	0.76	0.62	0.40	0.09	0.00	7700.77	7736.41
Sim_70	0.24	0.63	0.65	0.15	0.11	0.00	7556.81	7592.37
Sim_71	0.60	1.18	0.77	0.44	0.24	0.00	7080.14	7115.68
Sim_72	0.60	0.33	0.41	0.43	0.32	0.00	6868.47	6904.10
Sim_73	0.13	0.00	0.49	0.06	0.32	0.00	6872.02	6901.70
Sim_74	0.04	0.27	0.72	-0.09	0.25	0.00	7137.33	7172.96
Sim_75	-0.02	0.24	0.29	0.28	0.08	0.00	7734.45	7764.15
Sim_76	0.57	1.38	0.70	0.56	0.30	0.00	6833.63	6869.16
Sim_77	0.31	0.04	0.26	-0.12	0.25	0.00	7166.54	7202.18
Sim_78	0.44	0.10	0.20	0.00	0.25	0.00	7172.20	7201.89
Sim_79	0.26	1.39	1.19	0.46	0.23	0.00	7183.45	7219.04
Sim_80	0.70	0.68	0.40	0.31	0.27	0.00	6971.89	7007.44
Sim_81	-0.15	0.24	0.71	0.02	0.21	0.00	7227.58	7263.16
Sim_82	0.18	-0.54	-0.07	-0.20	0.32	0.00	6897.95	6933.58
Sim_83	0.10	0.07	0.30	0.00	0.08	0.00	7714.92	7744.61
Sim_84	0.55	0.36	0.00	0.49	0.11	0.00	7657.21	7686.92
Sim_85	0.36	0.48	0.35	0.25	0.07	0.00	7707.00	7742.59
Sim_86	0.44	1.33	0.75	0.56	0.24	0.00	7238.25	7273.91
Sim_87	0.24	0.12	0.29	0.09	0.12	0.00	7653.98	7689.64
Sim_88	0.58	0.94	0.61	0.67	0.17	0.00	7318.51	7354.05
Sim_89	0.29	0.01	0.30	0.08	0.22	0.00	7224.12	7253.79
Sim_90	0.14	0.22	0.38	-0.01	0.08	0.00	7607.56	7637.17
Sim_91	-0.20	0.42	0.67	0.21	0.18	0.00	7296.82	7332.37

Sim_92	-0.11	0.37	0.55	0.17	0.10	0.00	7698.20	7733.85
Sim_93	-0.23	0.56	0.87	0.01	0.16	0.00	7498.87	7528.57
Sim_94	0.47	0.87	0.46	0.44	0.12	0.00	7557.48	7593.08
Sim_95	0.01	0.36	0.02	0.23	0.11	0.00	7652.73	7676.49
Sim_96	0.02	0.30	0.39	0.05	0.03	0.00	7862.35	7897.96
Sim_97	0.18	0.52	0.69	-0.06	0.18	0.00	7454.89	7490.56
Sim_98	-0.31	-0.15	0.45	0.00	0.30	0.00	6865.72	6895.35
Sim_99	0.32	1.13	1.03	0.45	0.20	0.00	7317.14	7352.76
Sim_100	-0.06	-0.22	0.10	-0.15	0.07	0.00	7716.70	7752.30

Tonini (OLS – sites level)

Simulation	CCV	CEH min	CVH	Elevation	R2	p-value	AIC	BIC
Sim_1	0.38	1.22	1.06	0.38	0.23	0.00	7097.04	7132.56
Sim_2	-0.23	0.19	0.42	0.11	0.11	0.00	7564.03	7599.61
Sim_3	0.65	0.51	0.00	0.38	0.19	0.00	7371.11	7400.81
Sim_4	0.38	0.28	0.30	0.47	0.16	0.00	7476.71	7512.35
Sim_5	0.20	0.24	0.40	0.00	0.11	0.00	7594.72	7624.39
Sim_6	0.16	0.99	0.84	0.49	0.16	0.00	7378.05	7413.61
Sim_7	-0.01	-0.95	-0.36	-0.42	0.34	0.00	6791.55	6821.22
Sim_8	-0.27	0.31	0.67	0.07	0.18	0.00	7315.41	7350.97
Sim_9	0.28	0.52	0.23	0.19	0.06	0.00	7763.88	7799.50
Sim_10	0.42	0.68	0.45	0.16	0.15	0.00	7360.22	7395.74
Sim_11	0.58	0.75	0.72	0.29	0.30	0.00	6880.21	6915.76
Sim_12	0.19	0.70	0.47	0.27	0.06	0.00	7694.03	7729.59
Sim_13	0.43	0.67	0.75	0.31	0.24	0.00	7235.62	7271.28
Sim_14	0.03	0.15	0.44	-0.01	0.10	0.00	7566.52	7596.15
Sim_15	-0.07	0.31	0.62	-0.04	0.11	0.00	7638.84	7674.46
Sim_16	0.48	0.78	0.58	0.45	0.14	0.00	7543.84	7579.47
Sim_17	-0.35	-0.70	-0.05	-0.37	0.21	0.00	7253.30	7288.90
Sim_18	0.58	1.78	1.20	0.78	0.38	0.00	6631.77	6667.39
Sim_19	0.46	0.78	0.53	0.16	0.18	0.00	7136.80	7172.20
Sim_20	0.11	0.73	0.85	0.36	0.17	0.00	7459.80	7495.45
Sim_21	0.19	0.72	0.69	0.58	0.21	0.00	7293.67	7329.29
Sim_22	0.51	0.25	0.25	0.32	0.18	0.00	7368.64	7404.25
Sim_23	0.04	0.36	0.46	0.14	0.05	0.00	7809.72	7845.34
Sim_24	0.37	0.09	0.01	0.00	0.13	0.00	7617.18	7640.96
Sim_25	0.08	0.86	1.00	0.28	0.18	0.00	7398.71	7434.35
Sim_26	0.04	1.28	1.42	0.46	0.38	0.00	6577.44	6613.03
Sim_27	0.17	1.00	0.51	0.34	0.18	0.00	7355.95	7391.55
Sim_28	-0.06	0.30	0.52	0.11	0.08	0.00	7674.47	7710.07

Sim_29	0.21	0.60	0.72	0.21	0.14	0.00	7609.40	7645.08
Sim_30	-0.03	0.25	0.63	0.10	0.18	0.00	7376.74	7412.36
Sim_31	-0.22	0.17	0.64	0.01	0.21	0.00	7371.30	7401.03
Sim_32	0.43	0.89	0.31	0.35	0.18	0.00	7338.88	7374.46
Sim_33	0.18	0.12	0.35	0.17	0.13	0.00	7567.85	7603.49
Sim_34	-0.21	-0.21	0.57	-0.23	0.41	0.00	6273.22	6308.65
Sim_35	0.25	0.60	0.48	0.35	0.07	0.00	7492.92	7528.34
Sim_36	0.53	0.28	0.57	0.10	0.49	0.00	5989.63	6025.16
Sim_37	0.01	0.87	0.78	0.37	0.13	0.00	7429.63	7459.23
Sim_38	0.15	-0.01	0.29	0.00	0.15	0.00	7526.81	7550.58
Sim_39	0.10	-0.84	-0.14	-0.41	0.54	0.00	5782.80	5818.46
Sim_40	0.02	0.91	0.60	0.55	0.23	0.00	7200.73	7236.34
Sim_41	0.35	0.21	0.46	0.17	0.25	0.00	7128.99	7164.60
Sim_42	0.39	-0.01	-0.29	0.33	0.07	0.00	7825.37	7855.10
Sim_43	0.52	0.68	0.60	0.47	0.19	0.00	7316.79	7352.39
Sim_44	-0.01	-0.80	-0.25	-0.28	0.27	0.00	7128.85	7158.58
Sim_45	0.63	1.02	0.39	0.52	0.20	0.00	7033.52	7068.89
Sim_46	0.13	-0.64	-0.14	-0.16	0.30	0.00	6952.56	6988.18
Sim_47	0.22	1.30	1.04	0.60	0.24	0.00	7173.54	7209.16
Sim_48	0.03	0.40	0.57	0.37	0.18	0.00	7453.21	7488.89
Sim_49	0.33	1.10	0.79	0.50	0.14	0.00	7460.95	7496.53
Sim_50	0.28	0.98	0.57	0.43	0.12	0.00	7574.02	7609.64
Sim_51	0.67	1.24	0.63	0.51	0.29	0.00	6999.56	7035.21
Sim_52	0.37	0.91	0.67	0.47	0.12	0.00	7654.06	7689.73
Sim_53	0.00	0.35	0.96	0.00	0.42	0.00	6380.81	6404.55
Sim_54	0.00	0.21	0.55	0.00	0.14	0.00	7345.78	7369.42
Sim_55	-0.21	-0.38	0.33	-0.16	0.33	0.00	6798.61	6834.21
Sim_56	-0.45	-0.65	0.18	-0.45	0.34	0.00	6623.41	6658.90
Sim_57	0.60	1.14	0.46	0.67	0.25	0.00	7145.94	7181.57
Sim_58	0.36	0.67	0.09	0.34	0.17	0.00	7484.89	7520.55
Sim_59	0.50	0.33	0.19	0.32	0.12	0.00	7652.33	7688.00
Sim_60	-0.14	0.36	0.51	0.12	0.07	0.00	7633.26	7668.80
Sim_61	0.22	0.87	0.79	0.62	0.22	0.00	7250.40	7286.02
Sim_62	0.00	-0.58	0.02	-0.18	0.30	0.00	6861.27	6884.98
Sim_63	0.36	-0.42	-0.21	-0.13	0.27	0.00	7060.63	7096.24
Sim_64	0.35	0.67	0.46	0.27	0.08	0.00	7767.23	7802.89
Sim_65	-0.36	0.02	0.67	-0.11	0.33	0.00	6461.13	6490.53
Sim_66	0.28	0.28	0.11	0.25	0.03	0.00	7871.25	7906.88
Sim_67	0.30	1.55	1.26	0.52	0.28	0.00	6978.84	7014.43
Sim_68	0.25	0.32	0.52	0.01	0.18	0.00	7390.53	7420.23
Sim_69	0.20	0.77	0.64	0.41	0.10	0.00	7683.89	7719.53

Sim_70	0.29	0.74	0.72	0.22	0.12	0.00	7503.53	7539.09
Sim_71	0.60	1.11	0.71	0.41	0.23	0.00	7116.88	7152.42
Sim_72	0.62	0.29	0.38	0.40	0.35	0.00	6778.71	6814.34
Sim_73	0.10	-0.10	0.41	0.02	0.30	0.00	6938.34	6968.03
Sim_74	0.11	0.31	0.71	-0.03	0.25	0.00	7145.06	7180.70
Sim_75	-0.03	0.28	0.31	0.29	0.09	0.00	7714.20	7749.83
Sim_76	0.52	1.27	0.61	0.49	0.26	0.00	6922.98	6958.45
Sim_77	0.32	0.03	0.27	-0.10	0.26	0.00	7126.88	7156.57
Sim_78	0.43	0.11	0.21	0.00	0.24	0.00	7212.12	7241.82
Sim_79	0.19	1.45	1.27	0.40	0.25	0.00	7045.76	7081.29
Sim_80	0.69	0.61	0.37	0.27	0.28	0.00	6927.04	6962.58
Sim_81	-0.16	0.18	0.61	-0.01	0.16	0.00	7401.75	7431.40
Sim_82	0.21	-0.47	-0.01	-0.17	0.33	0.00	6838.84	6868.53
Sim_83	0.17	0.05	0.28	0.02	0.11	0.00	7608.24	7631.99
Sim_84	0.58	0.40	-0.03	0.54	0.12	0.00	7607.04	7636.75
Sim_85	0.33	0.38	0.31	0.20	0.07	0.00	7710.89	7746.48
Sim_86	0.47	1.32	0.76	0.56	0.24	0.00	7248.53	7284.20
Sim_87	0.22	0.11	0.34	0.07	0.14	0.00	7564.58	7600.25
Sim_88	0.58	0.94	0.61	0.67	0.17	0.00	7313.38	7348.92
Sim_89	0.24	-0.09	0.25	0.00	0.23	0.00	7192.83	7222.50
Sim_90	0.06	0.01	0.25	-0.10	0.08	0.00	7597.92	7627.53
Sim_91	-0.19	0.47	0.70	0.23	0.19	0.00	7271.49	7307.03
Sim_92	-0.12	0.39	0.58	0.17	0.11	0.00	7679.48	7715.13
Sim_93	-0.22	0.57	0.88	0.05	0.17	0.00	7463.65	7499.30
Sim_94	0.42	0.89	0.52	0.43	0.11	0.00	7594.95	7630.55
Sim_95	0.00	0.39	0.01	0.27	0.13	0.00	7577.45	7601.22
Sim_96	0.05	0.56	0.53	0.18	0.05	0.00	7815.50	7851.12
Sim_97	0.20	0.66	0.76	0.00	0.17	0.00	7478.03	7507.76
Sim_98	-0.33	-0.13	0.46	0.00	0.30	0.00	6868.81	6898.43
Sim_99	0.35	1.11	1.01	0.45	0.20	0.00	7303.63	7339.25
Sim_100	-0.09	-0.27	0.08	-0.21	0.08	0.00	7698.96	7734.56

Leache (OLS – sites level)

Simulation	CCV	CEH min	CVH	Elevation	R2	p-value	AIC	BIC
Sim_1	0.33	1.07	0.95	0.39	0.19	0.00	7133.34	7168.77
Sim_2	-0.37	0.09	0.41	0.00	0.15	0.00	7424.23	7453.85
Sim_3	0.75	0.62	0.13	0.36	0.27	0.00	7079.80	7115.43
Sim_4	0.33	0.35	0.34	0.47	0.13	0.00	7565.28	7600.91
Sim_5	0.23	0.11	0.24	-0.02	0.10	0.00	7620.45	7650.12
Sim_6	0.15	1.14	0.98	0.50	0.19	0.00	7086.37	7121.77

Sim_7	-0.03	-0.95	-0.30	-0.33	0.36	0.00	6690.62	6726.24
Sim_8	-0.15	0.38	0.47	0.22	0.11	0.00	7379.75	7415.18
Sim_9	0.32	0.50	0.16	0.18	0.08	0.00	7711.83	7747.45
Sim_10	0.42	0.74	0.55	0.20	0.15	0.00	7345.58	7381.09
Sim_11	0.53	0.75	0.88	0.28	0.38	0.00	6468.34	6503.82
Sim_12	0.24	0.78	0.49	0.28	0.08	0.00	7569.12	7604.63
Sim_13	0.42	0.80	0.77	0.43	0.19	0.00	7407.48	7443.15
Sim_14	0.00	0.24	0.55	0.00	0.12	0.00	7510.83	7534.53
Sim_15	0.03	0.42	0.76	0.00	0.18	0.00	7406.26	7435.95
Sim_16	0.41	0.71	0.56	0.46	0.12	0.00	7604.86	7640.48
Sim_17	-0.38	-0.61	0.00	-0.28	0.20	0.00	7288.63	7318.30
Sim_18	0.58	1.77	1.19	0.78	0.37	0.00	6646.18	6681.80
Sim_19	0.50	0.79	0.45	0.20	0.19	0.00	7077.26	7112.64
Sim_20	-0.10	0.63	0.89	0.24	0.20	0.00	7341.62	7377.27
Sim_21	0.10	0.72	0.78	0.49	0.21	0.00	7281.81	7317.43
Sim_22	0.61	0.28	0.15	0.38	0.18	0.00	7368.19	7403.80
Sim_23	0.15	0.46	0.36	0.16	0.03	0.00	7801.10	7836.67
Sim_24	0.36	0.22	0.11	-0.03	0.14	0.00	7494.10	7529.70
Sim_25	0.11	1.04	1.15	0.32	0.23	0.00	7243.19	7278.83
Sim_26	0.13	1.29	1.42	0.48	0.39	0.00	6483.75	6519.30
Sim_27	0.32	0.75	0.29	0.25	0.13	0.00	7476.48	7512.03
Sim_28	-0.03	0.25	0.51	0.13	0.11	0.00	7582.24	7617.82
Sim_29	0.15	0.58	0.78	0.21	0.16	0.00	7520.76	7556.44
Sim_30	0.00	0.29	0.66	0.08	0.18	0.00	7330.82	7360.46
Sim_31	-0.21	0.31	0.79	0.00	0.22	0.00	7332.81	7362.54
Sim_32	0.50	0.81	0.18	0.36	0.20	0.00	7252.50	7288.08
Sim_33	0.27	0.21	0.46	0.17	0.20	0.00	7305.97	7341.58
Sim_34	-0.21	-0.01	0.71	-0.22	0.37	0.00	6388.70	6418.19
Sim_35	0.17	0.70	0.44	0.35	0.08	0.00	7323.11	7358.43
Sim_36	0.43	0.15	0.50	0.01	0.44	0.00	6024.74	6054.17
Sim_37	0.20	1.05	0.86	0.51	0.16	0.00	7388.04	7423.61
Sim_38	0.19	-0.01	0.21	0.01	0.11	0.00	7611.76	7635.51
Sim_39	0.10	-0.71	-0.04	-0.36	0.49	0.00	6042.83	6078.44
Sim_40	0.02	0.81	0.59	0.49	0.17	0.00	7297.38	7326.98
Sim_41	0.11	-0.31	0.20	-0.04	0.30	0.00	6944.49	6980.09
Sim_42	0.33	0.26	-0.03	0.32	0.04	0.00	7892.45	7922.18
Sim_43	0.48	0.66	0.56	0.43	0.16	0.00	7434.05	7469.64
Sim_44	-0.01	-0.90	-0.33	-0.26	0.30	0.00	7012.43	7042.16
Sim_45	0.56	1.07	0.40	0.54	0.19	0.00	6973.16	7008.46
Sim_46	0.15	-0.68	-0.21	-0.17	0.29	0.00	6984.06	7019.68
Sim_47	0.23	1.16	0.86	0.58	0.20	0.00	7329.93	7365.55

Sim_48	0.01	0.24	0.47	0.34	0.20	0.00	7403.73	7433.46
Sim_49	0.30	1.29	0.92	0.55	0.20	0.00	7287.27	7322.85
Sim_50	0.32	1.27	0.78	0.62	0.20	0.00	7145.99	7181.46
Sim_51	0.65	1.28	0.68	0.53	0.28	0.00	7038.32	7073.96
Sim_52	0.31	0.97	0.79	0.46	0.13	0.00	7572.68	7608.32
Sim_53	-0.01	0.29	0.89	-0.06	0.39	0.00	6319.53	6349.03
Sim_54	0.02	0.28	0.61	0.03	0.15	0.00	7313.89	7337.54
Sim_55	-0.27	-0.52	0.21	-0.22	0.32	0.00	6848.12	6883.71
Sim_56	-0.55	-0.70	0.18	-0.55	0.36	0.00	6540.76	6576.23
Sim_57	0.52	0.84	0.37	0.57	0.14	0.00	7539.36	7574.99
Sim_58	0.32	0.80	0.23	0.34	0.17	0.00	7484.39	7520.06
Sim_59	0.49	0.19	0.16	0.30	0.15	0.00	7538.19	7573.85
Sim_60	-0.14	0.41	0.59	0.13	0.09	0.00	7547.69	7583.21
Sim_61	0.25	0.93	0.76	0.66	0.21	0.00	7269.92	7305.54
Sim_62	-0.08	-0.81	-0.19	-0.31	0.29	0.00	6916.01	6951.57
Sim_63	0.38	-0.46	-0.24	0.00	0.26	0.00	7101.40	7131.08
Sim_64	0.35	0.73	0.54	0.25	0.10	0.00	7711.02	7746.68
Sim_65	-0.31	0.10	0.75	0.00	0.38	0.00	6315.03	6344.49
Sim_66	0.24	0.65	0.57	0.27	0.07	0.00	7750.25	7785.88
Sim_67	0.24	1.34	1.14	0.45	0.22	0.00	7177.75	7213.31
Sim_68	0.17	-0.04	0.23	-0.16	0.17	0.00	7447.89	7483.52
Sim_69	0.16	0.81	0.72	0.43	0.12	0.00	7615.37	7651.00
Sim_70	0.32	0.65	0.55	0.20	0.10	0.00	7583.13	7618.69
Sim_71	0.47	1.02	0.75	0.37	0.17	0.00	7276.11	7311.61
Sim_72	0.59	0.19	0.31	0.30	0.33	0.00	6808.05	6843.63
Sim_73	0.07	-0.13	0.45	-0.01	0.36	0.00	6715.17	6744.85
Sim_74	0.10	0.28	0.63	-0.07	0.21	0.00	7122.58	7158.06
Sim_75	0.09	0.39	0.39	0.35	0.08	0.00	7733.91	7769.54
Sim_76	0.62	1.27	0.57	0.52	0.30	0.00	6793.51	6828.99
Sim_77	0.26	0.04	0.22	-0.10	0.18	0.00	7413.70	7449.33
Sim_78	0.46	-0.15	0.01	0.00	0.29	0.00	7014.85	7038.61
Sim_79	0.23	1.52	1.24	0.45	0.26	0.00	6972.80	7008.31
Sim_80	0.65	0.49	0.29	0.21	0.26	0.00	6961.88	6997.37
Sim_81	-0.34	-0.01	0.51	-0.14	0.18	0.00	7351.75	7381.41
Sim_82	0.17	-0.46	0.00	-0.23	0.30	0.00	6952.48	6982.17
Sim_83	0.14	0.03	0.32	0.00	0.14	0.00	7512.57	7542.26
Sim_84	0.56	0.59	0.19	0.61	0.13	0.00	7581.12	7616.76
Sim_85	0.44	0.45	0.41	0.25	0.14	0.00	7498.37	7533.96
Sim_86	0.42	1.27	0.80	0.49	0.21	0.00	7274.48	7310.07
Sim_87	0.24	-0.07	0.16	0.01	0.15	0.00	7557.41	7587.13
Sim_88	0.62	0.87	0.59	0.61	0.18	0.00	7283.08	7318.62

Sim_89	0.25	-0.05	0.29	0.02	0.24	0.00	7165.71	7195.38
Sim_90	0.12	0.04	0.17	-0.08	0.06	0.00	7512.43	7547.83
Sim_91	-0.17	0.42	0.67	0.24	0.19	0.00	7215.53	7251.03
Sim_92	-0.20	0.25	0.57	0.05	0.12	0.00	7614.12	7649.76
Sim_93	-0.21	0.58	0.76	0.03	0.11	0.00	7662.34	7697.98
Sim_94	0.41	0.88	0.55	0.42	0.10	0.00	7597.21	7632.79
Sim_95	0.00	0.40	0.06	0.32	0.13	0.00	7570.84	7600.54
Sim_96	0.09	0.32	0.24	0.08	0.01	0.00	7904.37	7939.98
Sim_97	0.21	0.66	0.79	0.06	0.17	0.00	7472.13	7507.80
Sim_98	-0.32	-0.22	0.41	-0.01	0.32	0.00	6777.14	6806.76
Sim_99	0.37	0.95	0.92	0.45	0.20	0.00	7308.23	7343.85
Sim_100	-0.06	-0.39	0.02	-0.21	0.12	0.00	7555.82	7585.49

All species (SARs)

Simulation	CCV	Elevation	CEH min	CVH	R2	p-value	AIC	Scheme	Model
Sim_1	0.15	0.11	0.40	0.35	0.60	0.00	5317.63	W	error
Sim_2	0.34	0.12	0.39	0.18	0.43	0.00	6276.78	W	error
Sim_3	0.00	-0.13	-0.20	0.08	0.33	0.00	6827.19	W	error
Sim_4	0.25	0.03	0.18	0.30	0.60	0.00	5362.19	W	error
Sim_5	0.01	0.06	0.26	0.10	0.62	0.00	5199.25	W	error
Sim_6	0.32	0.17	0.40	0.51	0.47	0.00	6216.39	W	error
Sim_7	0.10	0.04	0.10	0.30	0.43	0.00	6300.59	W	error
Sim_8	-0.09	-0.04	0.12	0.41	0.35	0.00	6751.04	W	error
Sim_9	0.33	0.30	0.56	0.45	0.35	0.00	6740.15	W	error
Sim_10	-0.29	-0.35	-0.78	-0.19	0.48	0.00	6082.51	W	error
Sim_11	0.35	0.53	1.22	0.78	0.67	0.00	4821.24	W	error
Sim_12	0.16	-0.07	0.19	0.12	0.63	0.00	4987.48	W	error
Sim_13	-0.09	0.06	0.00	0.09	0.51	0.00	5929.97	W	error
Sim_14	0.06	0.23	0.40	0.56	0.54	0.00	5787.43	W	error
Sim_15	0.00	0.35	0.46	0.52	0.55	0.00	5701.89	W	error
Sim_16	0.35	0.18	0.19	0.24	0.49	0.00	6081.15	S	error
Sim_17	0.06	0.03	-0.02	0.04	0.48	0.00	6125.16	W	error
Sim_18	0.23	-0.10	-0.07	-0.02	0.52	0.00	5962.31	W	error
Sim_19	0.06	0.14	0.26	0.41	0.50	0.00	6057.54	W	error
Sim_20	-0.05	0.20	0.38	0.46	0.65	0.00	4959.67	W	error
Sim_21	0.12	0.30	0.77	0.33	0.48	0.00	6104.82	W	error
Sim_22	-0.01	0.02	-0.12	0.01	0.52	0.00	5901.91	W	error
Sim_23	0.14	0.16	0.42	0.55	0.49	0.00	6130.10	W	error
Sim_24	0.35	0.11	0.33	0.02	0.53	0.00	5848.95	W	error
Sim_25	-0.03	0.06	0.09	0.42	0.46	0.00	6210.18	W	error

Sim_26	-0.17	0.04	0.11	0.53	0.48	0.00	6166.55	W	error
Sim_27	0.19	0.18	0.55	0.13	0.53	0.00	5779.40	W	error
Sim_28	0.08	0.02	-0.12	0.14	0.48	0.00	6116.62	W	error
Sim_29	-0.08	-0.18	-0.44	0.11	0.72	0.00	4299.72	W	error
Sim_30	0.02	0.02	0.15	0.19	0.61	0.00	5139.86	W	error
Sim_31	0.20	-0.13	-0.31	0.10	0.73	0.00	4285.86	W	error
Sim_32	0.02	0.33	0.69	0.57	0.52	0.00	5874.66	W	error
Sim_33	0.09	-0.06	-0.24	0.08	0.55	0.00	5764.85	W	error
Sim_34	0.13	-0.35	-0.83	-0.17	0.67	0.00	4848.39	W	error
Sim_35	0.16	0.24	0.16	0.23	0.48	0.00	6112.67	W	error
Sim_36	0.06	0.47	0.83	0.46	0.54	0.00	5784.77	W	error
Sim_37	0.22	0.01	-0.12	0.17	0.50	0.00	6032.20	W	error
Sim_38	0.18	0.11	0.08	-0.03	0.47	0.00	6212.73	W	error
Sim_39	0.31	0.27	0.45	0.44	0.50	0.00	6008.83	W	error
Sim_40	-0.03	-0.30	-0.82	-0.29	0.50	0.00	6053.50	W	error
Sim_41	0.16	0.17	0.47	0.13	0.63	0.00	5019.09	W	error
Sim_42	0.13	-0.18	-0.59	-0.13	0.53	0.00	5828.88	W	error
Sim_43	0.09	0.36	0.79	0.54	0.57	0.00	5610.11	W	error
Sim_44	0.02	0.28	0.23	0.44	0.45	0.00	6323.91	W	error
Sim_45	0.16	0.24	0.56	0.31	0.55	0.00	5691.67	W	error
Sim_46	0.12	-0.07	0.08	0.24	0.43	0.00	6372.90	W	error
Sim_47	0.07	0.14	0.44	0.21	0.56	0.00	5671.40	W	error
Sim_48	0.15	0.11	0.33	0.06	0.69	0.00	4734.03	W	error
Sim_49	0.23	0.34	0.69	0.53	0.41	0.00	6549.41	W	error
Sim_50	-0.03	-0.15	-0.34	0.13	0.70	0.00	4566.74	W	error
Sim_51	0.03	-0.05	-0.07	0.17	0.49	0.00	5920.89	W	error
Sim_52	-0.10	-0.14	-0.43	0.15	0.62	0.00	5255.39	W	error
Sim_53	-0.22	-0.30	-0.67	-0.01	0.59	0.00	5327.33	W	error
Sim_54	0.35	0.45	0.87	0.33	0.55	0.00	5755.02	W	error
Sim_55	0.04	0.04	0.28	-0.05	0.62	0.00	5261.07	W	error
Sim_56	0.28	0.13	0.14	0.09	0.46	0.00	6259.43	W	error
Sim_57	0.10	0.38	0.72	0.57	0.50	0.00	5933.78	W	error
Sim_58	-0.02	0.13	0.31	0.22	0.57	0.00	5555.69	W	error
Sim_59	0.09	0.38	0.50	0.49	0.62	0.00	5272.39	W	error
Sim_60	-0.03	-0.20	-0.59	0.01	0.56	0.00	5599.95	W	error
Sim_61	0.27	-0.24	-0.58	-0.27	0.53	0.00	5844.96	W	error
Sim_62	0.26	0.29	0.72	0.46	0.37	0.00	6689.82	W	error
Sim_63	-0.09	-0.06	-0.27	0.12	0.78	0.00	3615.16	W	error
Sim_64	0.17	0.13	0.09	-0.01	0.29	0.00	7008.49	S	error
Sim_65	0.23	0.43	1.08	0.80	0.55	0.00	5698.23	W	error
Sim_66	0.22	-0.03	-0.07	0.15	0.49	0.00	6058.77	W	error

Sim_67	-0.02	0.07	0.15	0.09	0.57	0.00	5600.66	W	error
Sim_68	-0.03	-0.39	-0.80	-0.21	0.59	0.00	5457.58	W	error
Sim_69	0.19	0.10	0.28	0.32	0.37	0.00	6602.28	W	error
Sim_70	0.36	0.24	0.67	0.40	0.55	0.00	5632.47	W	error
Sim_71	0.19	0.03	-0.12	0.14	0.61	0.00	5338.57	W	error
Sim_72	0.09	0.01	-0.11	0.40	0.51	0.00	5978.49	W	error
Sim_73	0.10	-0.06	0.02	0.41	0.50	0.00	6036.27	W	error
Sim_74	-0.07	0.20	0.21	0.28	0.33	0.00	6853.71	W	error
Sim_75	0.20	0.25	0.66	0.21	0.68	0.00	4696.81	W	error
Sim_76	0.25	-0.15	-0.14	0.11	0.48	0.00	6154.80	S	error
Sim_77	0.21	-0.11	-0.23	-0.02	0.56	0.00	5703.59	W	error
Sim_78	0.14	0.28	0.65	0.49	0.68	0.00	4712.77	W	error
Sim_79	-0.17	0.07	0.07	0.34	0.56	0.00	5568.20	W	error
Sim_80	0.24	-0.03	0.08	0.08	0.63	0.00	5085.18	W	error
Sim_81	-0.07	0.07	0.03	0.40	0.43	0.00	6360.04	W	error
Sim_82	0.14	-0.22	-0.58	-0.10	0.55	0.00	5705.16	W	error
Sim_83	0.09	-0.01	0.02	0.23	0.24	0.00	7181.86	W	error
Sim_84	0.22	0.19	0.25	0.07	0.54	0.00	5797.85	W	error
Sim_85	0.21	0.08	0.11	0.08	0.34	0.00	6766.82	W	error
Sim_86	0.22	0.32	0.78	0.33	0.50	0.00	6037.10	W	error
Sim_87	0.18	0.03	0.01	0.20	0.36	0.00	6769.09	W	error
Sim_88	0.07	0.05	0.07	0.09	0.60	0.00	5321.21	W	error
Sim_89	0.12	-0.06	-0.23	0.14	0.59	0.00	5471.47	W	error
Sim_90	0.24	0.17	0.45	0.17	0.26	0.00	7103.57	W	error
Sim_91	0.08	-0.08	-0.02	0.12	0.48	0.00	6044.30	W	error
Sim_92	-0.07	0.16	0.19	0.32	0.54	0.00	5726.27	W	error
Sim_93	-0.12	0.12	0.25	0.43	0.39	0.00	6625.01	W	error
Sim_94	-0.08	0.09	0.27	0.45	0.51	0.00	5959.01	W	error
Sim_95	0.24	0.24	0.56	0.28	0.49	0.00	6061.85	W	error
Sim_96	0.08	0.26	0.50	0.11	0.50	0.00	6054.21	W	error
Sim_97	0.06	0.06	0.24	0.29	0.32	0.00	6882.49	W	error
Sim_98	0.09	-0.13	-0.07	0.13	0.59	0.00	5510.82	W	error
Sim_99	-0.20	-0.01	-0.36	0.12	0.56	0.00	5610.17	W	error
Sim_100	0.25	0.32	0.72	0.64	0.50	0.00	6009.49	W	error

Tonini (SARs)

Simulation	CCV	Elevation	CEH min	CVH	R2	p value	AIC	Scheme	Model
Sim_1	0.23	0.21	0.67	0.58	0.57	0.00	5472.16	W	error
Sim_2	0.29	0.09	0.35	0.20	0.43	0.00	6282.21	W	error
Sim_3	-0.03	-0.18	-0.24	0.07	0.27	0.00	7052.05	W	error

Sim_4	0.38	0.13	0.39	0.46	0.55	0.00	5668.52	W	error
Sim_5	0.02	0.07	0.22	0.10	0.61	0.00	5255.83	W	error
Sim_6	0.32	0.18	0.38	0.51	0.49	0.00	6144.33	W	error
Sim_7	0.08	0.01	0.03	0.27	0.45	0.00	6206.36	W	error
Sim_8	-0.02	-0.03	0.17	0.42	0.36	0.00	6721.95	W	error
Sim_9	0.35	0.30	0.58	0.45	0.37	0.00	6685.87	W	error
Sim_10	-0.26	-0.32	-0.71	-0.13	0.49	0.00	6039.90	W	error
Sim_11	0.34	0.52	1.18	0.74	0.67	0.00	4837.75	W	error
Sim_12	0.16	-0.02	0.28	0.17	0.67	0.00	4707.50	W	error
Sim_13	-0.09	0.07	0.03	0.12	0.51	0.00	5893.32	W	error
Sim_14	0.06	0.25	0.45	0.55	0.53	0.00	5883.80	W	error
Sim_15	0.06	0.39	0.48	0.50	0.53	0.00	5862.50	W	error
Sim_16	0.36	0.19	0.20	0.26	0.50	0.00	6028.86	S	error
Sim_17	0.04	0.09	0.18	0.26	0.36	0.00	6719.30	W	error
Sim_18	0.24	-0.09	-0.03	-0.03	0.51	0.00	5969.91	W	error
Sim_19	0.08	0.22	0.51	0.64	0.47	0.00	6213.50	W	error
Sim_20	-0.01	0.32	0.65	0.74	0.64	0.00	5089.01	W	error
Sim_21	0.14	0.30	0.77	0.30	0.49	0.00	6042.70	W	error
Sim_22	-0.07	-0.05	-0.19	-0.03	0.49	0.00	6062.90	W	error
Sim_23	0.16	0.14	0.36	0.50	0.52	0.00	5957.58	W	error
Sim_24	0.25	0.05	0.23	-0.01	0.56	0.00	5663.08	W	error
Sim_25	-0.03	0.06	0.09	0.42	0.49	0.00	6047.70	W	error
Sim_26	-0.16	0.02	0.11	0.53	0.47	0.00	6220.85	W	error
Sim_27	0.19	0.19	0.57	0.16	0.55	0.00	5669.06	W	error
Sim_28	0.10	0.01	-0.14	0.11	0.52	0.00	5933.06	W	error
Sim_29	-0.07	-0.19	-0.46	0.08	0.73	0.00	4120.21	W	error
Sim_30	0.04	0.05	0.20	0.16	0.61	0.00	5142.16	W	error
Sim_31	0.18	-0.14	-0.29	0.09	0.73	0.00	4192.17	W	error
Sim_32	-0.03	0.23	0.47	0.39	0.54	0.00	5677.56	W	error
Sim_33	0.08	-0.08	-0.28	0.04	0.58	0.00	5562.73	W	error
Sim_34	0.09	-0.40	-0.87	-0.18	0.70	0.00	4593.75	W	error
Sim_35	0.17	0.21	0.12	0.24	0.53	0.00	5835.60	W	error
Sim_36	0.00	0.43	0.74	0.43	0.54	0.00	5784.49	W	error
Sim_37	0.24	0.02	-0.09	0.19	0.51	0.00	5964.50	W	error
Sim_38	0.19	0.12	0.06	-0.12	0.47	0.00	6247.21	W	error
Sim_39	0.30	0.26	0.40	0.43	0.51	0.00	5938.88	W	error
Sim_40	0.00	-0.30	-0.82	-0.29	0.50	0.00	6042.70	W	error
Sim_41	0.18	0.18	0.49	0.19	0.62	0.00	5001.18	W	error
Sim_42	0.10	-0.21	-0.65	-0.15	0.56	0.00	5627.19	W	error
Sim_43	0.11	0.43	0.88	0.65	0.55	0.00	5702.68	W	error
Sim_44	0.01	0.28	0.25	0.42	0.49	0.00	6104.46	W	error

Sim_45	0.17	0.24	0.53	0.28	0.57	0.00	5548.03	W	error
Sim_46	0.12	-0.09	0.05	0.25	0.42	0.00	6394.33	W	error
Sim_47	0.08	0.19	0.54	0.23	0.56	0.00	5682.39	W	error
Sim_48	0.13	0.10	0.30	0.05	0.70	0.00	4629.09	W	error
Sim_49	0.23	0.32	0.67	0.51	0.41	0.00	6538.12	W	error
Sim_50	-0.05	-0.16	-0.33	0.16	0.70	0.00	4574.20	W	error
Sim_51	0.04	-0.05	-0.08	0.16	0.49	0.00	5907.62	W	error
Sim_52	-0.12	-0.14	-0.44	0.18	0.65	0.00	5014.66	W	error
Sim_53	-0.22	-0.29	-0.64	0.00	0.60	0.00	5265.21	W	error
Sim_54	0.39	0.47	0.86	0.32	0.51	0.00	5993.49	W	error
Sim_55	0.15	0.14	0.46	0.03	0.56	0.00	5699.24	W	error
Sim_56	0.31	0.14	0.13	0.10	0.45	0.00	6312.43	W	error
Sim_57	0.10	0.37	0.67	0.53	0.52	0.00	5851.35	W	error
Sim_58	-0.07	0.08	0.20	0.26	0.49	0.00	6006.73	W	error
Sim_59	0.08	0.39	0.55	0.53	0.62	0.00	5214.90	W	error
Sim_60	-0.02	-0.20	-0.59	-0.01	0.57	0.00	5540.93	W	error
Sim_61	0.29	-0.19	-0.50	-0.24	0.48	0.00	6123.84	W	error
Sim_62	0.19	0.12	0.38	0.26	0.42	0.00	6458.96	W	error
Sim_63	-0.08	-0.11	-0.31	0.01	0.79	0.00	3327.07	W	error
Sim_64	0.22	0.16	0.14	-0.01	0.31	0.00	6916.03	W	error
Sim_65	0.21	0.42	1.01	0.75	0.59	0.00	5415.98	W	error
Sim_66	0.19	-0.04	-0.06	0.16	0.51	0.00	5994.86	W	error
Sim_67	-0.01	0.08	0.16	0.11	0.57	0.00	5621.55	W	error
Sim_68	-0.04	-0.39	-0.80	-0.23	0.61	0.00	5330.66	W	error
Sim_69	0.22	0.14	0.36	0.38	0.37	0.00	6587.96	W	error
Sim_70	0.37	0.23	0.64	0.38	0.54	0.00	5722.74	W	error
Sim_71	0.21	0.01	-0.16	0.11	0.63	0.00	5158.65	W	error
Sim_72	0.07	-0.03	-0.18	0.33	0.50	0.00	5990.12	W	error
Sim_73	0.10	-0.06	0.01	0.39	0.50	0.00	5996.61	W	error
Sim_74	-0.07	0.23	0.25	0.30	0.33	0.00	6844.54	W	error
Sim_75	0.10	0.15	0.46	0.09	0.68	0.00	4670.16	W	error
Sim_76	0.26	-0.14	-0.16	0.10	0.49	0.00	6059.24	S	error
Sim_77	0.25	-0.09	-0.18	0.00	0.53	0.00	5858.72	W	error
Sim_78	0.07	0.25	0.61	0.45	0.67	0.00	4755.73	W	error
Sim_79	-0.14	-0.01	0.06	0.26	0.61	0.00	5232.20	W	error
Sim_80	0.14	-0.10	-0.09	0.02	0.71	0.00	4405.10	W	error
Sim_81	-0.13	-0.02	-0.16	0.19	0.39	0.00	6513.01	S	error
Sim_82	0.16	-0.19	-0.53	-0.05	0.54	0.00	5770.56	W	error
Sim_83	0.17	0.02	0.00	0.22	0.29	0.00	7001.06	W	error
Sim_84	0.23	0.23	0.27	0.05	0.55	0.00	5725.01	W	error
Sim_85	0.19	0.04	0.03	0.06	0.32	0.00	6845.87	W	error

Sim_86	0.25	0.33	0.80	0.36	0.50	0.00	6085.63	W	error
Sim_87	0.17	0.02	0.00	0.24	0.41	0.00	6534.67	W	error
Sim_88	0.04	0.04	0.02	0.06	0.61	0.00	5280.01	W	error
Sim_89	0.08	-0.12	-0.33	0.08	0.61	0.00	5296.34	W	error
Sim_90	0.25	0.16	0.44	0.17	0.27	0.00	7046.48	W	error
Sim_91	0.04	-0.13	-0.15	0.05	0.51	0.00	5858.20	W	error
Sim_92	-0.08	0.14	0.18	0.29	0.55	0.00	5632.94	W	error
Sim_93	-0.13	0.11	0.26	0.45	0.38	0.00	6639.05	W	error
Sim_94	-0.08	0.11	0.27	0.46	0.51	0.00	5956.62	W	error
Sim_95	0.20	0.22	0.50	0.25	0.50	0.00	5984.61	W	error
Sim_96	0.07	0.26	0.50	0.10	0.55	0.00	5725.51	W	error
Sim_97	0.05	0.14	0.45	0.41	0.38	0.00	6633.92	W	error
Sim_98	0.11	-0.09	0.06	0.21	0.58	0.00	5581.72	W	error
Sim_99	-0.21	0.00	-0.33	0.15	0.56	0.00	5597.33	W	error
Sim_100	0.27	0.32	0.68	0.61	0.52	0.00	5913.50	W	error

Leache (SARs)

Simulation	CCV	Elevation	CEH min	CVH	R2	p-value	AIC	Scheme	Model
sim_1	0.16	0.18	0.50	0.43	0.59	0.00	5307.93	W	error
sim_2	0.33	0.11	0.40	0.28	0.37	0.00	6527.83	S	error
sim_3	-0.03	-0.19	-0.35	0.04	0.34	0.00	6792.11	W	error
sim_4	0.26	0.05	0.11	0.34	0.66	0.00	4847.04	W	error
sim_5	0.09	0.10	0.31	0.13	0.57	0.00	5477.13	W	error
sim_6	0.30	0.27	0.51	0.54	0.48	0.00	6147.73	W	error
sim_7	0.06	0.04	0.11	0.36	0.42	0.00	6367.86	W	error
sim_8	0.08	0.02	0.22	0.50	0.44	0.00	6319.07	W	error
sim_9	0.27	0.29	0.51	0.43	0.38	0.00	6635.79	W	error
sim_10	-0.28	-0.23	-0.57	-0.04	0.50	0.00	5975.05	W	error
sim_11	0.29	0.43	0.99	0.59	0.71	0.00	4488.60	W	error
sim_12	0.16	-0.03	0.25	0.07	0.69	0.00	4508.69	W	error
sim_13	-0.14	0.07	0.05	0.19	0.46	0.00	6156.88	W	error
sim_14	-0.09	0.12	0.25	0.45	0.59	0.00	5464.73	W	error
sim_15	-0.01	0.31	0.43	0.52	0.55	0.00	5705.90	W	error
sim_16	0.41	0.23	0.26	0.22	0.50	0.00	5997.91	S	error
sim_17	0.11	0.09	0.25	0.15	0.36	0.00	6637.95	W	error
sim_18	0.21	-0.13	-0.02	-0.04	0.50	0.00	5993.94	W	error
sim_19	0.12	0.29	0.72	0.81	0.44	0.00	6333.21	W	error
sim_20	-0.04	0.12	0.21	0.29	0.72	0.00	4344.57	W	error
sim_21	0.27	0.18	0.48	0.07	0.50	0.00	5918.99	W	error
sim_22	-0.08	0.04	0.03	0.31	0.44	0.00	6312.52	W	error
sim_23	0.11	0.17	0.40	0.60	0.47	0.00	6209.14	W	error

sim_24	0.46	0.13	0.37	0.07	0.59	0.00	5498.14	W	error
sim_25	-0.01	0.04	0.02	0.33	0.54	0.00	5739.85	W	error
sim_26	-0.17	0.00	0.18	0.60	0.48	0.00	6195.39	W	error
sim_27	0.24	0.19	0.53	0.09	0.55	0.00	5670.57	W	error
sim_28	0.14	-0.02	-0.17	0.13	0.53	0.00	5816.78	W	error
sim_29	-0.05	-0.10	-0.30	0.12	0.77	0.00	3660.89	W	error
sim_30	0.02	0.12	0.33	0.15	0.58	0.00	5268.15	W	error
sim_31	0.11	-0.20	-0.38	-0.03	0.75	0.00	3907.81	W	error
sim_32	0.11	0.40	0.74	0.58	0.49	0.00	6000.10	W	error
sim_33	0.14	-0.05	-0.20	0.02	0.51	0.00	5947.13	W	error
sim_34	0.11	-0.35	-0.78	-0.13	0.63	0.00	5162.14	W	error
sim_35	0.14	0.22	0.17	0.26	0.53	0.00	5850.01	W	error
sim_36	-0.02	0.37	0.66	0.43	0.57	0.00	5508.73	W	error
sim_37	0.06	-0.18	-0.49	-0.01	0.57	0.00	5590.15	W	error
sim_38	0.16	0.14	0.22	0.03	0.49	0.00	6148.31	W	error
sim_39	0.26	0.24	0.43	0.46	0.51	0.00	5912.43	W	error
sim_40	-0.03	-0.29	-0.89	-0.32	0.49	0.00	6122.98	W	error
sim_41	0.16	0.22	0.58	0.23	0.60	0.00	5100.51	W	error
sim_42	0.11	-0.22	-0.65	-0.17	0.52	0.00	5901.43	W	error
sim_43	0.13	0.43	0.83	0.58	0.50	0.00	6009.13	W	error
sim_44	-0.02	0.24	0.13	0.37	0.49	0.00	6128.87	W	error
sim_45	0.14	0.28	0.63	0.31	0.61	0.00	5251.83	W	error
sim_46	0.11	-0.13	-0.08	0.11	0.46	0.00	6216.38	W	error
sim_47	0.01	0.25	0.68	0.33	0.61	0.00	5160.71	W	error
sim_48	0.17	0.14	0.36	0.07	0.68	0.00	4796.13	W	error
sim_49	0.16	0.27	0.62	0.53	0.47	0.00	6215.92	W	error
sim_50	-0.02	-0.15	-0.31	0.16	0.69	0.00	4510.87	W	error
sim_51	0.01	-0.04	-0.05	0.21	0.49	0.00	5899.90	W	error
sim_52	-0.15	-0.16	-0.56	0.06	0.68	0.00	4786.31	W	error
sim_53	-0.20	-0.30	-0.64	-0.04	0.64	0.00	4938.95	W	error
sim_54	0.35	0.40	0.63	0.27	0.38	0.00	6606.80	W	error
sim_55	0.17	0.20	0.60	0.16	0.54	0.00	5849.89	W	error
sim_56	0.23	0.05	-0.08	0.05	0.56	0.00	5684.44	W	error
sim_57	0.06	0.31	0.57	0.43	0.61	0.00	5129.38	W	error
sim_58	-0.08	0.11	0.27	0.37	0.45	0.00	6157.84	W	error
sim_59	0.09	0.41	0.57	0.49	0.64	0.00	5085.13	W	error
sim_60	-0.07	-0.30	-0.79	-0.19	0.53	0.00	5783.56	W	error
sim_61	0.27	-0.11	-0.52	-0.24	0.49	0.00	6031.65	W	error
sim_62	0.22	0.13	0.45	0.32	0.41	0.00	6511.24	W	error
sim_63	-0.10	-0.04	-0.25	0.11	0.81	0.00	3224.24	W	error
sim_64	0.17	0.15	0.35	0.27	0.38	0.00	6630.00	S	error

sim_65	0.18	0.39	0.87	0.69	0.56	0.00	5576.11	W	error
sim_66	0.11	-0.20	-0.28	0.00	0.58	0.00	5559.80	W	error
sim_67	-0.04	0.07	0.14	0.13	0.59	0.00	5484.74	W	error
sim_68	-0.06	-0.35	-0.85	-0.20	0.61	0.00	5353.50	W	error
sim_69	0.20	0.10	0.31	0.28	0.37	0.00	6613.93	S	error
sim_70	0.32	0.24	0.66	0.47	0.47	0.00	6065.91	W	error
sim_71	0.22	-0.03	-0.17	0.07	0.63	0.00	5135.66	W	error
sim_72	0.02	-0.08	-0.24	0.35	0.55	0.00	5732.48	W	error
sim_73	0.10	-0.12	-0.27	0.00	0.60	0.00	5285.09	W	error
sim_74	0.00	0.24	0.32	0.35	0.39	0.00	6569.19	W	error
sim_75	0.22	0.19	0.57	0.15	0.67	0.00	4704.75	W	error
sim_76	0.21	-0.14	-0.17	0.01	0.50	0.00	6035.46	S	error
sim_77	0.19	-0.19	-0.40	-0.10	0.56	0.00	5690.67	W	error
sim_78	0.04	0.19	0.42	0.24	0.71	0.00	4383.38	W	error
sim_79	-0.05	0.08	0.18	0.19	0.67	0.00	4728.01	W	error
sim_80	0.13	-0.13	-0.12	0.03	0.68	0.00	4645.08	W	error
sim_81	-0.16	-0.02	-0.14	0.22	0.51	0.00	5930.83	W	error
sim_82	0.14	-0.23	-0.51	-0.05	0.53	0.00	5870.75	W	error
sim_83	0.11	-0.04	-0.05	0.24	0.33	0.00	6841.58	W	error
sim_84	0.25	0.32	0.47	0.25	0.55	0.00	5743.35	W	error
sim_85	0.26	0.07	0.04	0.11	0.37	0.00	6636.86	W	error
sim_86	0.24	0.31	0.79	0.39	0.45	0.00	6237.63	W	error
sim_87	0.18	-0.04	-0.17	0.07	0.46	0.00	6260.04	W	error
sim_88	0.09	0.01	-0.05	0.02	0.64	0.00	5017.78	W	error
sim_89	0.11	-0.09	-0.27	0.13	0.59	0.00	5466.46	W	error
sim_90	0.26	0.14	0.40	0.11	0.31	0.00	6924.41	W	error
sim_91	0.07	-0.11	-0.07	0.05	0.48	0.00	5931.81	W	error
sim_92	-0.10	0.16	0.17	0.34	0.54	0.00	5690.92	W	error
sim_93	-0.16	0.03	0.11	0.40	0.42	0.00	6432.89	W	error
sim_94	-0.09	0.11	0.38	0.46	0.42	0.00	6450.00	W	error
sim_95	0.25	0.28	0.63	0.38	0.46	0.00	6172.55	W	error
sim_96	0.06	0.28	0.51	0.13	0.59	0.00	5460.99	W	error
sim_97	0.08	0.05	0.23	0.15	0.36	0.00	6683.06	W	error
sim_98	0.04	-0.13	-0.12	0.04	0.59	0.00	5482.12	W	error
sim_99	-0.21	-0.07	-0.42	0.07	0.60	0.00	5305.72	W	error
sim_100	0.25	0.30	0.57	0.56	0.48	0.00	6139.18	S	error
