# Supplementary figures and images for: Exploring the causes underlying the latitudinal variation in range sizes: Evidence for Rapoport’s rule in spiny lizards (genus Sceloporus)
Source: PLoS One. 2024 Jul 9;19(7):e0306832. doi: 10.1371/journal.pone.0306832 (PMC11233011; doi:10.1371/journal.pone.0306832)

**S1 Fig.** Range size and Midpoints comparisons for all methods

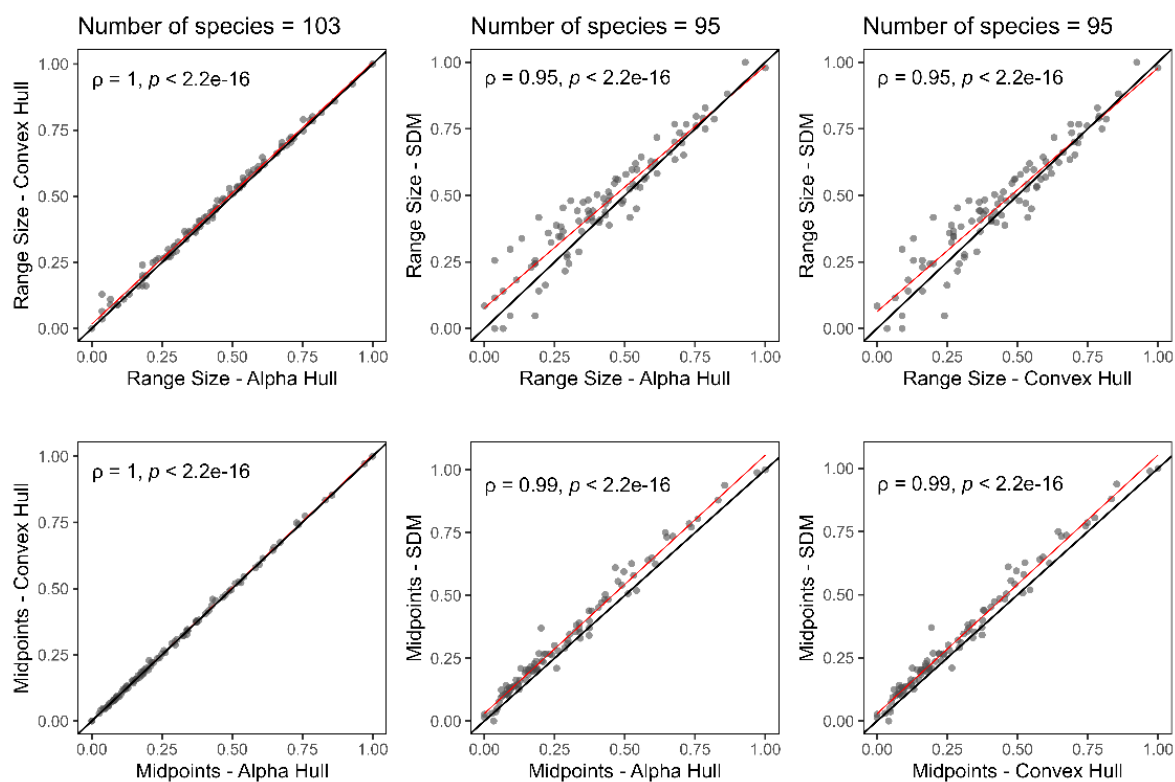

Supplement: S1 Fig — This figure includes the comparation for range size and midpoints estimations for the three methods employed in our study (Alpha-hull, Convex-hull and Species Distribution Models). (PDF) [file pone.0306832.s005.pdf]

**S2 Fig.** Frequency histograms for null OLS (cross-species and assemblage level)

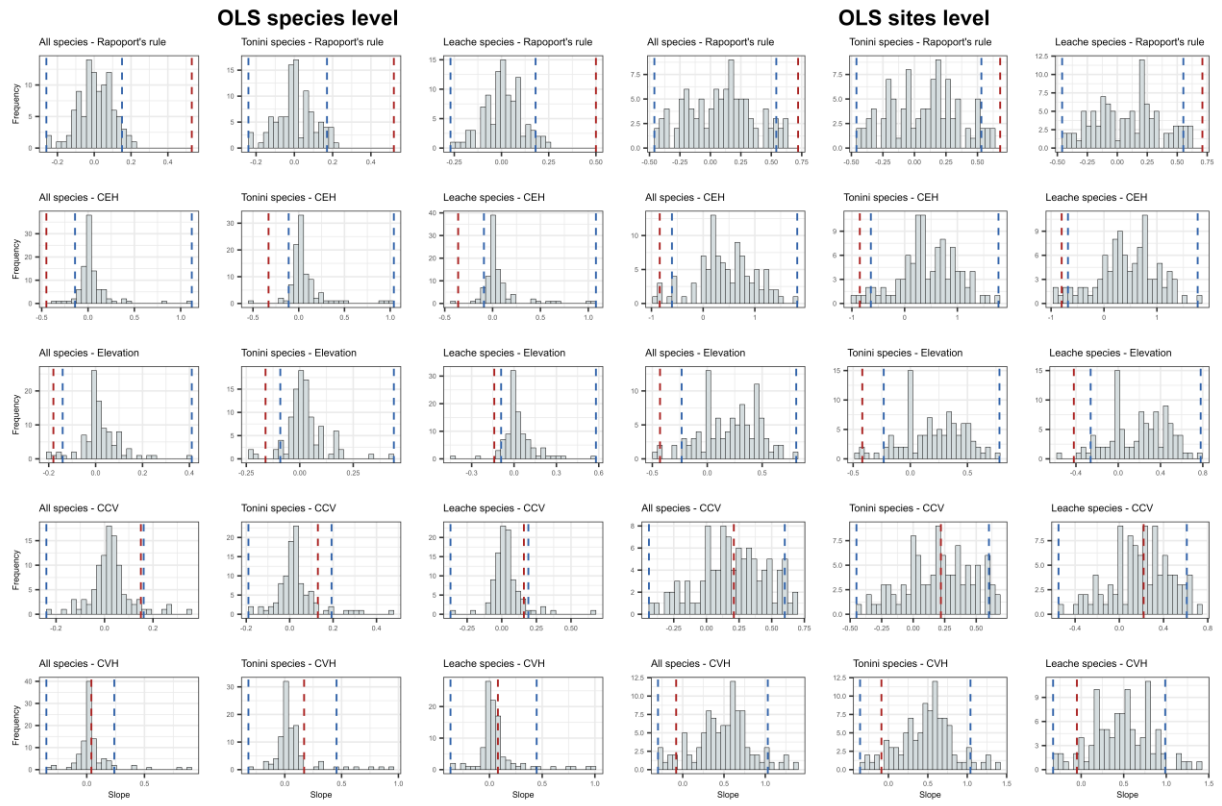

Supplement: S2 Fig — This figure shows the 100 simulated OLS coefficients at cross-species and assemblage level. (PDF) [file pone.0306832.s006.pdf]
