# Supplementary material for: Exploring the causes underlying the latitudinal variation in range sizes: Evidence for Rapoport’s rule in spiny lizards (genus Sceloporus)
Source: PLoS One. 2024 Jul 9;19(7):e0306832. doi: 10.1371/journal.pone.0306832 (PMC11233011; doi:10.1371/journal.pone.0306832)
Supplement: S1 Table — This table contains the number of occurrence records used to model the distribution ranges. (PDF) [file pone.0306832.s007.pdf]

Species	Number of records
Sceloporus acanthinus	62
Sceloporus adleri	15
Sceloporus aeneus	154
Sceloporus albiventris	62
Sceloporus anahuacus	34
Sceloporus arenicolus	42
Sceloporus asper	33
Sceloporus aurantius	4
Sceloporus aureolus	48
Sceloporus bicanthalis	80
Sceloporus bimaculosus	150
Sceloporus brownorum	16
Sceloporus bulleri	47
Sceloporus carinatus	41
Sceloporus cautus	87
Sceloporus chaneyi	8
Sceloporus chrysostictus	204
Sceloporus clarkii	703
Sceloporus consobrinus	1595
Sceloporus couchii	42
Sceloporus cowlesi	630
Sceloporus cozumelae	29
Sceloporus cryptus	7
Sceloporus cupreus	8
Sceloporus cyanogenys	231
Sceloporus cyanostictus	18
Sceloporus dixoni	10
Sceloporus druckercolini	8
Sceloporus dugesii	148
Sceloporus edbelli	89
Sceloporus edwardtaylori	23
Sceloporus esperanzae	3
Sceloporus exsul	4
Sceloporus formosus	164
Sceloporus gadoviae	88
Sceloporus gadsdeni	4
Sceloporus goldmani	22
Sceloporus graciosus	1534
Sceloporus grammicus	863
Sceloporus grandaevus	3
Sceloporus heterolepis	39

Sceloporus hondurensis	27
Sceloporus horridus	379
Sceloporus huichol	4
Sceloporus hunsakeri	28
Sceloporus insignis	12
Sceloporus internasalis	46
Sceloporus jalapae	74
Sceloporus jarrovii	568
Sceloporus lemosespinali	41
Sceloporus licki	33
Sceloporus lundelli	77
Sceloporus macdougalli	3
Sceloporus maculosus	27
Sceloporus magister	1162
Sceloporus malachiticus	122
Sceloporus megalepidurus	80
Sceloporus melanorhinus	235
Sceloporus merriami	174
Sceloporus minor	195
Sceloporus mucronatus	213
Sceloporus nelsoni	169
Sceloporus oberon	31
Sceloporus occidentalis	1992
Sceloporus ochoterenae	42
Sceloporus olivaceus	950
Sceloporus omiltemanus	64
Sceloporus orcutti	164
Sceloporus ornatus	56
Sceloporus palaciosi	20
Sceloporus parvus	160
Sceloporus poinsettii	803
Sceloporus pyrocephalus	112
Sceloporus salvini	36
Sceloporus samcolemani	14
Sceloporus scalaris	271
Sceloporus schmidti	3
Sceloporus scitulus	14
Sceloporus serrifer	166
Sceloporus shannonorum	9
Sceloporus siniferus	263
Sceloporus slevini	118
Sceloporus smaragdinus	59
Sceloporus smithi	33

Sceloporus spinosus	587
Sceloporus squamosus	85
Sceloporus stejnegeri	7
Sceloporus subniger	60
Sceloporus subpictus	10
Sceloporus sugillatus	8
Sceloporus taeniocnemis	73
Sceloporus tanneri	15
Sceloporus teapensis	141
Sceloporus torquatus	616
Sceloporus tristichus	844
Sceloporus undulatus	3836
Sceloporus unicanthalis	10
Sceloporus uniformis	335
Sceloporus utiformis	181
Sceloporus variabilis	817
Sceloporus virgatus	105
Sceloporus woodi	59
Sceloporus zosteromus	146
**Total**	**24336**
