# Supplementary material for: Exploring the causes underlying the latitudinal variation in range sizes: Evidence for Rapoport’s rule in spiny lizards (genus Sceloporus)
Source: PLoS One. 2024 Jul 9;19(7):e0306832. doi: 10.1371/journal.pone.0306832 (PMC11233011; doi:10.1371/journal.pone.0306832)
Supplement: S2 Table — This table contains the range size estimation for all the species through alpha-hulls. (PDF) [file pone.0306832.s008.pdf]

S1 Table. Range size estimated with alpha hulls for all the species

Species	Range size
Sceloporus acanthinus	163253.25
Sceloporus adleri	33882.75
Sceloporus aeneus	304944.75
Sceloporus albiventris	101648.25
Sceloporus anahuacus	61605
Sceloporus arenicolus	67765.5
Sceloporus asper	92407.5
Sceloporus aurantius	18481.5
Sceloporus aureolus	77006.25
Sceloporus bicanthalis	129370.5
Sceloporus bimaculosus	760821.75
Sceloporus brownorum	64685.25
Sceloporus bulleri	221778
Sceloporus carinatus	67765.5
Sceloporus cautus	212537.25
Sceloporus chaneyi	15401.25
Sceloporus chrysostictus	169413.75
Sceloporus clarkii	933315.75
Sceloporus consobrinus	3523806
Sceloporus couchii	117049.5
Sceloporus cowlesi	896352.75
Sceloporus cozumelae	70845.75
Sceloporus cryptus	27722.25
Sceloporus cupreus	18481.5
Sceloporus cyanogenys	360389.25
Sceloporus cyanostictus	36963
Sceloporus dixoni	21561.75
Sceloporus druckercolini	15401.25
Sceloporus dugesii	249500.25
Sceloporus edbelli	369630
Sceloporus edwardtaylori	36963
Sceloporus esperanzae	6160.5
Sceloporus exsul	12321
Sceloporus formosus	301864.5
Sceloporus gadoviae	187895.25
Sceloporus gadsdeni	12321
Sceloporus goldmani	113969.25
Sceloporus graciosus	2384113.5
Sceloporus grammicus	1210538.25
Sceloporus grandaevus	206376.75
Sceloporus heterolepis	181734.75
Sceloporus hondurensis	73926

Sceloporus horridus	455877
Sceloporus huichol	9240.75
Sceloporus hunsakeri	12321
Sceloporus insignis	40043.25
Sceloporus internasalis	141691.5
Sceloporus jalapae	126290.25
Sceloporus jarrovii	1210538.25
Sceloporus lemosespinali	169413.75
Sceloporus licki	52364.25
Sceloporus lundelli	150932.25
Sceloporus macdougalli	6160.5
Sceloporus maculosus	58524.75
Sceloporus magister	1795785.75
Sceloporus malachiticus	409673.25
Sceloporus megalepidurus	117049.5
Sceloporus melanorhinus	455877
Sceloporus merriami	335747.25
Sceloporus minor	394272
Sceloporus mucronatus	286463.25
Sceloporus nelsoni	332667
Sceloporus oberon	181734.75
Sceloporus occidentalis	1478520
Sceloporus ochoterenae	64685.25
Sceloporus olivaceus	847068.75
Sceloporus omiltemanus	184815
Sceloporus orcutti	243339.75
Sceloporus ornatus	104728.5
Sceloporus palaciosi	36963
Sceloporus parvus	326506.5
Sceloporus poinsettii	1404594
Sceloporus pyrocephalus	169413.75
Sceloporus salvini	147852
Sceloporus samcolemani	18481.5
Sceloporus scalaris	766982.25
Sceloporus schmidti	9240.75
Sceloporus scitulus	36963
Sceloporus serrifer	760821.75
Sceloporus shannonorum	80086.5
Sceloporus siniferus	520562.25
Sceloporus slevini	499000.5
Sceloporus smaragdinus	95487.75
Sceloporus smithi	49284
Sceloporus spinosus	924075
Sceloporus squamosus	289543.5

Sceloporus stejnegeri	15401.25
Sceloporus subniger	95487.75
Sceloporus subpictus	21561.75
Sceloporus sugillatus	40043.25
Sceloporus taeniocnemis	120129.75
Sceloporus tanneri	24642
Sceloporus teapensis	326506.5
Sceloporus torquatus	680735.25
Sceloporus tristichus	979519.5
Sceloporus undulatus	5436641.25
Sceloporus unicanthalis	46203.75
Sceloporus uniformis	517482
Sceloporus utiformis	206376.75
Sceloporus variabilis	1478520
Sceloporus virgatus	264901.5
Sceloporus woodi	73926
Sceloporus zosteromus	129370.5
