# Supplementary material for: Exploring the causes underlying the latitudinal variation in range sizes: Evidence for Rapoport’s rule in spiny lizards (genus Sceloporus)
Source: PLoS One. 2024 Jul 9;19(7):e0306832. doi: 10.1371/journal.pone.0306832 (PMC11233011; doi:10.1371/journal.pone.0306832)
Supplement: Attachment — Submitted filename: Review Comments.pdf [file pone.0306832.s009.pdf]

Comments to Authors:

Dear Authors,

It is really an interesting work exploring species range sizes along latitude. Also, ectothermic and least studied groups like reptiles (especially lizards) are the best models for such experiments.

With my limited knowledge on this analytical approaches, I feel like the analysis is more like a test of Climatic Variability Hypothesis rather than Rapoport's rule. Because, the analyses conducted in this paper first explore the geographical range of each species using SDM or extent of occurrences (polygon methods), then modeling these range sizes with variability in climatic variables (current and historic) and elevation. In method, use of 'range size in km2 (response variable) and latitudinal midpoint (explanatory variable) ' shows that the overall geographical range size is taken as the response variable instead of the latitudinal range sizes (or range limits).

The (latitudinal) Rapoport's rule is a test of latitudinal range sizes of species (not the overall geographical distribution range size) with their mid-point distribution across latitude. Meanwhile Climatic Variability Hypothesis explore multiple dimensions of species ranges with respect to climatic variability in any geographical space (latitudinal/ altitudinal) as it is done in this paper. If this is correct, I would request authors to set the objectives of the paper in that aspect.

The following paper (Reference 90 already cited in the MS) is a good example : Pintor, A. F., Schwarzkopf, L., & Krockenberger, A. K. (2015). Rapoport's Rule: Do climatic variability gradients shape range extent?. Ecological Monographs, 85(4), 643-659.

Apart from the above, I would also like to see the sample sizes which is the number of occurrence points used for modeling each species in the analysis. In methods the authors mentioned that species with at least three validated points were also used. I am not sure if such a small number of points can define the climatic variables or range sizes (or if it is sufficient for SDMs) of a species.

Overall, the paper is a nice experiment with strong analytical back up. However, it would be really great if you could clarify the basic hypothesis tested here as the title emphasis on testing Rapoport's rule.
