# Supplementary material for: Exploring the causes underlying the latitudinal variation in range sizes: Evidence for Rapoport’s rule in spiny lizards (genus Sceloporus)
Source: PLoS One. 2024 Jul 9;19(7):e0306832. doi: 10.1371/journal.pone.0306832 (PMC11233011; doi:10.1371/journal.pone.0306832)
Supplement: Attachment — Submitted filename: PONE-D-23-29199_reviewer_BS.pdf [file pone.0306832.s010.pdf]

PLOS ONE

Exploring the causes underlying the latitudinal variation in range sizes: evidence for Rapoport's rule in spiny lizards (genus Sceloporus)

--Manuscript Draft--

**Manuscript Number:**	PONE-D-23-29199
**Article Type:**	Research Article
**Full Title:**	Exploring the causes underlying the latitudinal variation in range sizes: evidence for Rapoport's rule in spiny lizards (genus Sceloporus)
**Short Title:**	Rapoport's rule in spiny lizards
**Corresponding Author:**	Kevin López Reyes, MSc<br>UNAM: Universidad Nacional Autonoma de Mexico<br>Mérida, MEXICO
**Keywords:**	Rapoport's rule; Range size; Lizards; Sceloporus; Temperature; Elevation
**Abstract:**	Species' range size is a fundamental unit of analysis in biodiversity research, given its association with extinction risk and species richness. One of its most notable patterns is its positive relationship with latitude, which is considered an ecogeographical rule called Rapoport's rule. Despite this rule being confirmed for various taxonomic groups, its validity has been widely discussed and several taxa still lack a formal assessment. Different hypotheses have been proposed to explain their potential mechanisms, with those related to temperature and elevational variation the most supported thus far. In this study, we employed two level of analyses (cross-species and assemblage) to investigate the validity of Rapoport's rule in spiny lizards (genus Sceloporus). We evaluated four environmental-related hypotheses (minimum temperature, temperature range, temperature stability since the last glacial maximum, and elevation) posed to explain such pattern and compared our observations to those expected under a null model of range position. Our results provided support for Rapoport's rule at both levels of analyses, contrasting with null expectations. Consistently, minimum temperature and elevation were the most relevant variables explaining the spatial variation in range size. At the cross-species level, our null simulations revealed that both variables deviated significantly from random expectations. Conversely, at the assemblage level, none of the variables were statistically different from the expected relationships. We discussed the implication of our findings in relation to the ecology and evolution of spiny lizards.
**Order of Authors:**	Kevin López-Reyes, MSc
	Carlos Yáñez-Arenas
	Fabricio Villalobos
**Additional Information:**	
**Question**	**Response**
**Financial Disclosure**<br><br>Enter a financial disclosure statement that describes the sources of funding for the work included in this submission. Review the submission guidelines for detailed requirements. View published research articles from *PLOS ONE* for specific examples.<br><br>This statement is required for submission and **will appear in the published article** if the submission is accepted. Please make	KLR received support from the Consejo Nacional de Humanidades Ciencias y Tecnologías (CONAHCYT; CVU: 1010702). The funders had no role in study design, data collection and analysis, decision to publish, or preparation of the manuscript.

sure it is accurate.<br><br>**Unfunded studies**<br>Enter: *The author(s) received no specific funding for this work.*<br><br>**Funded studies**<br>Enter a statement with the following details:<br>• Initials of the authors who received each award<br>• Grant numbers awarded to each author<br>• The full name of each funder<br>• URL of each funder website<br>• Did the sponsors or funders play any role in the study design, data collection and analysis, decision to publish, or preparation of the manuscript?<br>• **NO** - Include this sentence at the end of your statement: *The funders had no role in study design, data collection and analysis, decision to publish, or preparation of the manuscript.*<br>• **YES** - Specify the role(s) played.<br><br>* typeset	
**Competing Interests**<br><br>Use the instructions below to enter a competing interest statement for this submission. On behalf of all authors, disclose any competing interests that could be perceived to bias this work—acknowledging all financial support and any other relevant financial or non-financial competing interests.<br><br>This statement is required for submission and **will appear in the published article** if the submission is accepted. Please make sure it is accurate and that any funding sources listed in your Funding Information later in the submission form are also declared in your Financial Disclosure statement.<br><br>View published research articles from *PLOS ONE* for specific examples.	The authors have declared that no competing interests exist.

**NO authors have competing interests**<br><br>Enter: *The authors have declared that no competing interests exist.*<br><br>**Authors with competing interests**<br><br>Enter competing interest details beginning with this statement:<br><br>*I have read the journal's policy and the authors of this manuscript have the following competing interests: [insert competing interests here]*<br><br>* typeset	
**Ethics Statement**<br><br>Enter an ethics statement for this submission. This statement is required if the study involved:<br><br>• Human participants<br>• Human specimens or tissue<br>• Vertebrate animals or cephalopods<br>• Vertebrate embryos or tissues<br>• Field research<br><br>Write "N/A" if the submission does not require an ethics statement.<br><br>General guidance is provided below. Consult the submission guidelines for detailed instructions. **Make sure that all information entered here is included in the Methods section of the manuscript.**	N/A

Powered by Editorial Manager® and ProduXion Manager® from Aries Systems Corporation

**Format for specific study types**<br><br>**Human Subject Research (involving human participants and/or tissue)**<br>• Give the name of the institutional review board or ethics committee that approved the study<br>• Include the approval number and/or a statement indicating approval of this research<br>• Indicate the form of consent obtained (written/oral) or the reason that consent was not obtained (e.g. the data were analyzed anonymously)<br><br>**Animal Research (involving vertebrate animals, embryos or tissues)**<br>• Provide the name of the Institutional Animal Care and Use Committee (IACUC) or other relevant ethics board that reviewed the study protocol, and indicate whether they approved this research or granted a formal waiver of ethical approval<br>• Include an approval number if one was obtained<br>• If the study involved *non-human primates*, add *additional details* about animal welfare and steps taken to ameliorate suffering<br>• If anesthesia, euthanasia, or any kind of animal sacrifice is part of the study, include briefly which substances and/or methods were applied<br><br>**Field Research**<br><br>Include the following details if this study involves the collection of plant, animal, or other materials from a natural setting:<br>• Field permit number<br>• Name of the institution or relevant body that granted permission	
**Data Availability**<br><br>Authors are required to make all data underlying the findings described fully available, without restriction, and from the time of publication. PLOS allows rare exceptions to address legal and ethical concerns. See the PLOS Data Policy and FAQ for detailed information.	Yes - all data are fully available without restriction

A Data Availability Statement describing where the data can be found is required at submission. Your answers to this question constitute the Data Availability Statement and **will be published in the article**, if accepted.<br><br>**Important:** Stating 'data available on request from the author' is not sufficient. If your data are only available upon request, select 'No' for the first question and explain your exceptional situation in the text box.<br><br>Do the authors confirm that all data underlying the findings described in their manuscript are fully available without restriction?	
**Describe where the data may be found in full sentences. If you are copying our sample text, replace any instances of XXX with the appropriate details.**<br><br>• If the data are **held or will be held in a public repository**, include URLs, accession numbers or DOIs. If this information will only be available after acceptance, indicate this by ticking the box below. For example: *All XXX files are available from the XXX database (accession number(s) XXX, XXX.).*<br>• If the data are all contained **within the manuscript and/or Supporting Information files**, enter the following: *All relevant data are within the manuscript and its Supporting Information files.*<br>• If neither of these applies but you are able to provide **details of access elsewhere**, with or without limitations, please do so. For example:<br><br>*Data cannot be shared publicly because of [XXX]. Data are available from the XXX Institutional Data Access / Ethics Committee (contact via XXX) for researchers who meet the criteria for access to confidential data.*<br><br>*The data underlying the results presented in the study are available from (include the name of the third party*	The data underlying the results presented in the study are available from GBIF (www.gbif.org; doi: 10.15468/dl.5234bm) and WorldClim (https://www.worldclim.org/data/worldclim21.html).

Powered by Editorial Manager® and ProduXion Manager® from Aries Systems Corporation

and contact information or URL).<br>• This text is appropriate if the data are owned by a third party and authors do not have permission to share the data.<br><br>* typeset	
Additional data availability information:	

Powered by Editorial Manager® and ProduXion Manager® from Aries Systems Corporation

Manuscript Click here to access/download;Manuscript;LopezReyes_etal_Draft.docx

Exploring the causes underlying the latitudinal variation in range sizes: evidence for Rapoport's rule in spiny lizards (genus *Sceloporus*)

Kevin López-Reyes [1,2,3*], Carlos Yáñez-Arenas [2*], Fabricio Villalobos [3*]

[1]. Posgrado en Ciencias Biológicas, Universidad Nacional Autónoma de México (UNAM)

[2]. Laboratorio de Ecología Geográfica, Unidad Académica Sisal, Facultad de Ciencias, Unidad de Conservación de la Biodiversidad, Parque Científico y Tecnológico de Yucatán, Universidad Nacional Autónoma de México, Mérida, Yucatán, México

[3]. Laboratorio de Macroecología Evolutiva, Red de Biología Evolutiva, Instituto de Ecología A.C. – INECOL, Xalapa, Veracruz, México

*Corresponding authors: Kevin López-Reyes, Carlos Yáñez-Arenas and Fabricio Villalobos

E-mail: lopezreyes.ka@gmail.com, lichoso@gmail.com, fabricio.villalobos@gmail.com

Abstract

Species' range size is a fundamental unit of analysis in biodiversity research, given its association with extinction risk and species richness. One of its most notable patterns is its positive relationship with latitude, which is considered an ecogeographical rule called Rapoport's rule. Despite this rule being confirmed for various taxonomic groups, its validity has been widely discussed and several taxa still lack a formal assessment. Different hypotheses have been proposed to explain their potential mechanisms, with those related to temperature and elevational variation the most supported thus far. In this study, we employed two level of analyses (cross-species and assemblage) to investigate the validity of Rapoport's rule in spiny lizards (genus *Sceloporus*). We evaluated four environmental-related hypotheses (minimum temperature, temperature range, temperature stability since the last glacial maximum, and elevation) posed to explain such pattern and compared our observations to those expected under a null model of range position. Our results provided support for Rapoport's rule at both levels of analyses, contrasting with null expectations. Consistently, minimum temperature and elevation were the most relevant variables explaining the spatial variation in range size. At the cross-species level, our null simulations revealed that both variables deviated significantly from random expectations. Conversely, at the assemblage level, none of the variables were statistically different from the expected relationships. We discussed the implication of our findings in relation to the ecology and evolution of spiny lizards.

Keywords: Rapoport's rule, Range size, Lizards, *Sceloporus,* Temperature, Elevation

1. Introduction

In ecology and biogeography, the concept of “species distributional area” refers to the geographical regions of the planet where individuals of a specific species have been observed to occur. These areas encompass the ecological conditions that allow the species to have non-ephemeral interactions with the environment [1,2]. Previous studies have confirmed that the size of species’ distributional areas, also known as range size, is inversely related to species richness [3] and with the risk of extinction [4]. Consequently, understanding the spatial variation of range size can provide valuable insights into global patterns of biodiversity and inform conservation actions. One of the best-known macroecological patterns is the positive relationship between range size and latitude, also known as Rapoport’s rule [5]. Although Rapoport’s rule has been supported for various taxonomic groups [6–8], it’s validity has received strong criticism. For instance, the idea that this rule might be only an effect observed primarily in regions of the Northern Hemisphere, especially in the Americas [9–11]. Furthermore, despite the great interest in this rule, several species-rich groups still lack a proper assessment of their range size latitudinal patterns and their potential causes. Reptiles are an example of such groups, as they have received less attention compared to other extensively studied groups of terrestrial vertebrates, such as birds [8,12,13], mammals [6,14], and amphibians [15]. Among reptiles, lizards have received less attention in the study of macroecological patterns and their potential mechanisms [16,17], which poses a challenge to the generalization of Rapoport’s rule.

A crucial step towards addressing this knowledge gap consists in evaluating Rapoport’s rule in such groups, as well as understanding the mechanisms driving the spatial

variation in their range sizes. In this context, previous studies have suggested that the latitudinal variation in range size is primarily driven by spatial variation in temperature (current and past) and elevation, mainly through their influence on species' physiological tolerance and dispersal abilities [15,18–20]. Particularly for lizards, these attributes play a crucial role in their dispersal ability because they are closely associated with their thermal tolerance breadth and mobility [21–23]. The "climate variability hypothesis" [5] suggests that species present at northern latitudes tend to be thermal generalists due to the high temperature variability experienced in such areas, which in turn allows them to have larger range sizes compared to their tropical counterparts. Alternatively, Pither [24] suggested the "climate extremes hypothesis" in which, thermal limits are what restrict range sizes. According to this hypothesis, species able to tolerate more extreme temperature limits, namely cold ones, will exhibit larger range sizes. Another explanation for Rapoport's rule centers on historical climate stability [25]. Regions with stable climates over extended periods of time facilitate the persistence of species with small ranges. In contrast, areas characterized by greater climate variation over time, such as those observed in northern latitudes, primarily support species with larger range sizes. This is because species with smaller range sizes are more susceptible to extinction processes such as those exerted by climatic instability [25–27]. One additional factor that can influence the latitudinal gradient in range size is elevation [15,28]. In the tropics, there is greater temperature variation between different elevation zones compared to temperate regions. Therefore, tropical mountains act as more restrictive physiological barriers [28]. Consequently, tropical species will face greater challenges in expanding their range size across elevational gradients compared to temperate species.

In addition to these ecological hypotheses, other models have emerged in an attempt to explain the spatial variation in range sizes. For instance, the Mid-Domain Effect (MDE) provides a “non-biological” explanation for species distribution patterns, attributing them to the geometric constraints imposed by the shape of the geographic domain [29]. According to this model, species’ ranges are randomly arranged within a domain, where regions with greater availability of area (usually the center of the domain) tend to have greater overlap in species ranges (hence higher species richness) as well as larger range sizes [9,29]. This scenario can be recreated using null models that randomize the geographic position of the species’ ranges while maintaining their size [30]. This allows breaking the relationship between the environment and the position and size of the geographic range, making them useful for comparing the environmental variation experienced by species in their original positions with null observations where theoretically the environment has no effect on range size [6,31,32]. Consequently, it is crucial to include null models when assessing latitudinal gradients in range sizes and their relationship with potential determinants.

Compared to other vertebrate groups, especially in North America, there is a noticeable knowledge gap regarding the range size pattern in lizards. To our current understanding, no formal analysis has been carried out regarding the latitudinal variation of range sizes in lizards and their underlying mechanisms within this particular region. Spiny lizards (genus *Sceloporus*) represent an ideal group to fill this gap. This genus encompass ~116 species and is found in a wide variety of thermal and topographic regimes [33]. Moreover, although some of its species are present in Central America, its distribution is predominantly in North America [34]. Besides, although phylogenetic [35,36], ecological [37–39] and evolutionary [34,40–44] aspects have been previously evaluated for this group, its main macroecological patterns and determinants remain unevaluated. In this study, we

evaluated the validity of Rapoport's rule in genus *Sceloporus* and explored the importance of four ecological hypotheses (climate variability, climate extremes, historical climate stability, and elevation; see Table 1) to explain their latitudinal range size variation at two levels of analysis (cross-species and assemblage). To do that, we performed spatial and phylogenetic multiple regressions and our results were contrasted with null models to determine if the patterns found in this study can be recreated assuming no effect of environmental gradients. This study represents the first formal assessment of geographic variation in range size for this genus, as well as its potential determinants and provides valuable insights into its geographic and ecological knowledge.

Table 1. Ecological hypotheses evaluated to explain the latitudinal variation in range size for *Sceloporus* lizards within this study.

Hypothesis	Prediction	Explanation	Variable	Source
Historical climate stability [25,26]	+	Greater climate variation over time, especially in northern latitudes, predominantly supports species with larger range sizes. As species with smaller ranges are more susceptible to extinction.	Climate change velocity	Sandel et al., 2011
Climate variability [5]	+	Species found in northern latitudes tend to be thermal generalists, which allows them to have larger range sizes.	Temperature annual range	WorldClim
Climate extremes [24]	-	Species that are able to tolerate more extreme temperature limits, namely colder ones, will exhibit broader distribution areas.	Minimum temperature of the coldest month	WorldClim

Elevation [28]	-	Tropical mountains pose greater physiological barriers for species to expand their range sizes.	Digital elevation model	SRTM

2. Materials and Methods

All data cleaning procedures, model construction, and statistical analyses mentioned in the subsequent sections were conducted using the R software version 4.2.2 [45].

2.1 Geographic data

2.1.1 Occurrence records

Presence data, namely occurrence records, of *Sceloporus* species were obtained through Global Biodiversity Information Facility [46] and Naturalista [47]. The information from both repositories was integrated into a single species-level dataset, where taxonomic harmonization was performed using Uetz *et al.* [33] as the taxonomic authority. Then, records without geographic coordinates and duplicates were removed using the function “clean_dup” from the “ntbox” package [48]. Due to the inherent biases associated with sampling efforts and geographical inaccuracies, we performed an additional cleaning process. First, to reduce spatial autocorrelation, we utilized the “gridSample” function from the “dismo” package [49]. This function allowed us to retain a single presence record per cell, based on a 10 arc-minute raster resolution, which corresponds to approximately 340 square kilometers. Then, to eliminate geographically atypical data, we employed the “cc_outl” function from the “CoordinateCleaner” package [50]. In this process, we initially calculated the distances to the nearest neighbors for each presence record. If the distance exceeded two times the interquartile range of the average distance of all observations, the

record was classified as a geographically atypical observation and removed from the analysis.

2.1.2 Geographic ranges

We ensured independence of range sizes and midpoint estimations by using three distinct approaches for species geographic ranges estimation. We utilized one method based on a correlative approach between occurrences and climate (species distribution modeling), as well as two polygon-based estimations: **1) Convex hulls:** defined as the minimum convex set that contains a finite series of points, connecting the outermost points, where internal angles are less than 180 degrees [51] and **2) Alpha-hulls:** which allow the construction of non-convex sets by adjusting the shape of convex polygons based on an alpha parameter, resulting in shapes that may include holes or empty spaces within [52].

Given that the polygon construction is based on Voronoi triangulation, for the alpha and convex hulls we only considered species with a minimum of three distinct presence records after data depuration. To construct the convex-hulls, we used the “rangemap_hull” function from the “rangemap” package [53]. Alpha-hulls were estimated using the “getDynamicAlphaHull” function from the “rangeBuilder” package [54], which starts with an initial alpha value and gradually increases it until two conditions are met: 1) The desired percentage of points to be included within the polygon (in our case, 100%) and 2) The maximum number of disjoint distributions allowed (since this study did not consider disjoint distributions, only one alpha polygon was generated per species). Both methods used a buffer of 50 km, which represents the observed maximum distance of population separation within this genus [55].

To construct the species distribution models we employed the maximum entropy algorithm Maxent [56]. This analysis considered only those species with a minimum of five occurrence records after data cleaning. The presence records were then divided into two subsets: training and validation. For species with less than 25 occurrence records, a random partitioning was performed using 60% of the records for training and 40% for validation. The remaining species' records were partitioned using the "get.checkerboard1" function from the "ENMeval" package [57]. This function employs a checkerboard pattern to divide the observations, using the cells of an input raster as the basis. In our case, the raster had a resolution of ~340 km^2. After this, we collected the 19 bioclimatic variables from WorldClim version 2.1 (www.worldclim.org), which represent annual temperature and precipitation patterns between the years 1970 and 2000 [58]. Additionally, a digital elevation model derived from the Shuttle Radar Topographic Mission (SRTM; https://earthexplorer.usgs.gov) was included in the analysis [59]. The variables from both repositories were obtained at a ~340 km^2 resolution. Initially, bioclimatic variables 8, 9, 18, and 19 were removed due to previously demonstrated artifacts in their construction [60]. Afterwards, a variance inflation factor analysis was conducted to reduce multicollinearity among variables using the "vifstep" function from the "usdm" package [61]. Based on this procedure, the variables used for modeling were the digital elevation model and the following bioclimatic variables: 2 (mean diurnal range), 7 (temperature annual range), 10 (mean temperature of warmest quarter), 13 (precipitation of wettest month), 14 (precipitation of driest month), and 15 (precipitation seasonality). These variables were cropped to a calibration region for each species, defined based on the convex-hulls. Then, for each species, optimal parameters for modeling were obtained through a series of candidate models, which were constructed and evaluated using the "kuenm_cal_swd"

function from the “kuenm” package [62]. Based on this function, we generated the following combinations for different types of response curves: “linear”, “linear and quadratic”, “linear, quadratic and product”, “linear, quadratic, product and threshold” and “linear, quadratic, product, threshold and hinge”. We also tried different regularization multipliers (1, 2, 3, 4). After, the selection of the best models was done using three hierarchical criteria: 1) Statistical significance: where models performed better than expected by chance, i.e., the average area under the curve greater than 1, using partial receiver operating characteristic analysis [63], 2) Predictive ability: we selected models that correctly anticipated at least 90% of the presence records, resulting in a 10% omission rate [56] and 3) Complexity: using the small-sample corrected Akaike Information Criterion, we selected models with a difference of fewer than two units [64]. Finally, using the “kuenm_mod_swd” function, the final model was transferred to a region representing a hypothesis of historical accessibility for each species, referred to as “M” sensu Soberon and Peterson [65], constructed with terrestrial ecoregions [66]. To perform this, we included into the “M” those ecoregions that had at least one presence record. Subsequently, the continuous models generated through Maxent modeling were transformed into binary representations of distribution areas using the “rangemap_enm” function from the “rangemap” package [53]. The binarization threshold to obtain the final range map of each species was determined as the minimum value that encompassed the 90% of environmentally nearest records.

2.2 Environmental predictors of range size

To evaluate the environmental hypotheses posed to explain Rapoport’s rule, we used a set of predictor variables. The climate variability hypothesis was assessed using the

temperature annual range, whereas the climate extremes hypothesis was evaluated using the minimum temperature of the coldest month. Both variables were obtained from WorldClim version 2.1 (https://www.worldclim.org/). The hypothesis of elevation was evaluated using the previously mentioned digital elevation model. To represent historical climate stability, we used the climate change velocity since the last glacial maximum (~21,000 years ago), obtained from Sandel *et al.* [27]. This variable represents a measure of the speed at which climatic conditions change in a particular region [67], obtained by dividing a temporal gradient (in this case, the differences in annual mean temperature between the current and the last glacial maximum) by a spatial gradient (interpreted as the rate of temperature change relative to the distance between adjacent cells), resulting in a variable expressed in units of distance per time. All the aforementioned variables were transformed to an "Albers Equal Area" projection using the "projectRaster" function from the "raster" package [68] and obtained at a spatial resolution of ~340 km^2, except for the climate change velocity, which was obtained at a resolution of 2.5 arc-minutes (~21.6 km^2). However, in order to match the presence-absence matrix described in section 2.5 below, these variables were resampled to a resolution of 50 km × 50 km using the "resample" function from the "raster" package [68].

2.3 Phylogenetic relationships

To represent the phylogenetic relationships among species, we considered two different phylogenies. These phylogenies are the most recent and comprehensive phylogenies that include the genus *Sceloporus*. The first phylogeny was constructed by Leache *et al.* [35] and includes 129 phrynosomatid species, of which 86 belong to the 116 described species for the genus [33]. This phylogeny includes six subspecies of *Sceloporus* (*S. magister*

cephaloflavus, S. magister magister, S. magister uniformis, S. formosus scitulus, S. grammicus microlepidotus and *S. megalepidurus pictus*), and two species without current taxonomic validity: *S. vandenburgianus*, currently considered as a subspecies of *S. graciosus* [69] and *S. lineolateralis*, at present considered as a subspecies of *S. jarrovi* [70,71]. These latter species, as well as the aforementioned subspecies, were removed from the phylogeny to match with the most recent taxonomic update [33]. The second phylogeny was the one constructed for the order Squamata by Tonini *et al.* [36], including 9,754 species, of which 97 belonged to *Sceloporus*. Both phylogenies were constructed using a Bayesian approach, generating 10,000 phylogenetic trees, and were calibrated in time using fossil records. From the 10,000 trees obtained in each study, the maximum clade credibility tree was derived based on the median branch lengths, using the software TreeAnnotator v1.10.4 [72]. Additionally, to determine if the species values for each predictor variable are shared among closely related species, we calculated phylogenetic signals using conventional metrics such as Blomberg's K and Pagel's lambda with the "phylosig" function from the "phytools" package [73].

2.4 Cross-species and assemblage level approaches

For the following analyses, we considered three datasets: 1) all species, 2) those contained in Leache *et al.* [35] and 3) the ones present in Tonini *et al.* [36]. We include only those species for which we were able to estimate their geographic ranges. Thus, of the 116 species reported for the genus [33], in the subset of "all species", we had 103 species, while in subsets of Leache *et al.* [35] and Tonini *et al.* [36], there were 81 and 92 species, respectively. First, we constructed a presence-absence matrix with a resolution of 50 × 50

km and an “Albers Equal Area” projection using the “lets.presab” function from the “letsR” package [74].

To robustly evaluate Rapoport’s rule we considered two complementary levels of analyses considering the two dimensions of the pattern: at the cross-species level and the assemblage level [75]. At the cross-species level, we obtained the range size in km^2 (response variable) and latitudinal midpoint (explanatory variable) for each species from the presence-absence matrix. These attributes were calculated using the “lets.rangesize” and “lets.midpoint” functions respectively, both implemented in the “letsR” package [74]. Given the large variation in magnitude orders for the range sizes, this variable was log-transformed. To ensure that the estimation of these properties was not dependent on the method used to construct the geographic ranges, we compared their range sizes and latitudinal midpoints using Spearman’s rank correlation coefficient. We found that three methods were highly correlated (S1 Fig), so we only used the method that demonstrated more conservative estimates of range size (alpha-hulls) for downstream analyses. At the assemblage level, our response variable was defined as the median of the log-transformed range size for all the species present in a given cell. To perform this calculation, we employed the “lets.maplizer” function [74]. Meanwhile, our explanatory variable was the latitudinal centroid corresponding to each cell in the presence-absence matrix.

To evaluate the environmental hypotheses, we used the same response variable as in the evaluation of Rapoport’s rule, log-range size of species and median log-range size of assemblages for the cross-species and assemblage level analyses, respectively. For the explanatory variables, we considered two distinct approaches, depending on the level of analysis: For the cross-species level we considered the mean climatic values within the

geographic range of each species. For the assemblage level, we focused on the absolute values within each cell.

2.5 Statistical analysis

All predictor and response variables were standardized using the “scale” function from R base. The mean was set to 0 and the variance was set to 1. We also checked for multicollinearity problems using the “vif” function from the “usdm” package [61].

2.5.1 Ordinary least squares

We explored the validity of Rapoport’s rule and evaluated the environmental hypotheses using ordinary least squares (OLS) models. Unlike Rapoport’s rule validation, where we used univariate regressions, for environmental hypothesis we performed multiple regressions because they allowed us to consider the effect of all explanatory variables within the model [76]. To do this, we employed a multi-model inference approach based on information theory [77]. We constructed multiple models that considered all possible combinations of our predictor variables using the “dredge” function from the “MuMIn” package [78]. The relative support for each model was determined based on their Akaike weights. At the cross-species level, we found limited support for most variable combinations (S1 Appendix). Therefore, we performed coefficient averaging using the 95% of the Akaike weights as confidence set [77]. This was performed using the “model.avg” function from the “MuMIn” package [78]. At the assemblage level, the best model consistently included all predictors and had strong support (high Akaike weights, S1 Appendix). Thus, model averaging was not performed.

2.5.2 Phylogenetic generalised least squares

When analyzing relationships at the cross-species level, we need to consider the phylogenetic autocorrelation among species. This is because phylogenetic autocorrelation violates the assumption of independence among observations [79]. To avoid this issue, we employed the Phylogenetic generalised least squares (PGLS) method to incorporate phylogenetic covariance within our regressions [80]. To do so, we used the "pgls" function from the "caper" package [81]. The same model selection and averaging procedure described for OLS was also performed for PGLS models.

2.5.3 Simultaneous autoregressive models

Due to the potential presence of spatial autocorrelation in the assemblage level approach, we performed simultaneous autoregressive (SARs) models using the "errorsarlm" function from the "spatialreg" package [82]. These models are used to incorporate spatial autocorrelation in statistical analyses, using neighborhood matrices to define the relationship between the model residuals at each location and those of neighboring locations [83]. For the environmental hypotheses, we selected the model with the best support based on OLS (which considered all predictor variables). In order to determine the most appropriate spatial weights matrix, we followed a similar protocol to the one used in Skeels *et al.* [84]. For this, we constructed four different models (one OLS and three SARs models). The neighborhood distance matrix used was 50 km (distance between the centroids of the cells). Three schemes were applied for this distance matrix [85]: One row standardized (W), another globally standardized (C), and a variance stabilizing (S). The selection of the best SAR model was based on comparing the lowest values of the Akaike Information Criterion and the highest values of R^2 (S2 Appendix).

2.6 Null models

To compare our results against expected relationships, we created a series of null models. These null models assumed that the spatial distribution of range size was not influenced by environmental gradients [9]. Our null hypothesis was based on cohesive range models using the spreading-dye algorithm [86]. Using the previously generated presence-absence matrix, we applied the “rangemod2d” function from the “RangemodelR” package to randomize the position of species ranges while preserving their size [30]. This function is based on the simulation implemented in Rahbek *et al.* [87], where for each species, an initial site is randomly selected, and then neighboring cells are added until the number of occupied grid-cells for the species is reached. This procedure was repeated 100 times. Then, we calculated the expected regression coefficients (slopes) for OLS, PGLS, and SARs using the procedure described for the observed data. Null frequency distributions were generated from the 100 simulated coefficients to compare with the observed coefficients. We considered the observed coefficients to be significantly different from the null coefficients if they fell outside the 95% confidence interval of their frequency distributions. Due to the directional nature of our hypotheses, we decided to use a one-tailed test. Thus, for those associated with negative predictions, we opted for the 0.05 percentile from the null distribution to establish the cutoff threshold. Conversely, for hypotheses linked to positive predictions, we utilized the 0.95 percentile.

3. Results

3.1 Cross-species level analysis

At the cross-species level, we detected a higher frequency of species with small range sizes creating a left-skewed distribution and a log-normal distribution was observed when the range size was log-transformed (Fig 1). The range sizes varied by up to four orders of magnitude. The smallest range sizes were 6,160.5 km^2 for *S. esperanzae* and *S. macdougalli*, and the largest range size was 5,436,641.25 km^2 for *S. undulatus* (S1 Table).

Figure 1. Frequency distribution of range size in km^2 (left) and natural logarithm of range size (right) at cross-species level across all datasets.

Using Pagel's lambda, significant phylogenetic signals were observed for all attributes except for range size (λ = 0 and p = 1) regardless of the phylogeny used. Similarly, Blomberg's K analysis revealed phylogenetic signals for all attributes except for range size and climate change velocity in both phylogenies (Table 2).

Table 2. Phylogenetic signal for all the attributes across Leache *et al.* [35] Tonini *et al.* [36] datasets. Bold values indicate significant results.

Trait	K	p-value	λ	p-value	Phylogeny
Latitudinal Midpoint	**0.46**	**< 0.01**	**0.79**	**< 0.01**	Tonini
log (Range size)	0.24	0.09	0	1	Tonini
Climate change velocity	0.23	0.32	**0.35**	**< 0.05**	Tonini
Elevation	**0.26**	**< 0.05**	**0.38**	**< 0.05**	Tonini
Minimum temperature of the coldest month	**0.35**	**< 0.01**	**0.66**	**< 0.01**	Tonini
Temperature annual range	**0.38**	**< 0.01**	**0.70**	**< 0.01**	Tonini

Latitudinal Midpoint	**0.77**	**< 0.01**	**0.98**	**< 0.01**	Leache
log (Range size)	0.28	0.37	0	1	Leache
Climate change velocity	0.37	0.10	**0.56**	**< 0.01**	Leache
Elevation	**0.49**	**< 0.01**	**0.70**	**< 0.01**	Leache
Minimum temperature of the coldest month	**0.63**	**< 0.01**	**0.91**	**< 0.01**	Leache
Temperature annual range	**0.71**	**< 0.01**	**0.98**	**< 0.01**	Leache

Our results indicated support for Rapoport's rule in all three datasets (Fig 2, Table 3), confirming a positive relationship between range size and latitude (Slope = 0.5 - 0.53, $R^2 = 0.24 - 0.27$, $p < 0.01$). The results for OLS and PGLS were very similar, and no phylogenetic signal was observed for the range size-latitude relationship in any dataset ($\lambda = 0$, Table 3). Regarding the climatic hypotheses, the model including only minimum temperature of the coldest month and elevation was selected as the best model, although it had low support (Akaike weights = 0.17 - 0.20; S1 Appendix). Still, the higher importance of these two variables, compared to the other ones, was confirmed in the model averaging approach, where the climatic extreme hypothesis consistently had the stronger relationship with range size (Slope = -0.45 to -0.33) followed by elevation (Slope = -0.14 to -0.18). Given this model averaging, the other hypotheses were also considered with positive relationships with climate change velocity (Slope = 0.13 - 0.16) and climatic variability (Slope = 0.04 - 0.17), although these had less relevance in explaining range size variation across space (Table 3).

Figure 2. Linear regressions for Rapoport's rule at the cross-species level

Table 3. OLS and PGLS slopes for Rapoport's rule and climatic hypothesis (model averaging) at the cross-species level

	All species	Tonini et al. 2016		Leache et al. 2016	
	**OLS**	**OLS**	**PGLS**	**OLS**	**PGLS**
			**Rapoport's rule**		
Slope	0.53	0.52	0.52	0.50	0.50
R^2	0.27	0.26	0.26	0.24	0.24
λ	-	-	0	-	0
p-value	< 0.01	< 0.01	< 0.01	< 0.01	< 0.01
			**Climatic hypothesis**		
CEH min	-0.45	-0.33	-0.33	-0.36	-0.36
Elevation	-0.18	-0.16	-0.16	-0.14	-0.14
CCV	0.15	0.13	0.13	0.16	0.16
CVH	0.04	0.17	0.17	0.08	0.08

3.2 Assemblage level analysis

At the assemblage level, our results also provided support for Rapoport's rule regardless of the analyzed dataset (Fig 3, Table 4), with positive slopes in the relationship between range size and latitude (Slope = 0.68 - 0.72, R^2 = 0.5 - 0.96, p = 0). In general, SARs had higher R^2 values (0.96 in all three datasets) compared to OLS (0.5 - 0.51). The slopes were similar between OLS and SARs, with SARs having slightly lower values (Slope = 0.68 - 0.70, Table 4). The climatic extreme hypothesis was the most relevant in explaining the variation in range size across all datasets ($Slope_{OLS}$ = -0.85 to -0.80 and $Slope_{SARs}$ = -0.35 to -0.30) followed by elevation ($Slope_{OLS}$ = -0.42 to -0.43 and $Slope_{SARs}$ = -0.14 to -0.12) and historical climate stability hypothesis ($Slope_{OLS}$ = 0.21 - 0.22, $Slope_{SARs}$ = 0.02). The hypothesis of climatic variability was not supported at the assemblage level, as we observed low negative slopes (Table 4).

Figure 3. Linear regressions for Rapoport's rule at the assemblage level

Table 4. OLS and SARs slopes for Rapoport's rule and climatic hypothesis (best supported model) at the assemblage level

	All species		Tonini et al. 2016		Leache et al. 2016	
	**OLS**	**SAR**	**OLS**	**SAR**	**OLS**	**SAR**
			Rapoport's rule			
Slope	0.72	0.68	0.71	0.68	0.71	0.70
R^2	0.51	0.96	0.50	0.96	0.50	0.96
p-value	0.00	0.00	0.00	0.00	0.00	0.00
			Climatic hypothesis			
CEH min	-0.84	-0.31	-0.85	-0.30	-0.80	-0.35
Elevation	-0.43	-0.14	-0.42	-0.12	-0.42	-0.13
CCV	0.21	0.02	0.22	0.02	0.22	0.02
CVH	-0.08	-0.08	-0.09	-0.07	-0.05	-0.05

3.2 Null models

Regarding the null models, for Rapoport's rule, we found significant differences between the observed coefficient and the simulated ones at both the cross-species and assemblage levels in all datasets, regardless of the statistical method used to analyze the relationship (S2 Fig and Fig 4). Concerning the climatic hypotheses, for the OLS models (S2 Fig), the observed coefficients for climate extremes and elevation hypotheses were significantly different from the null coefficients in all datasets, both at the cross-species and assemblage levels. On the other hand, the observed slopes for the historical climate stability hypothesis and climate variability hypothesis did not differ from the null values in any dataset or level of analysis (S2 Fig). For PGLS, only the climate extremes and elevation hypotheses significantly differed from the null values (Fig 4). Regarding SARs, we did not find significant differences between the observed and simulated coefficients for any of the

climatic hypotheses (Fig 4). The individual coefficients of each simulated regression model can be found in S3 and S4 Appendixes.

Figure 4. Null frequency distributions of the 100 SARs and PGLS. Red dashed line = Observed value. Blue dashed lines = 0.05% and 95% interval confidence of the null distribution. CEH = Climate extremes hypothesis, EH = Elevation hypothesis, HCSH = Historical climate stability hypothesis, CVH = Climate variability hypothesis.

4. Discussion

In this study, we evaluated the Rapoport's rule in spiny lizards (genus *Sceloporus*) and assessed the support and importance of multiple environmental hypotheses to explain their latitudinal range size variation. Our results confirmed a positive relationship between range size and latitude, providing support for Rapoport's rule. This finding is consistent with previous studies supporting the validity for this rule in the analyzed region [6,8,11,88], as well as in other reptile groups such as snakes [20,89]. However, studies evaluating Rapoport's rule in lizards are still limited. Cruz *et al.* [16] found a positive relationship between range size and latitude when analyzing a small sample of lizards belonging to the *Liolaemus* genus. Conversely, Pincheira-Donoso [17], observed an inverse pattern to that predicted by Rapoport's rule for the same genus when analyzing a larger number of species. Moreover, Pintor *et al.* [90] found mixed results when analyzing Rapoport's rule in three different clades of lizards in Australia (*Carlia*, *Ctenotus* y *Egernia*). These findings emphasize that different patterns can emerge when analyzing different clades or number of

species [16]. Despite this, our results were consistent regardless of our analyzed datasets thus supporting the validity of Rapoport's rule in the *Sceloporus* genus.

Our confirmation for the existence of Rapoport's rule was also consistent both at the cross-species and assemblage levels, as has been found in other taxa [8,20,89,90]. This happens because both approaches are grounded in the same underlying principle, where the assemblage of species emerges from the overlap of their individual distributions [91]. Therefore, when the majority of species with large range sizes are found at temperate latitudes, the overlapping of their distributions lead to those locations having, on average, larger range sizes compared to tropical regions.

Concerning the evaluated environmental hypotheses, our results showed consistency between the cross-species and the assemblage level analyses. Overall, the climate extreme hypothesis consistently had the highest support, in line with findings from previous studies conducted in both plants and marine and terrestrial animals [24,92,93]. This hypothesis suggests that species capable of tolerating more extreme temperature limits, namely cold ones, are able to expand the boundaries of their geographic ranges [24]. In accordance with this, studies conducted in *Sceloporus*, found that species that tolerate colder temperatures tend to have larger geographic ranges [22]. Likewise, studies conducted on other genus of lizards such as *Liolaemus* showed that cold climate species exhibit greater physiological plasticity and have larger geographic ranges [21]. Within *Sceloporus*, species capable of tolerating low temperatures, such as those found in temperate regions, exhibit greater efficiency in thermoregulation, maintaining their body temperature even in cold climates by employing ethological strategies like limiting their activity season through hibernation [94]. Both characteristics make it easier for them to spread in colder climates.

These observations are closely related to elevation which was another important factor in determining the spatial variation of *Sceloporus* range sizes. According to the seminal hypothesis proposed by Janzen [28], mountains in tropical regions represent a more restrictive physiological barrier than in temperate regions. If this mechanism is operating to limit range size, species in these regions should exhibit high genetic differentiation among their populations, speciation through allopatry, and high species richness in tropical mountain regions [95]. Indeed, for the genus *Sceloporus*, there is evidence suggesting that these three processes may be occurring across mountain regions. Grummer *et al.* [96] observed limited genetic flow and genetic differentiation among populations of mountain-dwelling species within the *Sceloporus spinosus* group. Furthermore, allopatric speciation has been previously suggested as one of the most plausible explanations for the diversification of this lizards [43,97]. Additionally, it has been observed for this genus that tropical regions with high topographic variation are associated with high species richness [43]. Likewise, it has been observed that *Sceloporus* species in temperate regions are able to maintain their body temperature regardless elevation, contrasting with tropical zones where mountain species differ in body temperature compared to lowland species [94]. Previously, in other groups of lizards (families Scincidae and Varanidae), it was also observed that elevation had a negative effect on species' dispersal ability [23], and high-elevation areas are associated with low genetic diversity and small population sizes [98]. Additional studies have also found that elevation affects latitudinal patterns of range size in other ectotherm taxa [15,18].

Similarly, we also found support for the remaining hypotheses, but with slight relevance in explaining the observed spatial pattern in range size. Regarding the climate variability hypothesis, our results contrast with the original proposal of Stevens [5], who

suggested this hypothesis as one of the main explanations for Rapoport's rule. Although it has received broad support in explaining spatial variation in range size in different groups of reptiles such as snakes [20] and some lizard clades [16,89], other studies have rejected this hypothesis as the primary determinant of Rapoport's rule even for the same lizard clades (*Liolaemus*) [21,99]. In particular for *Sceloporus* lizards, the lack of support for the climate variability hypotheses may be due to their ability to maintain their body temperatures across seasons throughout the year, thus not being affected by such temperature variability in determining their movements and thus their range sizes [100].

We also observed that climate change velocity received statistical support but had little power to explain Rapoport's rule for our lizard genus. One of the main drivers behind this hypothesis is the extinction of small-ranged species at northern latitudes due to climatic instability [27]. Thus, our results suggests that extinction processes may not have been a significant factor in range size dynamics of *Sceloporus* lizards. Indeed, for this genus, low and stable extinction rates have been consistently observed throughout its evolutionary history [35]. Moreover, sites with the highest species diversity for the genus [43] coincide with regions where past climate variation has been relatively stable, such as the center of Mexico [27].

Although some environmental variables were more important than others in explaining the range size spatial pattern of *Sceloporus*, all variables contributed to our final models. First, this implies that no single hypotheses can solely explain the causes behind Rapoport's rule in this lizard clade. Still, among the evaluated hypotheses, some had more support (climate extremes and elevation) than others, which allowed us to infer the most important drivers of this ecogeographical rule in our studied group. This was also consistent between cross-species and assemblage levels, where averaged model and best

model, respectively, showed higher importance of minimum temperature of the coldest month and elevation. Models for both levels exhibited a good fit to the data, explaining a significant amount of variance of the range size spatial pattern. Considering our model selection approach, this is relevant because it provides support to our inferences beyond simply relying on a best model that could perform better than a set of candidates but still provide poor explanatory power [101,102].

Our analyses also revealed a correspondence between phylogenetically related species and their geographic and environmental proximity. Accordingly, our results showed phylogenetic signal in the location of species (i.e., their latitudinal midpoint) as well as in their occupied environments as shown by most of our evaluated variables. However, range size did not show phylogenetic signal, which supports the idea that speciation within *Sceloporus* primarily took place through allopatric processes [43,97], since this event can disrupt the preservation of range size while still keeping species in close environmental and geographic proximity [103]. This finding provides a clear explanation for the lack of phylogenetic signal in the relationship between range size and both latitude and climate.

When we contrasted our results with null scenarios, we discovered that the spatial variation of range size for the genus *Sceloporus* differs significantly from null expectations regardless of the level of analysis. This finding indicates that our observed patterns cannot be replicated by randomizing the location of species' ranges under the assumption of no environmental control. Therefore, other factors more than geometric restrictions should be influencing their spatial variation in range size. However, for the environmental hypothesis, we observed differences between the levels of analysis. At the cross-species level, for climate extremes we observed significant deviations from what was expected based on the null model. This can be explained by the fact that the study region exhibits a pronounced

latitudinal temperature gradient, with colder areas in the temperate region [58]. Therefore, randomizing the geographic position will not necessarily replicate the relationship found in the observed data. For instance, it is possible that small range size values are randomly placed in extremely cold locations, which would generate different relationships than the observed pattern. Likewise, we confirm that the relationship between range size and elevation follows a non-random pattern. The presence of this signal can be explained by the fact that, in tropical areas, species face greater difficulty in migrating between regions with different elevations [28,95]. On the contrary, despite the presence of diverse mountain ranges in the northern latitudes of our study region [104], species distributions in these areas also extend across the vast plains of North America. This occurrence results in their distribution having, on average, sites of intermediate elevation, distinguishing their tropical counterparts. Nevertheless, null models have the capability to position species (regardless of their range size) in regions of high or low elevation indiscriminately, resulting in relationships that diverge from those observed in the real data.

Conversely, the observed relationships for climate variability hypothesis and climate change velocity were not significantly different from the null expectations. This result makes sense due to the low contribution of these variables in explaining range size, so it is not surprising that their coefficients can be replicated by our simulations. At the assemblage level, our analyses did not reveal significant differences compared to the null models for any of the studied hypotheses. Previous studies have emphasized that spatial autocorrelation can produce significant relationships between climate and range size [31,105]. Indeed, these relationships can originate from the inherent spatial structure of climate variables and species' attributes at the assemblage level, such as range size [106,107]. This latter phenomenon arises because the range size of an assemblage emerges

from the overlapping of individual species, leading to issues of pseudo-replication [106]. Additionally, when combined with the pronounced spatial structure present in climate variables, these factors can contribute to the emergence of strong associations that lack biological meaning. Moreover, it is also possible that the overlap of species' ranges may be influenced by other factors beyond climate, such as biotic interactions [105]. Thus, while we observed a strong association with certain environmental variables and range size at the assemblage level, we were unable to distinguish such association from the effect of geometric constraints and inherent spatial structure. Still, pseudo-replication issues and spatial structure are less important at the cross-species level, which supports our overall findings and emphasizes the need to consider both levels of analyses for a robust evaluation of ecogeographical patterns [75].

5. Conclusions

We found support for Rapoport's rule at both the cross-species and assemblage levels in *Sceloporus*. Even though our region of analysis has strong geometric restrictions in landmasses, which would make it prone to exhibiting Rapoport's rule, our null models, which are useful for detecting these effects, revealed significant differences between the observed and simulated relationships. This indicates that the Rapoport's rule confirmed in our study is not a consequence of such constraints. In general, the climate extremes and elevation hypotheses had the highest support. At the cross-species level, these observations were significantly different from what was expected by null simulations, suggesting that this effect is linked to the physiology of the organisms. At the assemblage level, we did not observe significant differences from the null models for any climatic hypothesis, showing a

strong effect of spatial structure in shaping these relationships or the possible influence of other factors affecting the species assemblage.

Acknowledgments

To the Consejo Nacional de Humanidades Ciencias y Tecnologías (CONAHCYT) for providing me with the necessary support to carry out this research (CVU: 1010702). To the Posgrado en Ciencias Biológicas (PCB) at the Universidad Nacional Autónoma de México (UNAM), as this work is a derivative of my doctoral research in this program, and this article accomplishes one of the requirements for academic degree. To Dr. Luis Daniel Ávila Cabadilla and Dr. Enrique Martinez Meyer for comments and discussions that help improve this work. Finally, to the members of the evolutionary macroecology lab (https://maevolab.mx) for the support in phylogenetic and statistical analysis during this research.

References

1. Gaston KJ. How large is a species' geographic range? Oikos. 1991; 434–438.
2. Mota-Vargas C, Rojas-Soto OR. The importance of defining the geographic distribution of species for conservation: The case of the Bearded Wood-Partridge. Journal for Nature Conservation. 2012;20: 10–17.
3. Guo Q, Qian H, Zhang J. On the relationship between species diversity and range size. Journal of Biogeography. 2022;49: 1911–1919.
4. Davies TJ, Purvis A, Gittleman JL. Quaternary climate change and the geographic ranges of mammals. The American Naturalist. 2009;174: 297–307.
5. Stevens GC. The latitudinal gradient in geographical range: how so many species coexist in the tropics. The American Naturalist. 1989;133: 240–256.

6. Arita HT, Rodríguez P, Vázquez- Domínguez E. Continental and regional ranges of North American mammals: Rapoport's rule in real and null worlds. Journal of Biogeography. 2005;32: 961–971.

7. Chen A, Li Z, Zheng Y, Zhan J, Yang B, Yang Z. Decreasing Species Richness with Increase in Elevation and Positive Rapoport Effects of Crambidae (Lepidoptera) on Mount Taibai. Insects. 2022;13: 1125.

8. Dyer EE, Redding DW, Cassey P, Collen B, Blackburn TM. Evidence for Rapoport's rule and latitudinal patterns in the global distribution and diversity of alien bird species. Journal of Biogeography. 2020;47: 1362–1372.

9. Colwell RK, Hurtt GC. Nonbiological gradients in species richness and a spurious Rapoport effect. The American Naturalist. 1994;144: 570–595.

10. Gaston KJ, Blackburn TM, Spicer JI. Rapoport's rule: time for an epitaph? Trends in Ecology & Evolution. 1998;13: 70–74.

11. Ruggiero A, Werenkraut V. One- dimensional analyses of Rapoport's rule reviewed through meta- analysis. Global Ecology and Biogeography. 2007;16: 401–414.

12. Acharya BK, Sanders NJ, Vijayan L, Chettri B. Elevational gradients in bird diversity in the Eastern Himalaya: an evaluation of distribution patterns and their underlying mechanisms. PloS one. 2011;6: e29097.

13. Kim J-Y, Seo C, Hong S, Lee S, Eo SH. Altitudinal range-size distribution of breeding birds and environmental factors for the determination of species richness: An empirical test of altitudinal Rapoport's rule and non-directional rescue effect on a local scale. Plos one. 2019;14: e0203511.

14. Luo Z, Tang S, Li C, Chen J, Fang H, Jiang Z. Do Rapoport's rule, mid-domain effect or environmental factors predict latitudinal range size patterns of terrestrial mammals in China? PLoS One. 2011;6: e27975.

15. Whitton FJ, Purvis A, Orme CDL, Olalla- Tárraga MÁ. Understanding global patterns in amphibian geographic range size: does Rapoport rule? Global Ecology and Biogeography. 2012;21: 179–190.

16. Cruz F, Fitzgerald L, Espinoza R, Schulte Ii J. The importance of phylogenetic scale in tests of Bergmann's and Rapoport's rules: lessons from a clade of South American lizards. Journal of evolutionary biology. 2005;18: 1559–1574.

17. Pincheira-Donoso D. Predictable variation of range-sizes across an extreme environmental gradient in a lizard adaptive radiation: evolutionary and ecological inferences. PLoS One. 2011;6: e28942.

18. Grünig M, Beerli N, Ballesteros- Mejia L, Kitching IJ, Beck J. How climatic variability is linked to the spatial distribution of range sizes: seasonality versus

climate change velocity in sphingid moths. Journal of Biogeography. 2017;44: 2441–2450.

19. Morueta- Holme N, Enquist BJ, McGill BJ, Boyle B, Jørgensen PM, Ott JE, et al. Habitat area and climate stability determine geographical variation in plant species range sizes. Ecology letters. 2013;16: 1446–1454.

20. Ten Caten C, Lima- Ribeiro MS, Yañez- Arenas C, Villalobos F, Díaz- Gamboa L, Terribile LC. Robustness of Bergmann's and Rapoport's rules to different geographical range estimates in New World pit vipers. Journal of Biogeography. 2023;50: 365–379.

21. Bonino MF, Azócar DLM, Schulte II JA, Abdala CS, Cruz FB. Thermal sensitivity of cold climate lizards and the importance of distributional ranges. Zoology. 2015;118: 281–290.

22. Buckley LB. The range implications of lizard traits in changing environments. Global Ecology and Biogeography. 2010;19: 452–464.

23. Smissen PJ, Melville J, Sumner J, Jessop TS. Mountain barriers and river conduits: phylogeographical structure in a large, mobile lizard (Varanidae: Varanus varius) from eastern Australia. Journal of Biogeography. 2013;40: 1729–1740.

24. Pither J. Climate tolerance and interspecific variation in geographic range size. Proceedings of the Royal Society of London Series B: Biological Sciences. 2003;270: 475–481.

25. Dynesius M, Jansson R. Evolutionary consequences of changes in species' geographical distributions driven by Milankovitch climate oscillations. Proceedings of the National Academy of Sciences. 2000;97: 9115–9120.

26. Jansson R. Global patterns in endemism explained by past climatic change. Proceedings of the Royal Society of London Series B: Biological Sciences. 2003;270: 583–590.

27. Sandel B, Arge L, Dalsgaard B, Davies R, Gaston K, Sutherland W, et al. The influence of Late Quaternary climate-change velocity on species endemism. Science. 2011;334: 660–664.

28. Janzen DH. Why mountain passes are higher in the tropics. The American Naturalist. 1967;101: 233–249.

29. Colwell RK, Lees DC. The mid-domain effect: geometric constraints on the geography of species richness. Trends in ecology & evolution. 2000;15: 70–76.

30. Marathe A. rangemodelR: mid-domain effect and species richness. R package Version. 2019;1.

31. Moore TE, Bagchi R, Aiello- Lammens ME, Schlichting CD. Spatial autocorrelation inflates niche breadth–range size relationships. Global Ecology and Biogeography. 2018;27: 1426–1436.

32. Šizling AL, Storch D, Keil P. Rapoport's rule, species tolerances, and the latitudinal diversity gradient: geometric considerations. Ecology. 2009;90: 3575–3586.

33. Uetz P, Koo MS, Aguilar R, Brings E, Catenazzi A, Chang AT, et al. A quarter century of reptile and amphibian databases. Herpetol Rev. 2021;52: 246–255.

34. Lawing AM, Polly PD, Hews DK, Martins EP. Including fossils in phylogenetic climate reconstructions: A deep time perspective on the climatic niche evolution and diversification of spiny lizards (Sceloporus). The American Naturalist. 2016;188: 133–148.

35. Leaché AD, Banbury BL, Linkem CW, de Oca AN-M. Phylogenomics of a rapid radiation: is chromosomal evolution linked to increased diversification in north american spiny lizards (Genus Sceloporus)? BMC evolutionary biology. 2016;16: 1–16.

36. Tonini JFR, Beard KH, Ferreira RB, Jetz W, Pyron RA. Fully-sampled phylogenies of squamates reveal evolutionary patterns in threat status. Biological Conservation. 2016;204: 23–31.

37. Cruz-Elizalde R, Ramírez-Bautista A, de Cáceres-González FFN. Sexual dimorphism and feeding ecology of the black-bellied bunchgrass Lizard Sceloporus aeneus (Squamata: Phrynosomatidae) in Central Mexico. South American Journal of Herpetology. 2020;18: 46–55.

38. Díaz de la Vega-Pérez A, Barrios-Montiel R, Jiménez-Arcos V, Bautista A, Bastiaans E. High-mountain altitudinal gradient influences thermal ecology of the Mesquite Lizard (Sceloporus grammicus). Canadian Journal of Zoology. 2019;97: 659–668.

39. Gilbert AL, Lattanzio MS. Ontogenetic variation in the thermal biology of yarrow's spiny lizard, sceloporus jarrovii. PloS one. 2016;11: e0146904.

40. Lambert SM, Wiens JJ. Evolution of viviparity: a phylogenetic test of the cold-climate hypothesis in phrynosomatid lizards. Evolution. 2013;67: 2614–2630.

41. Martínez-Méndez N, Mejía O, Ortega J, Méndez-de la Cruz F. Climatic niche evolution in the viviparous Sceloporus torquatus group (Squamata: Phrynosomatidae). PeerJ. 2019;6: e6192.

42. Rivera JA, Lawing AM, Martins EP. Reconstructing historical shifts in suitable habitat of Sceloporus lineages using phylogenetic niche modelling. Journal of Biogeography. 2020;47: 2117–2128.

43. Rivera JA, Rich HN, Michelle Lawing A, Rosenberg MS, Martins EP. Occurrence data uncover patterns of allopatric divergence and interspecies interactions in the evolutionary history of Sceloporus lizards. Ecology and Evolution. 2021;11: 2796–2813.

44. Wiens JJ, Kozak KH, Silva N. Diversity and niche evolution along aridity gradients in North American lizards (Phrynosomatidae). Evolution. 2013;67: 1715–1728.

45. R Core Team. R: A language and environment for statistical computing. R Foundation for Statistical Computing, Vienna, Austria. URL https://www.R-project.org/. 2022.

46. GBIF. GBIF Occurrence Download. 2023. doi:10.15468/dl.5234bm

47. Naturalista. Downloaded on March 10. 2023.

48. Osorio- Olvera L, Lira- Noriega A, Soberón J, Peterson AT, Falconi M, Contreras-Díaz RG, et al. ntbox: An r package with graphical user interface for modelling and evaluating multidimensional ecological niches. Methods in Ecology and Evolution. 2020;11: 1199–1206.

49. Hijmans RJ, Phillips S, Leathwick J, Elith J, Hijmans MRJ. Package 'dismo.' Circles. 2017;9: 1–68.

50. Zizka A, Silvestro D, Andermann T, Azevedo J, Duarte Ritter C, Edler D, et al. CoordinateCleaner: Standardized cleaning of occurrence records from biological collection databases. Methods in Ecology and Evolution. 2019;10: 744–751.

51. Baíllo A, Chacón JE. Statistical outline of animal home ranges: an application of set estimation. Handbook of Statistics. Elsevier; 2021. pp. 3–37.

52. Edelsbrunner H, Kirkpatrick D, Seidel R. On the shape of a set of points in the plane. IEEE Transactions on information theory. 1983;29: 551–559.

53. Cobos ME, Barve V, Barve N, Jiménez-Valverde A, Nuñez-Penichet C. rangemap: An R Package to Explore Species' Geographic Ranges. Biodiversity Informatics. 2022;17.

54. Davis Rabosky AR, Cox CL, Rabosky DL, Title PO, Holmes IA, Feldman A, et al. Coral snakes predict the evolution of mimicry across New World snakes. Nature communications. 2016;7: 11484.

55. Ruby D, Dunham A. Variation in home range size along an elevational gradient in the iguanid lizard Sceloporus merriami. Oecologia. 1987;71: 473–480.

56. Phillips SJ, Anderson RP, Schapire RE. Maximum entropy modeling of species geographic distributions. Ecological modelling. 2006;190: 231–259.

57. Muscarella R, Galante PJ, Soley- Guardia M, Boria RA, Kass JM, Uriarte M, et al. ENM eval: An R package for conducting spatially independent evaluations and estimating optimal model complexity for Maxent ecological niche models. Methods in ecology and evolution. 2014;5: 1198–1205.

58. Fick SE, Hijmans RJ. WorldClim 2: new 1- km spatial resolution climate surfaces for global land areas. International journal of climatology. 2017;37: 4302–4315.

59. NASA. Shuttle Radar Topography Mission (SRTM) Global. Distributed by OpenTopography. Accessed: 2023-03-08. 2013. doi:10.5069/G9445JDF

60. Campbell LP, Luther C, Moo-Llanes D, Ramsey JM, Danis-Lozano R, Peterson AT. Climate change influences on global distributions of dengue and chikungunya virus vectors. Philosophical Transactions of the Royal Society B: Biological Sciences. 2015;370: 20140135.

61. Naimi B, Hamm NA, Groen TA, Skidmore AK, Toxopeus AG. Where is positional uncertainty a problem for species distribution modelling? Ecography. 2014;37: 191–203.

62. Cobos ME, Peterson AT, Barve N, Osorio-Olvera L. kuenm: an R package for detailed development of ecological niche models using Maxent. PeerJ. 2019;7: e6281.

63. Peterson AT, Papeş M, Soberón J. Rethinking receiver operating characteristic analysis applications in ecological niche modeling. Ecological modelling. 2008;213: 63–72.

64. Radosavljevic A, Anderson RP. Making better Maxent models of species distributions: complexity, overfitting and evaluation. Journal of biogeography. 2014;41: 629–643.

65. Soberon J, Peterson AT. Interpretation of models of fundamental ecological niches and species' distributional areas. 2005.

66. Olson DM, Dinerstein E, Wikramanayake ED, Burgess ND, Powell GV, Underwood EC, et al. Terrestrial Ecoregions of the World: A New Map of Life on EarthA new global map of terrestrial ecoregions provides an innovative tool for conserving biodiversity. BioScience. 2001;51: 933–938.

67. Loarie SR, Duffy PB, Hamilton H, Asner GP, Field CB, Ackerly DD. The velocity of climate change. Nature. 2009;462: 1052–1055.

68. Hijmans RJ, Van Etten J, Mattiuzzi M, Sumner M, Greenberg J, Lamigueiro O, et al. Raster package in R. Version https://mirro rs sjtug sjtu edu cn/cran/web/packa ges/rast e r/raster pdf. 2013.

69. Applegarth JS, Banta BH. A notable occurrence of Sceloporus graciosus vandenburgianus Cope (Reptilia: Lacertilia) in the Santa Cruz Mountains of California, with comments on its life history, ecology, taxonomy, and zoogeography. Wasmann J Biol. 1970;28: 191–198.

70. Martinez-Mendez N, Mendez-De la Cruz FR. Molecular phylogeny of the Sceloporus torquatus species-group (Squamata: Phrynosomatidae). Zootaxa. 2007;1609: 53–68.

71. Chan LM, Archie JW, Yoder AD, Fitzgerald LA. Review of the systematic status of Sceloporus arenicolus Degenhardt and Jones, 1972 with an estimate of divergence time. Zootaxa. 2013;3664: 312–320.

72. Drummond AJ, Rambaut A. BEAST: Bayesian evolutionary analysis by sampling trees. BMC evolutionary biology. 2007;7: 1–8.

73. Revell LJ. phytools: an R package for phylogenetic comparative biology (and other things). Methods in ecology and evolution. 2012; 217–223.

74. Vilela B, Villalobos F. letsR: a new R package for data handling and analysis in macroecology. Methods in Ecology and Evolution. 2015;6: 1229–1234.

75. Olalla-Tárraga MA, Bini LM, Diniz-Filho JA, Rodríguez MÁ. Cross-species and assemblage-based approaches to Bergmann's rule and the biogeography of body size in Plethodon salamanders of eastern North America. Ecography. 2010;33: 362–368.

76. Fox J. Applied regression analysis and generalized linear models. Sage Publications; 2015.

77. Johnson JB, Omland KS. Model selection in ecology and evolution. Trends in ecology & evolution. 2004;19: 101–108.

78. Barton K, Barton MK. Package 'mumin.' R package version. 2019;1.

79. Felsenstein J. Phylogenies and the comparative method. The American Naturalist. 1985;125: 1–15.

80. Freckleton RP, Harvey PH, Pagel M. Phylogenetic analysis and comparative data: a test and review of evidence. The American Naturalist. 2002.

81. Orme D, Freckleton R, Thomas G, Petzoldt T, Fritz S, Isaac N, et al. The caper package: comparative analysis of phylogenetics and evolution in R. R package version. 2013;5: 1–36.

82. Bivand R, Millo G, Piras G. A review of software for spatial econometrics in R. Mathematics. 2021;9: 1276.

83. Dormann CF, McPherson JM, Araújo MB, Bivand R, Bolliger J, Carl G, et al. Methods to account for spatial autocorrelation in the analysis of species distributional data: a review. Ecography. 2007; 609–628.

84. Skeels A, Esquerré D, Cardillo M. Alternative pathways to diversity across ecologically distinct lizard radiations. Global Ecology and Biogeography. 2020;29: 454–469.

85. Tiefelsdorf M, Griffith DA, Boots B. A variance-stabilizing coding scheme for spatial link matrices. Environment and planning A. 1999;31: 165–180.

86. Jetz W, Rahbek C. Geometric constraints explain much of the species richness pattern in African birds. Proceedings of the National Academy of Sciences. 2001;98: 5661–5666.

87. Rahbek C, Gotelli NJ, Colwell RK, Entsminger GL, Rangel TFL, Graves GR. Predicting continental-scale patterns of bird species richness with spatially explicit models. Proceedings of the Royal Society B: Biological Sciences. 2007;274: 165–174.

88. Alahuhta J, Antikainen H, Hjort J, Helm A, Heino J. Current climate overrides historical effects on species richness and range size of freshwater plants in Europe and North America. Journal of Ecology. 2020;108: 1262–1275.

89. Böhm M, Kemp R, Williams R, Davidson AD, Garcia A, McMillan KM, et al. Rapoport's rule and determinants of species range size in snakes. Diversity and Distributions. 2017;23: 1472–1481.

90. Pintor AF, Schwarzkopf L, Krockenberger AK. Rapoport's Rule: Do climatic variability gradients shape range extent? Ecological Monographs. 2015;85: 643–659.

91. Gaston KJ, Chown SL, Evans KL. Ecogeographical rules: elements of a synthesis. Journal of Biogeography. 2008;35: 483–500.

92. Pörtner HO, Bennett AF, Bozinovic F, Clarke A, Lardies MA, Lucassen M, et al. Trade-offs in thermal adaptation: the need for a molecular to ecological integration. Physiological and Biochemical Zoology. 2006;79: 295–313.

93. Bozinovic F, Calosi P, Spicer JI. Physiological correlates of geographic range in animals. Annual Review of Ecology, Evolution, and Systematics. 2011;42: 155–179.

94. Andrews RM. Geographic variation in field body temperature of Sceloporus lizards. Journal of Thermal Biology. 1998;23: 329–334.

95. Ghalambor CK, Huey RB, Martin PR, Tewksbury JJ, Wang G. Are mountain passes higher in the tropics? Janzen's hypothesis revisited. Integrative and comparative biology. 2006;46: 5–17.

96. Grummer JA, Calderón- Espinosa ML, Nieto- Montes de Oca A, Smith EN, Méndez- de la Cruz FR, Leaché AD. Estimating the temporal and spatial extent of gene flow among sympatric lizard populations (genus Sceloporus) in the southern Mexican highlands. Molecular ecology. 2015;24: 1523–1542.

97. Oufiero CE, Gartner GE, Adolph SC, Garland Jr T. Latitudinal and climatic variation in body size and dorsal scale counts in Sceloporus lizards: a phylogenetic perspective. Evolution. 2011;65: 3590–3607.

98. Dubey S, Shine R. Restricted dispersal and genetic diversity in populations of an endangered montane lizard (Eulamprus leuraensis, Scincidae). Molecular Ecology. 2010;19: 886–897.

99. Cruz FB, Azócar DLM, Bonino MF, Schulte II JA, Abdala CS, Perotti MG. Clima, distribución geográfica y viviparismo en especies de Liolaemus (Reptilia; Squamata): cuando las hipótesis se ponen a prueba. Ecosistemas. 2014;23: 37–45.

100. McGinnis SM. Sceloporus occidentalis: preferred body temperature of the western fence lizard. Science. 1966;152: 1090–1091.

101. Mac Nally R, Duncan RP, Thomson JR, Yen JD. Model selection using information criteria, but is the “best” model any good? Journal of Applied Ecology. 2018;55: 1441–1444.

102. Wagenmakers E-J, Farrell S. AIC model selection using Akaike weights. Psychonomic bulletin & review. 2004;11: 192–196.

103. Pigot AL, Phillimore AB, Owens IP, Orme CDL. The shape and temporal dynamics of phylogenetic trees arising from geographic speciation. Systematic biology. 2010;59: 660–673.

104. Snethlage MA, Geschke J, Ranipeta A, Jetz W, Yoccoz NG, Körner C, et al. A hierarchical inventory of the world’s mountains for global comparative mountain science. Scientific data. 2022;9: 149.

105. Boucher-Lalonde V, Currie DJ. Spatial autocorrelation can generate stronger correlations between range size and climatic niches than the biological signal—A demonstration using bird and mammal range maps. PloS one. 2016;11: e0166243.

106. Hawkins BA, Leroy B, Rodríguez MÁ, Singer A, Vilela B, Villalobos F, et al. Structural bias in aggregated species- level variables driven by repeated species co-occurrences: A pervasive problem in community and assemblage data. Journal of Biogeography. 2017;44: 1199–1211.

107. Saupe EE, Myers CE, Peterson AT, Soberón J, Singarayer J, Valdes P, et al. Non-random latitudinal gradients in range size and niche breadth predicted by spatial patterns of climate. Global Ecology and Biogeography. 2019;28: 928–942.

Supporting information

S1 Appendix. Results of model selection based on Akaike Information Criterion for environmental hypothesis. This table contains the raw values from all the combinations between environmental hypothesis used for model selection. (PDF).

S2 Appendix. Results of simultaneous autoregressive models. This appendix shows the results of the SARs regressions for Rapoport's rule and environmental hypothesis. (PDF).

S3 Appendix. Coefficients for null Simulations for Rapoport's rule. These tables contain all the simulated coefficients for the relation between range size and latitude. (PDF)

S4 Appendix. Coefficients for null simulations for environmental hypothesis. These tables contain all the simulated coefficients for the relation between range size and every environmental hypothesis. (PDF).

S1 Fig. Range size and Midpoints comparisons for all methods. This figure includes the comparation for range size and midpoints estimations for the three methods employed in our study (Alpha-hull, Convex-hull and SDMs). (PDF).

S2 Fig. Frequency histograms for null OLS (cross-species and assemblage level). This figure shows the 100 simulated OLS coefficients at cross-species and assemblage level (PDF).

S1 Table. Range size estimated with alpha hulls for all the species. This table contains the range size estimation for all the species through alpha-hulls**.** (PDF).

Fig1

Click here to access/download;Figure;Fig1.tiff

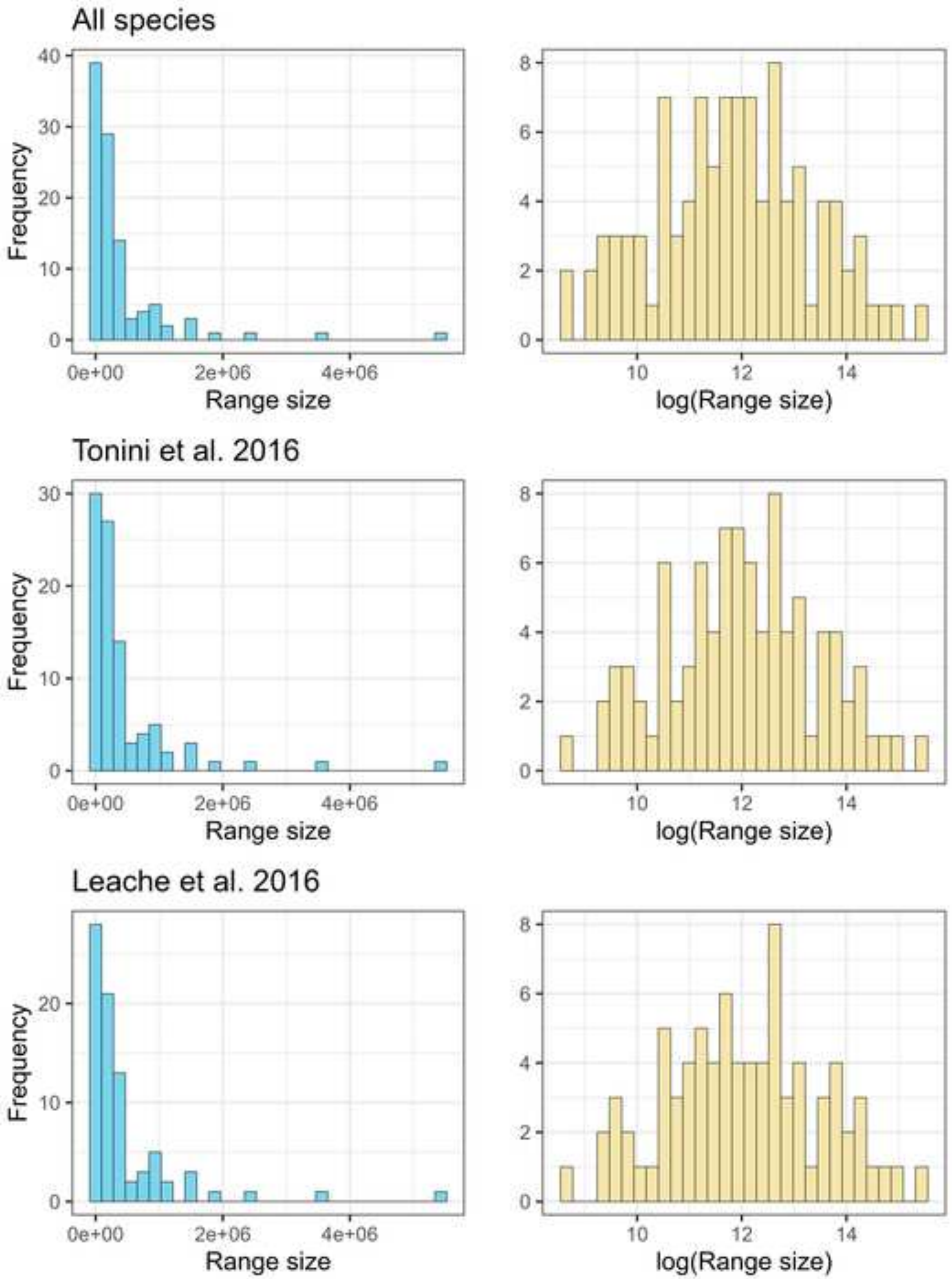

Fig2

Click here to access/download;Figure;Fig2.tiff

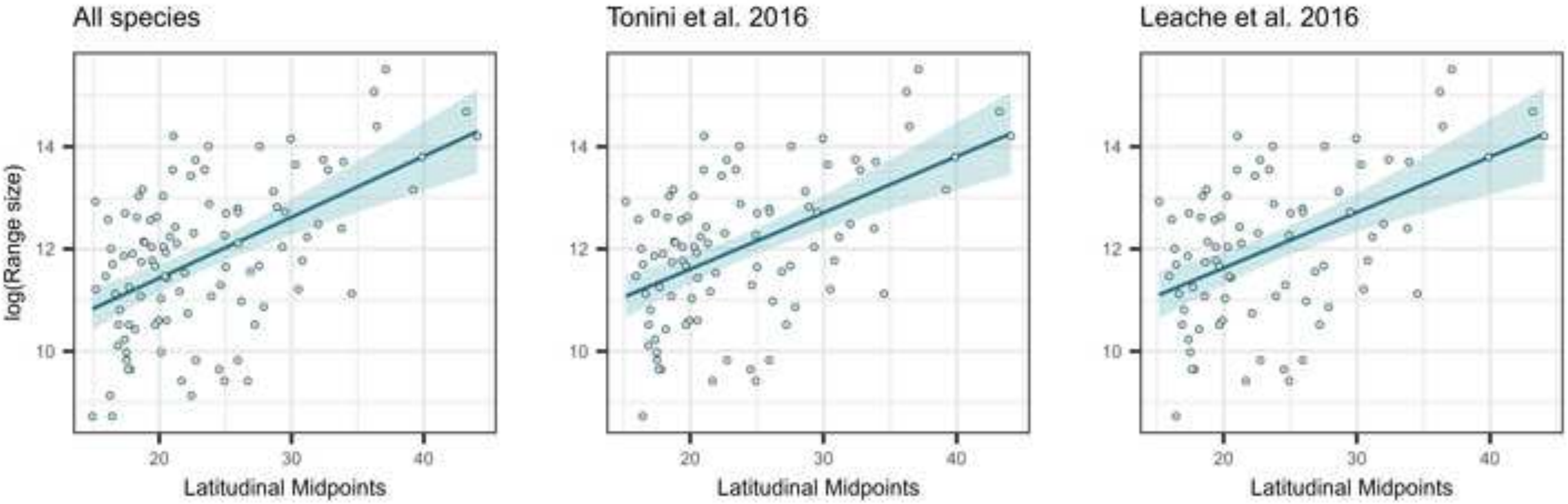

Fig3

Click here to access/download;Figure;Fig3.tiff

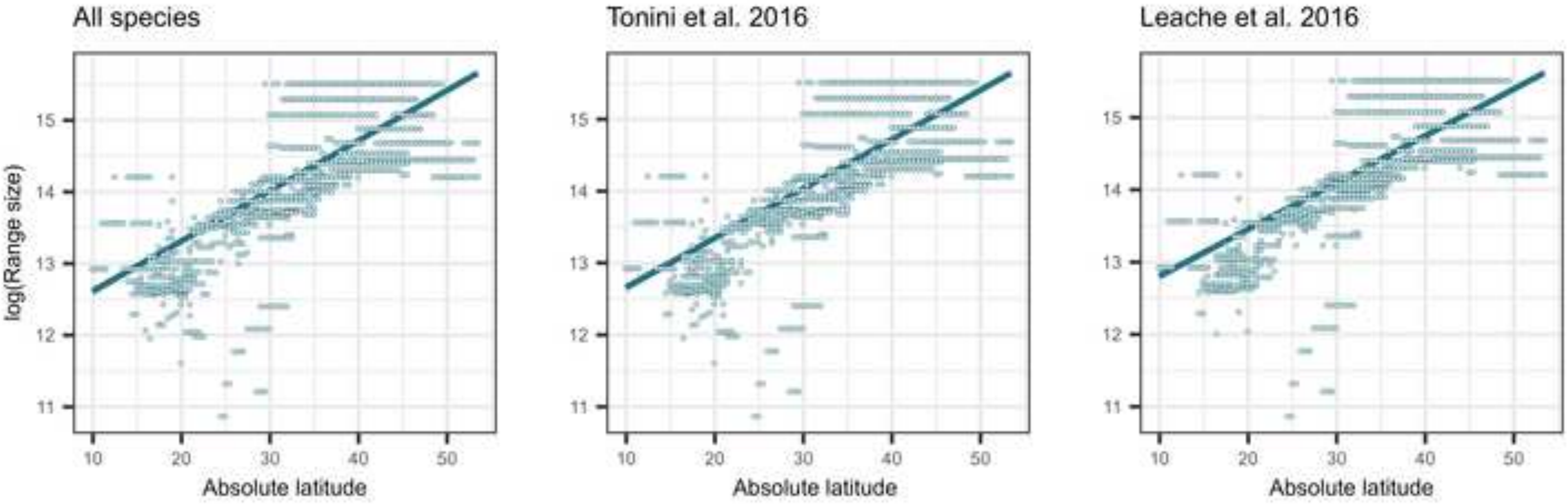

Fig4

Click here to access/download;Figure;Fig4.tiff

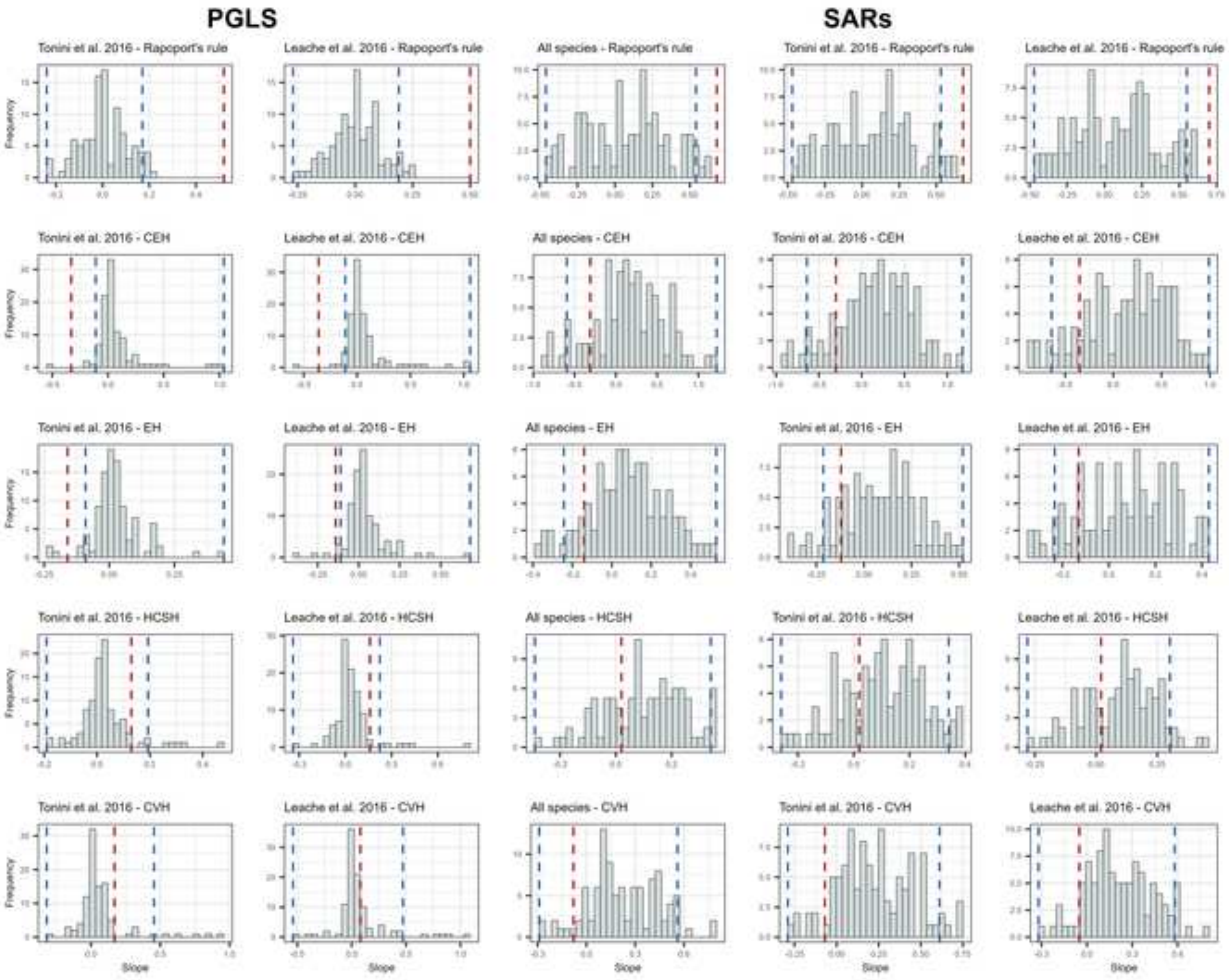

Click here to access/download
Supporting Information
S1_Appendix.pdf

Click here to access/download
Supporting Information
S2_Appendix.pdf

Click here to access/download
Supporting Information
S3_Appendix.pdf

Click here to access/download
Supporting Information
S4_Appendix.pdf

S2_Fig

Click here to access/download
Supporting Information
S2_Fig.pdf

S1_Table

Click here to access/download
Supporting Information
S1_Table.pdf

S1_Fig

Click here to access/download
Supporting Information
S1_Fig.pdf
